# Design and Synthesis of New 4-(3,4,5-Trimethoxyphenyl)Thiazole–Pyrimidine Derivatives as Potential Antiproliferative Agents

**DOI:** 10.3390/medicina59061076

**Published:** 2023-06-02

**Authors:** Ashraf K. El-Damasy, Heewon Jin, Mohamed A. Sabry, Hyun Ji Kim, Mohammed M. Alanazi, Seon Hee Seo, Eun-Kyoung Bang, Gyochang Keum

**Affiliations:** 1 Center for Brain Technology, Brain Science Institute, Korea Institute of Science and Technology (KIST), Seoul 02792, Republic of Korea; ph_karem2000@mans.edu.eg (A.K.E.-D.); hwjin37@snu.ac.kr (H.J.); hjkim926@kist.re.kr (H.J.K.); eunkbang@kist.re.kr (E.-K.B.); 2 Department of Medicinal Chemistry, Faculty of Pharmacy, Mansoura University, Mansoura 35516, Egypt; midoegy1992@hotmail.com; 3 Department of Pharmaceutical Chemistry, College of Pharmacy, King Saud University, Riyadh 11451, Saudi Arabia; mmalanazi@ksu.edu.sa; 4 Center for Brain Disorders, Brain Science Institute, Korea Institute of Science and Technology (KIST), Seoul 02792, Republic of Korea; shseo@kist.re.kr; 5 Division of Bio-Medical Science & Technology, KIST School, Korea University of Science and Technology (UST), Seoul 02792, Republic of Korea

**Keywords:** trimethoxyphenyl, thiazole, pyrimidines, NCI-60 screening, NSCL cancer cell line, ADME-Tox prediction

## Abstract

A new series of 3,4,5-trimethoxyphenyl thiazole pyrimidines has been synthesized and biologically evaluated for its in vitro anticancer activity. Compounds **4a**, **4b**, and **4h** with substituted piperazine showed the best antiproliferative activity. In the NCI-60 cell line screening, compound **4b** showed promising cytostatic activity against multiple cell lines. Notably, it elicited a GI value of 86.28% against the NSCL cancer cell line HOP-92 at a 10 μM dose. Compounds **4a** and **4h** at 10 μM showed promising GI values of 40.87% and 46.14% against HCT-116 colorectal carcinoma and SK-BR-3 breast cancer cell lines, respectively. ADME-Tox prediction of compounds **4a**, **4b,** and **4h** revealed their acceptable drug-likeness properties. In addition, compounds **4a**, **4b**, and **4h** showed a high probability of targeting kinase receptors via Molinspiration and Swiss TargetPrediction.

## 1. Introduction

Cancer is a malignant tumor caused by abnormal growth of malfunctioning tissues that may result from many factors, such as gene mutations or other environmental factors. There is still a massive need to develop new chemotherapeutic agents that target malignant tumors by various mechanisms and help inhibit the abnormal metastatic growth of malignant tumors [1,2].

Trimethoxyphenyl (TMP) moiety of colchicine (**I**) is a key pharmacophoric structure to bind tubulin, and it inhibits malignant cell division and growth [3,4,5]. From this point of view, many microtubule-targeting agents bearing TMP have been developed as potent tubulin inhibitors for treating cancers in the past decade [6,7,8,9,10,11,12,13,14]. The TMP thiazole derivatives compound **II** (TST) and compound **III** are potent anticancer candidates with promising growth inhibitory action against various cancer cell lines [15,16,17,18]. Dasatinib (**IV**) is a multi-kinase inhibitor anticancer drug with *N*-(2-methylpyrimidin-4-yl)thiazol-2-amine pharmacophore [19,20,21]. Compounds **V** and **VI**, benzo [4,5]thiazolo [3,2-a]pyrimidine derivatives, are potent anticancer candidates with promising IC_50_ values against a range of different cancer cell lines [22].

**Figure 1:**
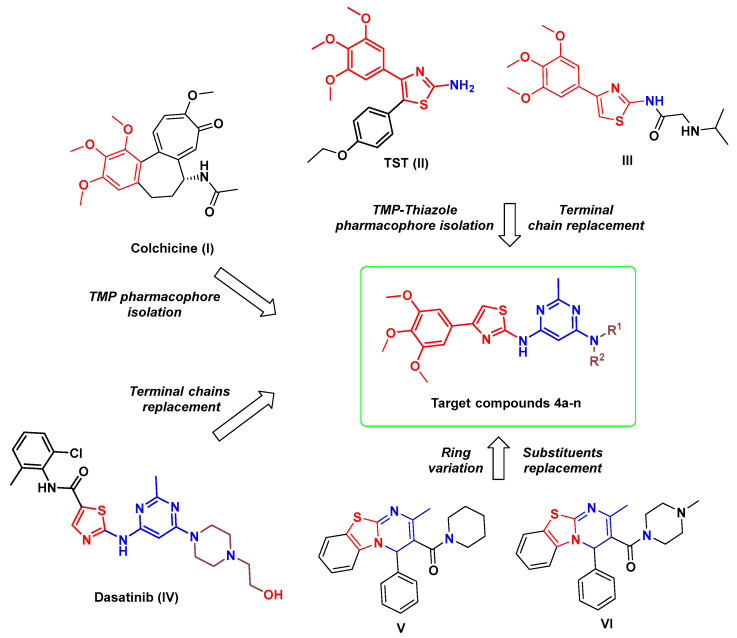
Rational design of the new trimethoxyphenyl thiazole derivatives.

In the present study, structural feature modifications on colchicine (I), compounds (**II**, **III**, **V**, and **VI**), and the FDA-approved drug dasatinib were performed to expand the chemical diversity of anticancer agents and generate new TMP thiazole derivatives with good anticipated anticancer activity. TMP pharmacophoric moiety was isolated from colchicine (**I**), and compounds **II** and **III** were modified by TMP thiazole pharmacophoric isolation and terminal groups’ replacement to obtain the newly designed TMP thiazole analogs. In addition, the central *N*-(2-methylpyrimidin-4-yl)thiazol-2-amine core of dasatinib (**IV**) was isolated by replacing its terminal chains to design the new TMP thiazole derivatives, while compounds **V** and **VI** were modified by varying the central ring and replacing their substituents with isolating their thiazole and pyrimidine rings to afford the newly designed TMP thiazole analogs. The main structural features of the designed series were the TMP thiazole moiety and pyrimidine ring linked to various terminal polar chains/nonpolar fragments (Figure 1). Several reports highlight the privilege of different cyclic amines in achieving favorable binding affinity with target proteins and cellular potency (via improving water solubility) [23,24,25,26,27,28]. Therefore, various amines (piperazines, morpholine, ethylenediamines, and propandiamines) were attached to the pyrimidine at the 6-position to explore their impact on anticancer activity and construct a reliable structure–activity relationship (SAR). The piperazine moiety was installed to the pyrimidine either directly (**4a**–**4f**) or through an ethyl/propylamine spacer (**4h** and **4k**).

## 2. Materials and Methods

### 2.1. General

Nuclear magnetic resonance (NMR) spectra of ^1^H and ^13^C were measured with a Bruker Avance spectrometer operating at 400 MHz for ^1^H NMR and 100 MHz for ^13^C NMR (Figure S1). The chemical shifts were recorded as parts per million (ppm, δ values) relative to the solvent standard. The signals were reported as s (singlet), d (doublet), t (triplet), q (quartet), quint (quintet), m (multiplet), and br. s (broad singlet), dd (doublet of doublet), or dt (doublet of triplet). The coupling constant (*J*) was expressed in Hertz (Hz). CDCl_3_, Acetone-*d*_6_, and DMSO-*d*_6_ were used as the NMR solvent from Cambridge Isotope Laboratories. Waters SYNAPT G2 mass spectrometer was used to get high-resolution mass spectra (HRMS) in ESI-TOF mode (Figure S2). High-performance liquid chromatography (HPLC) analysis was conducted using an Agilent 1100 model for certain representative compounds that showed purities ≥95% (Figure S3). YMC-Triart C18 column was used as a stationary phase; 150 × 2.0 mm I.D.; S-5 μm particle size; 120 Å pore size. The HPLC mobile phase increased linearly from 0.1% trifluoroacetic acid in distilled water to 0.1% trifluoroacetic acid in acetonitrile over 20 min at a flow rate of 1.0 mL/min. A chromatogram was obtained with a UV detector at 280 nm.

All chemical reagents and solvents, which are commercially available, were purchased from a commercial supplier, such as Sigma-Aldrich (St. Louis, MO, USA), Tokyo Chemical Industry Co. (Tokyo, Japan), Alfa Aesar (Haverhill, MA, USA), or Acros (Gujarat, India), and they were used without further purification. Reactions under the anhydrous system proceeded using inert argon gas. All reactions were monitored on thin-layer chromatography (TLC) plate (Merck, silica gel 60 F254, Darmstadt, Germany), and the components on TLC were visualized by observation using UV light (254 nm, 365 nm) and staining solutions. The types of staining solutions used for reaction monitoring were 20% ethanolic phosphomolybdic acid (PMA), 2% ninhydrin ethanolic solution, *p*-anisaldehyde, potassium permanganate solution, and cerium ammonium molybdate (CAM). Compounds **1** and **2** were prepared following the reported procedures references [29,30].

### 2.2. Procedure for the Synthesis of 2-Bromo-1-(3,4,5-trimethoxyphenyl)ethan-1-one (***1***) 

To a stirred solution of 1-(3,4,5-trimethoxyphenyl)ethan-1-one (2.5 g, 11.89 mmol) in anhydrous Et_2_O (30 mL), Br_2_ (0.73 mL, 14.27 mmol) was added dropwise at 0 °C. The reaction mixture temperature was gradually increased to room temperature for 2 h 35 min. After the reaction was finished, saturated aqueous NaHCO_3_ was added. Using a separation funnel, the aqueous layer was extracted with ethyl acetate (3 × 50 mL), and the organic layers were combined, washed with water and brine, then dried by Na_2_SO_4_ filtration. After the evaporation of ethyl acetate to concentrate resultants at room temperature without heating during the evaporation, the residues were triturated with n-hexane by scrubbing the resultants that were like brown gum. After 30 min, the brown gum was solidified. Then, hexane was removed by a pipette, and one more trituration was performed to afford the title compound as a brown solid; 2.883 g (84% yield); ^1^H NMR (400 MHz, CDCl_3_): δ 7.27 (s, 2H, Ar-H), 4.44 (s, 2H, Br-CH_2_), 3.96 (s, 3H, 4-O-CH_3_), 3.95 (s, 6H, 3,5-O-CH_3_); ^13^C NMR (100 MHz, CDCl_3_): δ 190.28 (C=O), 153.20 (Ar-C), 143.48(Ar-C), 129.01(Ar-C), 106.62 (Ar-C), 61.02 (4-O-CH_3_), 56.39 (3,5-O-CH_3_), 30.38 (Br-CH_2_); HRMS (ESI-TOF) *m*/*z* calcd. for C_11_H_14_BrO_4_ [M+H]^+^: 288.2901, found: 289.0075.

### 2.3. Procedure for the Synthesis of 4-(3,4,5-Trimethoxyphenyl)thiazol-2-amine (***2***) 

A solution of thiourea (399 mg, 5.230 mmol) in ethanol (5 mL) was added to a stirred solution of compound 1 (1.26 g, 4.358 mmol) in ethanol (10 mL). The reaction mixture was stirred under a reflux system at 78 °C for 45 min. After the reaction was completed, saturated aqueous NaHCO_3_ was added to make the reaction mixture basic with a pH value of 8 or 9. Then, using a separation funnel, the aqueous layer was extracted with dichloromethane (3 × 50 mL), and the organic layers were combined, washed with water and brine, and then dried by Na_2_SO_4_ filtration. After the evaporation of dichloromethane, the residue was stirred for 20 min with petroleum ether and filtered to purify the mixture. Flash column chromatography (FCC) was performed using 25–50% ethyl acetate in hexane to afford the title compound as a pure white solid; 1.14 g (98% yield); ^1^H NMR (400 MHz, CDCl_3_): δ 7.08 (s, 2H, 2,6-Ph-H), 6.65 (s, 1H, thiazole-H), 5.41 (s, 2H, NH_2_), 3.93 (s, 6H, 3,5- O-CH_3_), 3.89 (s, 3H, 4-O-CH_3_); ^13^C NMR (100 MHz, CDCl_3_): δ 167.11 (Ar-C), 153.33 (Ar-C), 151.19 (Ar-C), 137.97 (Ar-C), 130.51 (Ar-C), 103.34 (Ar-C), 102.38 (Ar-C), 60.94, (4-O-CH_3_), 56.18 (3,5-O-CH_3_); HRMS (ESI-TOF) *m*/*z* calcd. for C_12_H_15_N_2_O_3_S [M+H]^+^: 267.0815, found: 267.0803.

### 2.4. Procedure for the Synthesis of N-(6-Chloro-2-methylpyrimidin-4-yl)-4-(3,4,5-trimethoxyphenyl)thiazol-2-amine (***3***)

To a stirred suspension of compound **2** (100 mg, 0.375 mmol) in anhydrous THF (4 mL), 4,6-dichloro-2-methylpyrimidine (67.3 mg, 0.413 mmol) was added. Then, the reaction mixture was cooled down to 0 °C, and sodium hydride (18.9 mg, 0.788 mmol) was added, which was divided several times. The reaction mixture was stirred at room temperature for 16 h. After the reaction was finished, THF was evaporated, and the residue was purified by FCC using 20–50% ethyl acetate in hexane to afford the title compound as a white solid; 105.7 mg (72% yield); ^1^H NMR (400 MHz, DMSO-d_6_): δ 12.03 (br. s, 1H, NH), 7.64 (s, 1H, thiazole-H), 7.21 (s, 2H, 2,6-Ph-H), 6.92 (s, 1H, pyrimidin-5-yl-H), 3.86 (s, 6H, 3,5-O-CH_3_), 3.70 (s, 3H, 4-O-CH_3_), 2.55 (s, 3H, pyrimidine-CH_3_); ^13^C NMR (100 MHz, DMSO-d_6_): δ 167.78 (Ar-C), 158.63 (Ar-C), 158.38 (Ar-C), 158.25 (Ar-C), 153.54 (Ar-C), 149.33 (Ar-C), 137.73 (Ar-C), 130.49 (Ar-C), 108.62 (Ar-C), 103.43 (Ar-C), 103.33 (Ar-C), 60.52 (4-O-CH_3_), 56.32 (3,5- O-CH_3_), 25.61 (Ar-CH_3_); HRMS (ESI-TOF) *m*/*z* calcd. for C_17_H_18_ClN_4_O_3_S [M+H]^+^: 393.0801, found: 393.0788.

### 2.5. General Procedure for the Synthesis of N-(6-Substituted-2-methylpyrimidin-4-yl)-4-(3,4,5-trimethoxyphenyl)thiazole-2-amine (***4a***–***4n***)

A solution of secondary amines (0.510 mmol) in anhydrous dimethyl sulfoxide (1 mL) was added to a stirred solution of compound **3** (100 mg, 0.255 mmol) in anhydrous dimethyl sulfoxide (3 mL) and DIPEA (0.22 mL, 1.275 mmol). The reaction mixture was stirred at 110 °C for 2 h. After cooling to room temperature, water was added to quench the reaction. Using a separation funnel, the aqueous layer was extracted with ethyl acetate (4 × 50 mL), and the organic layers were combined, washed with water and brine, and then dried by Na_2_SO_4_ filtration. The solvent was evaporated under vacuum, and the resulting residue was purified by FCC using the proper eluent.

#### 2.5.1. *N*-(6-(4-Ethylpiperazin-1-yl)-2-methylpyrimidin-4-yl)-4-(3,4,5-trimethoxyphenyl)thiazol-2-amine (**4a**)

FCC eluent (50–100% ethyl acetate in hexane, then shifting to 10% methanol in ethyl acetate). White solid; 93.0 mg (82% yield); mp: 208–209 °C; ^1^H NMR (400 MHz, CDCl_3_): δ 11.61 (br. s, 1H, NH), 7.12 (s, 2H, 2,6-Ph-H), 7.11 (s, 1H, thiazole-H), 5.28 (s, 1H, pyrimidin-5-yl-H), 3.85 (s, 3H, 4-O-CH_3_), 3.81 (s, 6H, 3,5-O-CH_3_), 3.20 (br. s, 4H, 2 CH_2_-piperazine), 2.48 (s, 3H, pyrimidine-CH_3_), 2.40 (q, *J* = 7.1 Hz, 2H, CH_2_-CH_3_), 2.31 (br. s, 4H, 2 CH_2_-piperazine), 1.09 (t, *J* = 7.1 Hz, 3H, CH_2_-CH_3_); ^13^C NMR (100 MHz, CDCl_3_): δ 165.94 (Ar-C), 162.56 (Ar-C), 161.85 (Ar-C), 157.01 (Ar-C), 153.47 (Ar-C), 148.41 (Ar-C), 138.08 (Ar-C), 129.85 (Ar-C), 106.74 (Ar-C), 103.21 (Ar-C), 83.04 (Ar-C), 61.00 (4- O-CH_3_), 56.04 (3,5-O-CH_3_), 52.30 (2 CH_2_-piperazine), 52.19 (2 CH_2_-piperazine), 43.57 (Ar-CH_3_), 25.70 (CH_2_-CH_3_), 11.90 (CH_2_-CH_3_); HRMS (ESI-TOF) *m*/*z* calcd. for C_23_H_31_N_6_O_3_S [M+H]^+^: 471.2178, found: 471.2198.

#### 2.5.2. 2-(4-(2-Methyl-6-((4-(3,4,5-trimethoxyphenyl)thiazol-2-yl)amino)pyrimidin-4-yl)piperazin-1-yl)ethan-1-ol (**4b**)

FCC eluent (hexane/ethyl acetate, 2:1, then shifting to 1:1). White solid; 31.8 mg (30% yield); mp: 113–115 °C; ^1^H NMR (400 MHz, CDCl_3_): δ 11.54 (br. s, 1H, NH), 7.17 (s, 2H, 2,6-Ph-H), 7.08 (s, 1H, thiazole-H), 5.28 (s, 1H, pyrimidin-5-yl-H), 3.86 (s, 3H, 4-O-CH_3_), 3.82 (s, 6H, 3,5-O-CH_3_), 3.66 (t, *J* = 5.3 Hz, 2H, -CH_2_-CH_2_-OH), 3.19 (br. s, 4H, 2 CH_2_-piperazine), 2.71 (br. s, 1H, OH), 2.55 (t, *J* = 5.3 Hz, 2H, -CH_2_-CH_2_-OH), 2.50 (s, 3H, pyrimidine-CH_3_), 2.39 (t, *J* = 4.8 Hz, 4H, 2 CH_2_-piperazine); ^13^C NMR (100 MHz, CDCl_3_): δ 166.02 (Ar-C), 162.63 (Ar-C), 161.77 (Ar-C), 157.01 (Ar-C), 153.48 (Ar-C), 148.41 (Ar-C), 138.01 (Ar-C), 129.86 (Ar-C), 106.82 (Ar-C), 103.12 (Ar-C), 83.07 (Ar-C), 60.97 (4-O-CH_3_), 59.39 (-CH_2_-CH_2_-OH), 57.80 (-CH_2_-CH_2_-OH), 56.02 (3,5-O-CH_3_), 52.29(2 CH_2_-piperazine), 43.66 (2 CH_2_-piperazine), 25.68 (Ar-CH_3_); HRMS (ESI-TOF) *m*/*z* calcd. for C_23_H_31_N_6_O_4_S [M+H]^+^: 487.2127, found: 487.2122.

#### 2.5.3. *N*-(6-(4-(4-Methoxyphenyl)piperazin-1-yl)-2-methylpyrimidin-4-yl)-4-(3,4,5-trimethoxyphenyl)thiazol-2-amine (**4c**)

FCC eluent (hexane/ethyl acetate, 2:1, then shifting to 1:1). White solid; 31.8 mg (30% yield); mp: 216–217 °C; ^1^H NMR (400 MHz, CDCl_3_): δ 11.32 (br. s, 1H, NH), 7.21 (s, 2H, 2,6-Ph-H), 7.10 (s, 1H, thiazole-H), 6.94–6.87 (m, 4H, 2,3,5,6-Ph-H), 5.37 (s, 1H, pyrimidin-5-yl-H), 3.87 (s, 3H, 4-O-CH_3_), 3.86 (s, 6H, 3,5-O-CH_3_), 3.81 (s, 3H, 4-O-CH_3_), 3.38 (t, *J* = 4.8 Hz, 4H, 2 CH_2_-piperazine), 2.98 (t, *J* = 4.8 Hz, 4H, 2 CH_2_-piperazine), 2.54 (s, 3H, pyrimidine-CH_3_); ^13^C NMR (100 MHz, CDCl_3_): δ 166.12 (Ar-C), 162.66 (Ar-C), 161.65 (Ar-C), 157.06 (Ar-C), 154.18 (Ar-C), 153.56 (Ar-C), 148.54 (Ar-C), 145.53 (Ar-C), 138.17 (Ar-C), 129.92 (Ar-C), 118.67 (Ar-C), 114.51 (Ar-C), 106.77 (Ar-C), 103.22 (Ar-C), 83.03 (Ar-C), 60.99 (4-O-CH_3_), 56.09 (4-O-CH_3_), 55.58 (3,5-O-CH_3_), 50.48 (2 CH_2_-piperazine), 43.81 (2 CH_2_-piperazine), 25.71 (Ar-CH_3_); HRMS (ESI-TOF) *m*/*z* calcd. for C_28_H_33_N_6_O_4_S [M+H]^+^: 549.2284, found: 549.2293.

#### 2.5.4. *N*-(6-(4-(4-Fluorophenyl)piperazin-1-yl)-2-methylpyrimidin-4-yl)-4-(3,4,5-trimethoxyphenyl)thiazol-2-amine (**4d**)

FCC eluent (hexane/ethyl acetate, 1:2). White solid; 107.3 mg (79%); mp: 84–86 °C; ^1^H NMR (400 MHz, DMSO-d_6_): δ 11.31 (br. s, 1H, NH), 7.49 (s, 1H, thiazole-H), 7.20 (s, 2H, 2,6-Ph-H), 7.12–7.07 (m, 2H, 2,6-Ph-H), 7.04–7.00 (m, 2H, 3,5-Ph-H), 6.18 (s, 1H, pyrimidin-5-yl-H), 3.85 (s, 6H, 3,5-O-CH_3_), 3.70 (s, 3H, 4-O-CH_3_), 3.68 (br. s, 4H, 2 CH_2_-piperazine), 3.18 (br. s, 4H, 2 CH_2_-piperazine), 2.43 (s, 3H, pyrimidine-CH_3_); ^13^C NMR (100 MHz, DMSO-d_6_): δ 165.64 (Ar-C), 162.83 (Ar-C), 159.67 (Ar-C), 158.15 (Ar-C), 155.55 (Ar-C), 153.51 (Ar-C), 148.95 (Ar-C), 148.21 (Ar-C), 137.58 (Ar-C), 130.88 (Ar-C), 118.07 (q, *J* = 7.6 Hz) (Ar-C), 115.81 (q, *J* = 22 Hz) (Ar-C), 107.44 (Ar-C), 103.39 (Ar-C), 82.72 (Ar-C), 60.54 (4-O-CH_3_), 56.34 (3,5-O-CH_3_), 49.30 (2 CH_2_-piperazine), 43.91 (2 CH_2_-piperazine), 26.08 (Ar-CH_3_); HRMS (ESI-TOF) *m*/*z* calcd. for C_27_H_30_FN_6_O_3_S [M+H]^+^: 537.2084, found: 537.2102.

#### 2.5.5. *N*-(6-(4-(4-Chlorophenyl)piperazin-1-yl)-2-methylpyrimidin-4-yl)-4-(3,4,5-trimethoxyphenyl)thiazol-2-amine (**4e**)

FCC eluent (hexane/ethyl acetate, 2:1, *v*/*v*). White solid; 73.5 mg (52% yield); mp: 125–130 °C; ^1^H NMR (400 MHz, CDCl_3_): δ 11.94 (br. s, 1H, NH), 7.22 (s, 1H, thiazole-H), 7.13–6.73 (m, 6H, 6 Ph-H), 5.88 (s, 1H, pyrimidin-5-yl-H), 3.94–3.92 (m, 2H, CH_2_-piperazine), 3.88 (br. s, 4H, 2 CH_2_-piperazine), 3.86 (s, 9H, 3,4,5-O-CH_3_), 2.67 (s, 3H, pyrimidine-CH_3_); ^13^C NMR (100 MHz, CDCl_3_): δ 167.73 (Ar-C), 160.42 (Ar-C), 159.34 (Ar-C), 157.17 (Ar-C), 153.79 (Ar-C), 149.65 (Ar-C), 145.29 (Ar-C), 138.60 (Ar-C), 132.03 (Ar-C), 129.98 (Ar-C), 118.62 (Ar-C), 113.60 (Ar-C), 108.05 (Ar-C), 103.99 (Ar-C), 103.08 (Ar-C), 61.02 (4-O-CH_3_), 56.23 (3,5-O-CH_3_), 42.31 (2 CH_2_-piperazine), 40.82 (2 CH_2_-piperazine), 25.44 (Ar-CH_3_); HRMS (ESI-TOF) *m*/*z* calcd. for C_27_H_30_ClN_6_O_3_S [M+H]^+^: 553.1788, found: 553.1786.

#### 2.5.6. *N*-(6-(4-(2,3-Dichlorophenyl)piperazin-1-yl)-2-methylpyrimidin-4-yl)-4-(3,4,5-trimethoxyphenyl)thiazol-2-amine (**4f**)

FCC eluent (hexane/ethyl acetate, 3:1, then shifting to 1:1). White solid; 80.3 mg (53% yield); mp: 202–205 °C; ^1^H NMR (400 MHz, CDCl_3_): δ 11.07 (br. s, 1H, NH), 7.24–7.08 (m, 6H, 6 Ph-H), 6.97 (dd, *J* = 7.5, 2.1 Hz, 1H, 2,3-Cl-Ph-4-H), 3.88 (s, 3H, 4-O-CH_3_), 3.85 (s, 6H, 3,5-O-CH_3_), 3.47 (br. s, 4H, 2 CH_2_-piperazine), 2.98 (t, *J* = 4.7 Hz, 4H, 2 CH_2_-piperazine), 2.54 (s, 3H, pyrimidine-CH_3_); ^13^C NMR (100 MHz, CDCl_3_): δ 167.68 (Ar-C), 162.96 (Ar-C), 160.70 (Ar-C), 157.86 (Ar-C), 153.53 (Ar-C), 151.20 (Ar-C), 150.92 (Ar-C), 139.65 (Ar-C), 134.20 (Ar-C), 130.02 (Ar-C), 127.58 (Ar-C), 125.07 (Ar-C), 124.97 (Ar-C), 118.71 (Ar-C), 106.79 (Ar-C), 103.17 (Ar-C), 83.23 (Ar-C), 60.99 (4-O-CH_3_), 56.07 (3,5-O-CH_3_), 50.95 (2 CH_2_-piperazine), 43.97 (2 CH_2_-piperazine), 25.70 (Ar-CH_3_); HRMS (ESI-TOF) *m*/*z* calcd. for C_27_H_29_Cl_2_N_6_O_3_S [M+H]^+^: 587.1399, found: 587.1414.

#### 2.5.7. *N*-(2-Methyl-6-morpholinopyrimidin-4-yl)-4-(3,4,5-trimethoxyphenyl)thiazol-2-amine (**4g**)

The title compound was produced as a pure white solid; 85.9 mg (76% yield); mp: 110–112 °C; ^1^H NMR (400 MHz, CDCl_3_): δ 11.70 (br. s, 1H, NH), 7.16 (s, 2H, 2,6-Ph-H), 7.08 (s, 1H, thiazole-H), 5.23 (s, 1H, pyrimidin-5-yl-H), 3.87 (s, 3H, 4-O-CH_3_), 3.81 (s, 6H, 3,5-O-CH_3_), 3.59 (t, *J* = 4.7 Hz, 4H, 2 CH_2_-morpholine), 3.13 (t, *J* = 4.7 Hz, 4H, 2 CH_2_-morpholine), 2.51 (s, 3H, pyrimidine-CH_3_); ^13^C NMR (100 MHz, CDCl_3_): δ 165.99 (Ar-C), 162.83 (Ar-C), 161.86 (Ar-C), 156.99 (Ar-C), 153.53 (Ar-C), 148.43 (Ar-C), 138.07 (Ar-C), 129.85 (Ar-C), 106.91 (Ar-C), 103.15 (Ar-C), 83.04 (Ar-C), 66.35 (2 CH_2_-morpholine), 60.96 (4-O-CH_3_), 56.02 (3,5-O-CH_3_), 43.95 (2 CH_2_-morpholine), 25.64 (Ar-CH_3_); HRMS (ESI-TOF) *m*/*z* calcd. for C_21_H_26_N_5_O_4_S [M+H]^+^: 444.1706, found: 444.1711.

#### 2.5.8. 2-Methyl-*N*^4^-(2-(4-methylpiperazin-1-yl)ethyl)-*N*^6^-(4-(3,4,5-trimethoxyphenyl)thiazol-2-yl)pyrimidine-4,6-diamine (**4h**)

FCC eluent (50–100% ethyl acetate in hexane, then shifting to 10% methanol in ethyl acetate, and then 10% methanol in dichloromethane). White solid; 77.6 mg (68% yield); mp: 213–219 °C; ^1^H NMR (400 MHz, CDCl_3_): δ 11.64 (br. s, 1H, NH), 7.12 (s, 2H, 2,6-Ph-H), 7.03 (s, 1H, thiazole-H), 5.35 (br. s, 1H, NH), 5.02 (s, 1H, pyrimidin-5-yl-H), 3.80 (s, 3H, 4-O-CH_3_), 3.77 (s, 6H, 3,5-O-CH_3_), 2.57–2.40 (m, 15H, 4 CH_2_-piperazine, N-CH_2_-CH_2_-N, N-CH_3_), 2.28(s, 3H, pyrimidine-CH_3_); ^13^C NMR (100 MHz, CDCl_3_): δ 166.05 (Ar-C), 162.56 (Ar-C), 161.55 (Ar-C), 156.89 (Ar-C), 153.42 (Ar-C), 148.65 (Ar-C), 137.98 (Ar-C), 129.98 (Ar-C), 106.74 (Ar-C), 103.24 (Ar-C), 81.71 (Ar-C), 60.89 (4-O-CH_3_), 55.95 (3,5-O-CH_3_), 55.85 (N-CH_2_-CH_2_-N), 54.85 (N-CH_2_-CH_2_-N), 52.39 (N-CH_3_), 45.81 (2 CH_2_-piperazine), 37.40 (2 CH_2_-piperazine), 25.34 (Ar-CH_3_); HRMS (ESI-TOF) *m*/*z* calcd. for C_24_H_34_N_7_O_3_S [M+H]^+^: 500.2444, found: 500.2452.

#### 2.5.9. 2-Methyl-*N*^4^-(2-morpholinoethyl)-*N*^6^-(4-(3,4,5-trimethoxyphenyl)thiazol-2-yl)pyrimidine-4,6-diamine (**4i**)

FCC eluent (50–100% ethyl acetate in hexane, then shifting to 10% methanol in ethyl acetate). White solid; 73.5 mg (59% yield); mp: 200–202 °C; ^1^H NMR (400 MHz, CDCl_3_): δ 11.39 (br. s, 1H, NH), 7.10 (s, 2H, 2,6-Ph-H), 7.01 (s, 1H, thiazole-H), 5.44 (br. s, 1H, NH), 5.12 (s, 1H, pyrimidin-5-yl-H), 3.81 (s, 3H, 4-O-CH_3_), 3.79 (s, 6H, 3,5-O-CH_3_), 3.65 (t, *J* = 4.0 Hz, 4H, 2 CH_2_-morpholine), 2.68 (br. s, 2H, CH_2_-morpholine), 2.46 (s, 3H, pyrimidine-CH_3_), 2.42–2.37 (m, 6H, N-CH_2_-CH_2_-N, CH_2_-morpholine); ^13^C NMR (100 MHz, CDCl_3_): δ 166.12 (Ar-C), 162.62 (Ar-C), 161.52 (Ar-C), 156.89 (Ar-C), 153.46 (Ar-C), 148.75 (Ar-C), 138.05 (Ar-C), 129.99 (Ar-C), 106.76 (Ar-C), 103.33 (Ar-C), 81.83 (Ar-C), 66.83 (2 CH_2_-morpholine), 60.92 (4-O-CH_3_), 56.44 (N-CH_2_-CH_2_-N), 56.01 (3,5-O-CH_3_), 53.14 (N-CH_2_-CH_2_-N), 37.20 (2 CH_2_-morpholine), 25.26 (Ar-CH_3_); HRMS (ESI-TOF) *m*/*z* calcd. for C_23_H_31_N_6_O_4_S [M+H]^+^: 487.2127, found: 487.2135.

#### 2.5.10. 2-((2-Methyl-6-((4-(3,4,5-trimethoxyphenyl)thiazol-2-yl)amino)pyrimidin-4-yl)amino)ethan-1-ol (**4j**)

FCC eluent (100% ethyl acetate). Pale yellow solid; 97.8 mg (92% yield); mp: 85–90 °C; ^1^H NMR (400 MHz, Acetone-d_6_): δ 10.14 (br. s, 1H, NH), 7.31 (s, 1H, thiazole-H), 7.24 (s, 2H, 2,6-Ph-H), 6.31 (br. s, 1H, NH), 6.11 (s, 1H, pyrimidin-5-yl-H), 5.64 (br. s, 1H, OH), 3.89 (s, 6H, 3,5-O-CH_3_), 3.76–3.74 (m, 5H, 4-O-CH_3_ and CH_2_-OH), 3.48 (br. s, 2H. CH_2_-NH), 2.43 (s, 3H, pyrimidine-CH_3_); ^13^C NMR (100 MHz, CDCl_3_): δ 166.06 (Ar-C), 162.84 (Ar-C), 161.31 (Ar-C), 156.68 (Ar-C), 153.48 (Ar-C), 148.82 (Ar-C), 138.00 (Ar-C), 130.32 (Ar-C), 106.96 (Ar-C), 103.59 (Ar-C), 82.60 (Ar-C), 61.01 (4-O-CH_3_), 56.14 (3,5-O-CH_3_), 53.45 (CH_2_-OH), 43.89 (CH_2_-NH), 25.16 (Ar-CH_3_); HRMS (ESI-TOF) *m*/*z* calcd. for C_19_H_24_N_5_O_4_S [M+H]^+^: 418.1549, found: 418.1559.

#### 2.5.11. 2-Methyl-*N*^4^-(3-(4-methylpiperazin-1-yl)propyl)-*N*^6^-(4-(3,4,5-trimethoxyphenyl)thiazol-2-yl)pyrimidine-4,6-diamine (**4k**)

FCC eluent (hexane/ethyl acetate, 1:2, then which was shifted to 10% methanol in dichloromethane with a few drops of ammonia). Pale yellow solid; 38.4 mg (74% yield); mp: 89–91 °C; ^1^H NMR (400 MHz, CDCl_3_): δ 11.01 (br. s, 1H, NH), 7.13 (s, 2H, 2,6-Ph-H), 7.03 (s, 1H, thiazole-H), 5.50 (t, *J* = 5.2 Hz, 1H, CH_2_-NH), 5.18 (s, 1H, pyrimidin-5-yl-H), 3.84 (s, 3H, 4-O-CH_3_), 3.83 (s, 6H, 3,5-O-CH_3_), 2.83 (br. s, 2H, CH_2_-piperazine), 2.57–2.41 (m, 11H, 3 CH_2_-piperazine, -CH_2_-CH_2_-CH_2_-, N-CH_3_), 2.37 (t, *J* = 6.8 Hz, 2H, -CH_2_-CH_2_-NH), 2.30 (s, 3H, pyrimidine-CH_3_), 1.60 (t, *J* = 6.6 Hz, 2H, -CH_2_-CH_2_-N-); ^13^C NMR (100 MHz, CDCl_3_): δ 166.26 (Ar-C), 163.03 (Ar-C), 161.18 (Ar-C), 156.88 (Ar-C), 153.43 (Ar-C), 148.80 (Ar-C), 138.03 (Ar-C), 130.12 (Ar-C), 106.63 (Ar-C), 103.31 (Ar-C), 81.55 (Ar-C), 60.92 (4-O-CH_3_), 56.31 (N-CH_3_), 56.04 (3,5-O-CH_3_), 55.05 (-CH_2_-CH_2_-NH), 53.07 (-CH_2_-CH_2_-N-), 45.92 (2 CH_2_-piperazine), 40.29 (2 CH_2_-piperazine), 25.52 (Ar-CH_3_), 25.41 (-CH_2_-CH_2_-CH_2_-); HRMS (ESI-TOF) m/z calcd. for C_25_H_36_N_7_O_3_S [M+H]^+^: 514.2600, found: 514.2597.

#### 2.5.12. 2-Methyl-*N*^4^-(3-morpholinopropyl)-*N*^6^-(4-(3,4,5-trimethoxyphenyl)thiazol-2-yl)pyrimidine-4,6-diamine2 (**4l**)

FCC eluent (10% methanol in dichloromethane). White solid; 88.9 mg (68% yield); mp: 170–177 °C; ^1^H NMR (400 MHz, CDCl_3_): δ 11.41 (br. s, 1H, NH), 7.12 (s, 2H, 2,6-Ph-H), 7.04 (s, 1H, thiazole-H), 5.63 (br. s, 1H, NH), 5.12 (s, 1H, pyrimidin-5-yl-H), 3.81 (s, 3H, 4-O-CH_3_), 3.79 (s, 6H, 3,5-O-CH_3_), 3.65 (t, *J* = 4.2 Hz, 4H, 2 CH_2_-morpholine), 2.76 (br. s, 2H, CH_2_- morpholine), 2.46 (s, 3H, pyrimidine-CH_3_), 2.36–2.28 (m, 6H, CH_2_-morpholine, -CH_2_-CH_2_-N, -CH_2_-CH_2_-NH), 1.54 (t, *J* = 6.3 Hz, 2H, CH_2_-CH_2_-CH_2_-); ^13^C NMR (100 MHz, CDCl_3_): δ 166.18 (Ar-C), 162.99 (Ar-C), 161.44 (Ar-C), 156.90 (Ar-C), 153.42 (Ar-C), 148.65 (Ar-C), 138.00 (Ar-C), 130.02 (Ar-C), 106.67 (Ar-C), 103.28 (Ar-C), 81.56 (Ar-C), 66.90 (2 CH_2_-morpholine), 60.90 (4-O-CH_3_), 56.01 (3,5-O-CH_3_), 53.64 (-CH_2_-CH_2_-NH), 40.90 (-CH_2_-CH_2_-N-), 40.16 (2 CH_2_-morpholine), 25.40(Ar-CH_3),_ 25.11 (CH_2_-CH_2_-CH_2_); HRMS (ESI-TOF) *m*/*z* calcd. for C_24_H_33_N_6_O_4_S [M+H]^+^: 501.2284, found: 501.2295.

#### 2.5.13. *N*^4^-(3-(Dimethylamino)propyl)-2-methyl-*N*^6^-(4-(3,4,5-trimethoxyphenyl)thiazol-2-yl)pyrimidine-4,6-diamine (**4m**)

FCC eluent (10% methanol in ethyl acetate, then changed to 10% methanol in dichloromethane with 4 drops of ammonia). White solid; 66.6 mg (82% yield); mp: 86–88 °C; ^1^H NMR (400 MHz, CDCl_3_): δ 11.29 (br. s, 1H, NH), 7.14 (s, 2H, 2,6-Ph-H), 7.04 (s, 1H, thiazole-H), 5.51 (br. s, 1H, NH), 5.14 (s, 1H, pyrimidin-5-yl-H), 3.84 (s, 3H, 4-O-CH_3_), 3.82 (s, 6H, 3,5-O-CH_3_), 2.76 (br. s, 2H_,_ -CH_2_-CH_2_-NH), 2.48 (s, 3H, pyrimidine-CH_3_), 2.27 (t, *J* = 6.7 Hz, 2H, -CH_2_-CH_2_-N-), 2.18 (s, 6H_,_ 2 N-CH_3_), 1.56 (quint, *J* = 6.6 Hz, 2H, CH_2_-CH_2_-CH_2_); ^13^C NMR (100 MHz, CDCl_3_): δ 166.16 (Ar-C), 163.04 (Ar-C), 161.37 (Ar-C), 156.91 (Ar-C), 153.44 (Ar-C), 148.74 (Ar-C), 138.00 (Ar-C), 130.10 (Ar-C), 106.64 (Ar-C), 103.29 (Ar-C), 81.54 (Ar-C), 60.92 (4-O-CH_3_), 57.59 (-CH_2_-CH_2_-NH), 56.01 (3,5-O-CH_3_), 45.42 (2 N-CH_3_), 40.23 (-CH_2_-CH_2_-N-), 26.35 (Ar-CH_3_), 25.40 (CH_2_-CH_2_-CH_2_); HRMS (ESI-TOF) *m*/*z* calcd. for C_22_H_31_N_6_O_3_S [M+H]^+^: 459.2178, found: 459.2193.

#### 2.5.14. 3-((2-Methyl-6-((4-(3,4,5-trimethoxyphenyl)thiazol-2-yl)amino)pyrimidin-4-yl)amino)propan-1-ol (**4n**)

FCC eluent (100% ethyl acetate). Pale yellow solid; 38.2 mg (70% yield); mp: 97–99 °C; ^1^H NMR (400 MHz, CDCl_3_): δ 11.08 (br. s, 1H, NH), 7.13 (s, 2H, 2,6-Ph-H), 7.03 (s, 1H, thiazole-H), 5.13 (s, 1H, pyrimidin-5-yl-H), 4.98 (br. s, 1H, NH), 3.85 (s, 3H, 4-O-CH_3_), 3.84 (s, 6H, 3,5-O-CH_3_), 3.60 (t, *J* = 5.4 Hz, 2H_,_ -CH_2_-CH_2_-NH), 3.11 (br. s, 2H, -CH_2_-CH_2_-N-), 2.48 (s, 3H, pyrimidine-CH_3_), 1.64 (quint, *J* = 5.7 Hz, 2H, CH_2_-CH_2_-CH_2_); ^13^C NMR (100 MHz, CDCl_3_): δ 166.20 (Ar-C), 163.06 (Ar-C), 161.32 (Ar-C), 156.65 (Ar-C), 153.44 (Ar-C), 148.85 (Ar-C), 138.08 (Ar-C), 130.28 (Ar-C), 106.85 (Ar-C), 103.60 (Ar-C), 82.52 (Ar-C), 60.96 (4-O-CH_3_), 59.20 (-CH_2_-CH_2_-NH), 56.14 (3,5-O-CH_3_), 38.11 (-CH_2_-CH_2_-N-), 32.13 (Ar-CH_3_), 25.26 (CH_2_-CH_2_-CH_2_); HRMS (ESI-TOF) *m*/*z* calcd. for C_20_H_26_N_5_O_4_S [M+H]^+^: 432.1706, found: 432.1707.

### 2.6. NCI-60 Screening

The anticancer screening of compounds **4b**, **4c**, **4k**, and **4n** over a panel of six human leukemia cell lines was conducted using the Sulforhodamine B (SRB) assay (Figure S4) at the National Cancer Institute (NCI), Bethesda, Maryland, USA, employing the standard protocol [31].

### 2.7. In Vitro Anticancer Screening (MTT Assay)

Four human cancer cell lines, MCF-7 and SK-BR-3 (breast cancer cell lines), HCT-116 (human colorectal carcinoma), K562 (leukemia cell lines), and the normal cell line L132 were used in the in vitro MTT assay. MTT evaluation was performed to examine the antiproliferative activity of compounds **4a**–**4n** following the reported procedures [32].

## 3. Results and Discussion

### 3.1. Chemistry

**Scheme 1:**
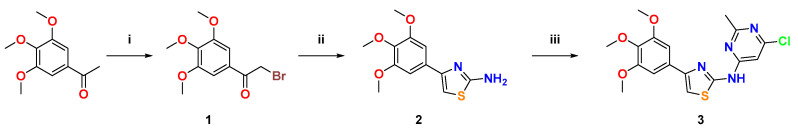
Reagent and reaction conditions: (i) Br_2_, Et_2_O, 0 °C to rt, 2 h 35 min, 84%; (ii) thiourea, EtOH, reflux, 45 min, 98%; (iii) 4,6-dichloro-2-methylpyrimidine, THF, 0 °C to rt, 16 h, 72%.

The key intermediate, *N*-(6-chloro-2-methylpyrimidin-4-yl)-4-(3,4,5-trimethoxyphenyl)thiazol-2-amine (**3**), was prepared as shown in Scheme 1. First, 2-bromo-1-(3,4,5-trimethoxyphenyl)ethan-1-one (**1**) **[29]** was synthesized via bromination of 3,4,5-trimethoxy acetophenone. Then, compound **1** was refluxed with thiourea for 45 min to yield compound **2 [30]**. Next, the key intermediate **3** was achieved by reacting compound **2** with 4,6-dichloro-2-methylpyrimidine using sodium hydride as a base with a 72% yield.

**Scheme 2:**
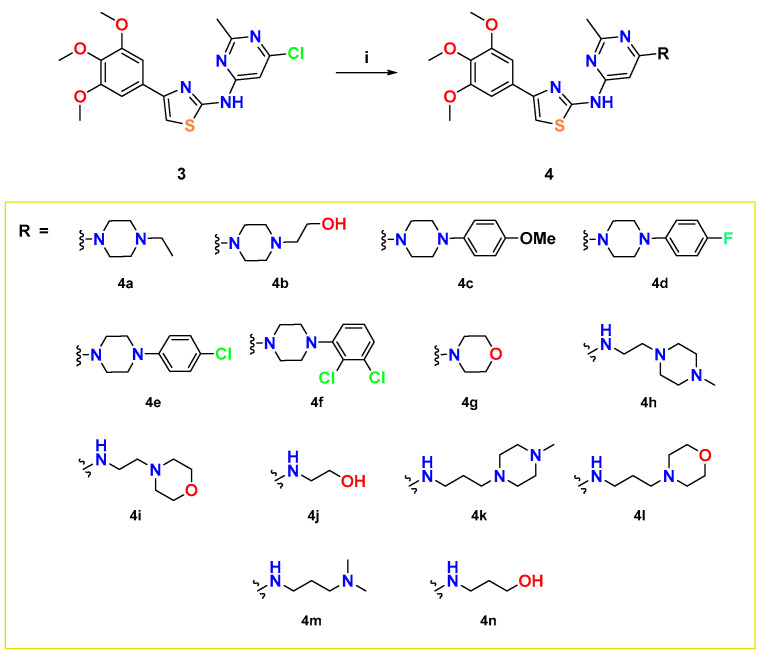
Reagents and reaction conditions: (i) substituted amine, DIPEA, DMSO, 110 °C, 1–15 h, 30–92%.

The installation of various amine groups to **3** was accomplished through a nucleophilic substitution reaction with substituted amines using *N,N*-diisopropylethylamine (DIPEA) as a base in DMSO at 110 °C to afford **4a**–**4n** (Scheme 2). All final compounds were obtained with high or moderate yield except **4b** and **4k**.

### 3.2. Antiproliferative Activities against NCI-60 Cell Line Panel at Single Dose Testing

The chemical structures of the newly synthesized compounds **4a**–**4n** were submitted to NCI for screening their antiproliferative activities [33]. Based on the degree of structural diversity and computational analysis, NCI selected compounds **4b**, **4c**, **4k**, and **4n** from the series to investigate their anticancer activity at a 10 μM dose (Figure S4). The results showed that compound **4b** has the highest cytotoxicity with a mean growth inhibition (GI) value of 32.20% (Table 1). The mean GI values of compounds **4c**, **4k**, and **4n** were 21.40%, 19.40%, and 16.44%, respectively. The superior activity of **4b** over the other tested compounds may be due to the direct attachment of the piperazine ring to the pyrimidine central core with some extent of flexibility in its terminal chain-bearing polar hydroxyl group. Moreover, compound **4b** showed GI values higher than 56% in eight sub-panels, as shown in Table 1.

The highest GI exerted by **4b** was 86.28%, found in the NSCL cancer cell line HOP-92, indicating the most potent anticancer activity among the four tested compounds. Regarding other tested cell lines, compound **4b** showed GI values of 61.31%, 59.05%, 73.52%, 57.10%, 72.38%, 56.66%, and 70.54% against SR, HT29, SF-295, LOX IMVI, A498, HS 578T, and T-47D cancer cell lines, respectively.

**Figure 2:**
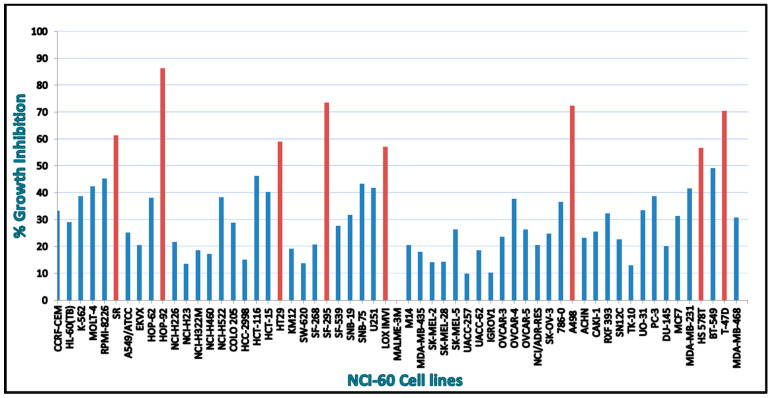
Percent (%) growth inhibition of the most potent compound **4b** against NCI-60 cell lines.

The antiproliferative activity of compound **4b** against the full NCI-60 cell line panel at 10 μM is shown in Figure 2. From these findings, it can be concluded that the best antiproliferative activity of compound **4b** was against lung cancer HOP-92, CNS cancer SF-295, renal cancer A498, and breast cancer T-47D cell lines, and to a lesser extent against leukemia SR, colon cancer HT29, melanoma LOX IMVI, and breast cancer HS 578T cell lines.

Among the other examined compounds, as shown in Table 1, compounds **4c** and **4k** showed moderate anticancer activity that may stem from the piperazine moiety in their terminal side chain with a different configuration compared to **4b**. Compound **4c** showed GI higher than 50% over four cancer cell lines. The best cytotoxic activity was against breast cancer cell line T-47D at 62.98%. In addition, the propandiamine **4k** showed GI values higher than 50% against four cancer cell lines. The best cytotoxic activity elicited by **4k** was against NSCL cancer cell line HOP-92 by 64.64%.

On the other hand, the propanolamine **4n** showed the least potent anticancer activity among the tested compounds, and this may be due to the absence of the piperazine ring that is present in the three other tested compounds. However, **4n** showed selective cytotoxicity against the T-47D breast cancer cell line with a GI% value of 68.39%.

### 3.3. In Vitro Anticancer MTT Assay against Four Cell Lines

Compounds **4a**–**4n** were subjected to MTT assay [34] to test their antiproliferative activity against four human cancer cell lines, i.e., MCF-7 and SK-BR-3 (breast cancer cells), HCT-116 (human colorectal carcinoma), and K562 (leukemia cells, chronic myelogenous), by the MTT assay method. In the MTT assay, the antiproliferative assay was focused on breast cancer cell lines as it showed the best results in the NCI-60 assay. All tested compounds were tested at 100 and 10 μM. The results are expressed as the percentage of growth inhibition (GI%), using the multi-kinase inhibitor sorafenib as a reference anticancer drug (Table 2).

The results reveal that all designed compounds had comparable cytotoxic activity against the four human cancer cell lines at 100 μM. Among the tested compounds, nine compounds (**4a**, **4b**, and **4h**–**4n**) manifested more than 70% GI over all tested cancer cell lines, and 27 cases were above 90% GI at 100 μM. Focusing on the concentration of 10 μM, the ethyl piperazine compounds **4a** and **4h** showed the best GI% of 40.87 and 46.14 against HCT-116 and SK-BR-3 cell lines, respectively. These GI% values are comparable to sorafenib (48.41 and 48.87 against HCT-116 and SK-BR-3, respectively). Both **4a** and **4h** compounds possess a piperazine ring in their terminal chain with slight flexibility. Considering the activity at 100 μM and 10 μM doses, it is found that compounds **4c**–**4f** with a substituted phenyl moiety at the terminal *N* of piperazine have a relatively low percentage of GI (<60% at 100 μM and <40% at 10 μM). In addition, it was noted that the variation in substituents on the phenyl ring in compounds **4c**–**4f** did not impact the anticancer activity. These cellular findings may indicate that the compounds derived from this series should have some flexibility in their terminal configuration to exert a considerable anticancer effect. The morpholine derivative **4g** showed an antiproliferative pattern similar to that observed with phenylpiperazines **4c**–**4f**. Moreover, the ethylamines **4h**-**4i** manifested comparable activity with their corresponding propylamines **4k**, **4l**, and **4n**, which reveals that both ethyl and propyl spacers are tolerable.

Moreover, compounds **4a** and **4h** showed weak cytotoxic effects over L132 normal cells, where they exerted high GI_50_ values of 23.52 and 26.38 μM, respectively (Table 3). These cellular outcomes reveal the selective antiproliferative activity of compounds **4a** and **4h** toward cancer cells.

### 3.4. Structure Similarity Search

The most potent compounds, **4a**, **4b**, and **4h,** were subjected to a structure similarity search (Molinspiration and Swiss TargetPredictors) to acquire certain insights about their potential molecular targets. Molinspiration concentrates on certain classes of drugs and calculates the specific activity scores for these classes. These bioactivity scores may be a proper measure to predict the target with the highest probability for the most potent synthesized compounds [35].

A Molinspiration bioactivity score greater than zero indicates a high probability of being active against this target. The obtained Molinspiration data (Table 4) for the most active anticancer compounds **4a**, **4b**, and **4h** revealed their high structural similarity with kinase inhibitors class with scores varying from 0.30 to 0.38 and to a lesser extent G-protein coupled receptor (GPCR) ligands with scores ranging from 0.08 to 0.10. These suggest that compounds **4a**, **4b**, and **4h** may exert their anticancer activity by inhibiting certain oncogenic kinases or targeting GPCR.

The Swiss TargetPrediction server was used to perform ligand-based target prediction of the precise targets of compounds [36]. It was utilized to identify the possible drug targets of the most active anticancer compounds **4a**, **4b**, and **4h**. The target prediction was limited to Homo sapiens targets to identify the targets belonging to human sources only.

**Figure 3:**
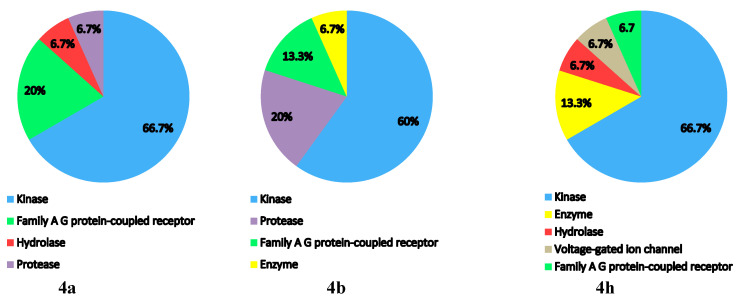
Percent (%) probability of targets predicted by Swiss TargetPrediction for the most active compounds **4a**, **4b**, and **4h**.

The acquired data, shown in Figure 3, predicted that the targets with the highest probability percentages were kinase targets with % probability ranging from 60.0% to 66.7%. The list of putative kinase targets is tabulated in Table 5. The kinase targets that were common among the three compounds **4a**, **4b**, and **4h** were insulin-like growth factor I receptor (IGF1R) and serine/threonine-protein kinase 4 (PLK4) and cyclin-dependent kinase 4 (CDK4).

### 3.5. Drug-Likeness and ADMET Prediction

Lipinski’s rule of five determines the oral bioavailability and drug-likeness of the designed small bioactive molecules [37]. The most active antiproliferative compounds **4a**, **4b**, and **4h** were tested to decide whether or not they obey the Lipinski rule of five, utilizing Swiss ADME predictor [38], as illustrated in Table 6. It can be observed that all of the active compounds comply with Lipinski’s rule of five.

The pharmacokinetics ADMET properties for the most potent compounds were calculated via SwissADME and PreADMET predictors [38,39]. The ADMET data of compounds **4a**, **4b**, and **4h**, shown in Table 7, disclose that they could be easily absorbed from GIT, with a high percentage of human intestinal absorption (HIA%) ranging from 95% to 98%. Their calculated log S values span from −4.52 to −5.16, which proved moderate to good solubilization in GIT fluids before absorption. All tested compounds are predicted to be non-inhibitors of the CYP1A2 enzyme, indicating a low probability of causing drug–drug interaction. In addition, carcinogenicity tests showed that all of them are non-carcinogenic.

However, to develop an anticancer agent with better antiproliferative activity and fewer side effects than the existing chemotype, it is necessary to further investigate other structural derivatives based on this scaffold; further design for structural optimization of the TMP-thiazole series is currently underway and will be reported in due course.

## 4. Conclusions

Among the synthesized pyrimidine-based Trimethoxyphenyl-thiazoles, compounds **4a**, **4b**, and **4h** showed the best growth inhibitory effects over certain cancer cell lines. Among the NCI-selected compounds, **4b** showed the highest GI values of 86.28% and 73.52% against the NSCL cancer cell line HOP-92 and the CNS cancer cell SF-295, respectively. Regarding the MTT-tested compounds, both **4a** and **4h** stood out as the most active members in this series with GI values of 40.87% and 46.14% at 10 μM against HCT-116 colorectal carcinoma and SK-BR-3 breast cancer cell lines, respectively. In addition, Molinspiration and Swiss target predictors showed that the potential molecular targets for compounds **4a**, **4b**, and **4h** are protein kinases. The calculated drug-likeness and ADMET data for compounds **4a**, **4b**, and **4h** underscored their good predicted oral bioavailability and a low probability of carcinogenicity or drug–drug interactions. Further optimization of the presented compounds, especially **4b**, is currently considered to generate potent anticancer chemotypes that may overcome the drawbacks of the existing anticancer chemotherapeutics.

## Figures and Tables

**Table 1:** The % growth inhibition of compounds **4b, 4c, 4k,** and **4n** against the most sensitive cell lines at 10 μM ^a^.

Cancer Type	Cell Line	% Growth Inhibition			
		4b (NSC Code 789748)	4c (NSC Code 789749)	4k (NSC Code 789663)	4n (NSC Code 789664)
**Mean GI%**		**32.20**	21.40	19.40	16.44
**Leukemia**	K-562	38.81	38.60	40.22	8.74
	MOLT-4	42.45	**52.58**	26.07	33.65
	RPMI-8226	45.32	NT	42.85	33.53
	SR	**61.31**	22.65	34.42	23.02
**Non-Small-Cell Lung Cancer**	HOP-92	**86.28**	NT	**64.64**	47.14
	NCI-H522	38.29	**60.42**	**54.63**	45.23
**Colon Cancer**	HCT-116	46.35	39.35	13.70	23.40
	HCT-15	40.25	25.94	13.84	8.04
	HT29	**59.05**	35.34	**62.20**	6.32
**CNS Cancer**	SF-295	**73.52**	15.35	2.09	15.19
	SNB-75	43.32	17.48	46.58	35.64
	U251	41.82	12.14	20.14	22.93
**Melanoma**	LOX IMVI	**57.10**	17.75	16.06	15.60
	SK-MEL-5	26.35	**60.01**	8.82	19.03
	UACC-257	9.91	45.07	10.78	1.86
**Renal Cancer**	786-0	36.71	0.36	45.28	33.67
	A498	**72.38**	24.94	15.91	11.18
**Prostate Cancer**	PC-3	38.70	NT	43.82	42.05
**Breast Cancer**	MDA-MB-231	41.61	9.20	12.02	14.09
	HS 578T	**56.66**	−8.98	21.09	11.50
	T-47D	**70.54**	**62.98**	51.03	**68.39**
	BT-549	49.20	13.39	38.24	43.30

^a^ Data were obtained from the National Cancer Institute (NCI) in vitro disease-oriented human tumor cell line screen. Bold figures refer to GI higher than 50% and bold underlined figures refer to the highest percentage of GI in each compound.

**Table 2:** In vitro anticancer MTT assay of the tested compounds against four human cancer cell lines ^a,b^.

Compound	% Growth Inhibition							
	MCF-7		SK-BR-3		HCT-116		K562	
	100 μM	10 μM	100 μM	10 μM	100 μM	10 μM	100 μM	10 μM
**4a**	**96.03**	35.86	**91.55**	28.22	95.50	40.87	**79.73**	13.57
**4b**	**95.35**	18.76	**90.77**	20.50	94.53	25.43	**92.67**	16.13
**4c**	43.33	36.26	39.64	25.13	32.95	23.66	22.52	16.21
**4d**	43.24	23.88	45.52	18.25	31.54	17.41	9.20	20.13
**4e**	50.70	36.91	47.57	26.63	49.79	32.41	49.57	24.93
**4f**	32.42	6.98	33.38	13.18	28.18	9.97	7.17	18.20
**4g**	40.41	16.68	45.72	19.64	55.49	35.23	28.15	16.06
**4h**	**95.72**	18.88	**90.61**	**46.14**	**94.99**	28.28	**92.86**	10.39
**4i**	**85.90**	11.03	**88.30**	21.83	**92.67**	5.63	**74.12**	8.47
**4j**	**84.12**	5.61	**82.54**	20.04	**92.04**	12.56	**84.50**	11.98
**4k**	**94.96**	4.12	**90.82**	36.57	**94.44**	19.19	**92.84**	7.44
**4l**	**94.82**	9.45	**90.17**	13.23	**94.00**	7.68	**91.67**	17.98
**4m**	**95.43**	16.27	**90.95**	12.52	**94.62**	25.99	**93.00**	6.59
**4n**	**92.04**	1.21	**88.70**	22.61	**93.89**	6.61	**88.28**	7.65
**Sorafenib**	96.06	40.45	93.12	48.87	97.32	48.41	NT	NT

^a^ Compounds were tested in triplicate mode at 100 μM and 10 μM. ^b^ Bold figures refer to GI higher than 70% at 100 μM, and bold underlined figures indicate GI higher than 40% at 10 μM.

**Table 3:** GI_50_ of compounds **4a** and **4h** against L132 normal cell line ^a^.

Compound	GI_50_ (μM)
**4a**	23.52 ± 2.77
**4h**	26.38 ± 1.42

^a^ GI_50_ values were obtained after incubation of the cell line with test compounds for 72 h, and the presented values are the average of at least two independent measurements with standard deviations.

**Table 4:** Predicted Molinspiration bioactivity score for the most active anticancer compounds **4a, 4b**, and **4h**.

Compound	Molinspiration Bioactivity Score					
	GPCR Ligand	Ion Channel Modulator	Kinase Inhibitor	Nuclear Receptor Ligand	Protease Inhibitor	Enzyme Inhibitor
**4a**	0.08	−0.15	0.30	−0.52	−0.30	0.01
**4b**	0.09	−0.14	0.35	−0.46	−0.25	0.05
**4h**	0.10	−0.15	0.38	−0.51	−0.19	0.05

GPCR = G-protein coupled receptor. Score > 0 = active, 0.5–0.0 = moderately active, < −0.5 = inactive.

**Table 5:** List of kinase targets for the most active anticancer compounds **4a**, **4b**, and **4h**, predicted by Swiss TargetPrediction.

Compound	List of Kinase Targets
**4a**	INSR, ALK, IGF1R, CCND1 CDK4, PLK1, PLK4, CDK1, CDK4, FGFR4, PDK1
**4b**	TYRO3, CDK4, CCND1 CDK4, PLK4, MAPK1, IGF1R
**4h**	IGF1R, CDK1, CDK4, ALK, AKT1, PLK1, PLK4, MET, TYRO3, INSR

**Table 6:** Calculated Lipinski’s rule of five parameters for the most active anticancer compounds **4a, 4b,** and **4h**.

Compound	Parameter						*n*Vs(g)
	Log P(a)	TPSA(b)	MW(c)	*n*HBA(d)	*n*HBD(e)	*n*RB(f)	
**4a**	4.06	113.11	470.59	7	1	8	0
**4b**	3.77	133.34	486.59	8	2	9	0
**4h**	4.32	125.14	499.63	8	2	10	0

(a) Calculated lipophilicity. (b) Total polar surface area. (c) Molecular weight. (d) Number of hydrogen bond acceptors. (e) Number of hydrogen bond doners. (f) Number of rotatable bonds. (g) Number of violations from Lipinski’s rule of five.

**Table 7:** Predicted ADMET data for the most active anticancer compounds **4a, 4b,** and **4h**.

Compound	HIA%	Log S	CYP1A2 Inhibition	Carcinogenicity
**4a**	98.05	−5.16	Non-inhibitor	Non-carcinogenic
**4b**	95.68	−4.52	Non-inhibitor	Non-carcinogenic
**4h**	96.11	−4.90	Non-inhibitor	Non-carcinogenic

## Data Availability

Not applicable.

## Supplementary Materials

The following supporting information can be downloaded at: https://www.mdpi.com/article/10.3390/medicina59061076/s1, Figure S1, ^1^H NMR and ^13^C NMR spectra; Figure S2, HRMS charts; Figure S3, HPLC chromatograms; Figure S4, NCI-60 cell line screening results.
