# Supplementary material for: Design and Synthesis of New 4-(3,4,5-Trimethoxyphenyl)Thiazole–Pyrimidine Derivatives as Potential Antiproliferative Agents
Source: Medicina (Kaunas). 2023 Jun 2;59(6):1076. doi: 10.3390/medicina59061076 (PMC10303439; doi:10.3390/medicina59061076)
Supplement: Supplementary file 1 [file medicina-59-01076-s001.zip › medicina-2413630-supplementary.pdf]

Supporting Information

Design and Synthesis of New 4-(3,4,5-Trimethoxyphenyl) Thiazole–Pyrimidine Derivatives as Potential Antiproliferative Agents

Ashraf K. El-Damasy [1,2,†], Heewon Jin [1,†], Mohamed A. Sabry [2], Hyun Ji Kim [1], Mohammed M. Alanazi [3], Seon Hee Seo [4], Eun-Kyoung Bang [1] and Gyochang Keum [1,5,*]

1. Center for Brain Technology, Brain Science Institute, Korea Institute of Science and Technology (KIST), Seoul 02792, Republic of Korea; ph_karem2000@mans.edu.eg (A.K.E.-D.); hwjin37@snu.ac.kr (H.J.); hjkim926@kist.re.kr (H.J.K.); eunkbang@kist.re.kr (E.-K.B.)
2. Department of Medicinal Chemistry, Faculty of Pharmacy, Mansoura University, Mansoura 35516, Egypt; midoegy1992@hotmail.com
3. Department of Pharmaceutical Chemistry, College of Pharmacy, King Saud University, Riyadh 11451, Saudi Arabia; mmalanazi@ksu.edu.sa
4. Center for Brain Disorders, Brain Science Institute, Korea Institute of Science and Technology (KIST), Seoul 02792, Republic of Korea; shseo@kist.re.kr
5. Division of Bio-Medical Science & Technology, KIST School, Korea University of Science and Technology (UST), Seoul 02792, Republic of Korea

* Correspondence: gkeum@kist.re.kr

† These authors contributed equally to the work.

Contents **Pages**

Figure S1. ^{1}H NMR and ^{13}C NMR spectra. 2–18
Figure S2. HRMS charts. 19–33
Figure S3. HPLC chromatograms. 34–37
Figure S4. NCI-60 cell line screening results. 38–41

Figure S1. ^{1}H NMR and ^{13}C NMR spectra.

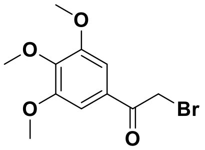

1

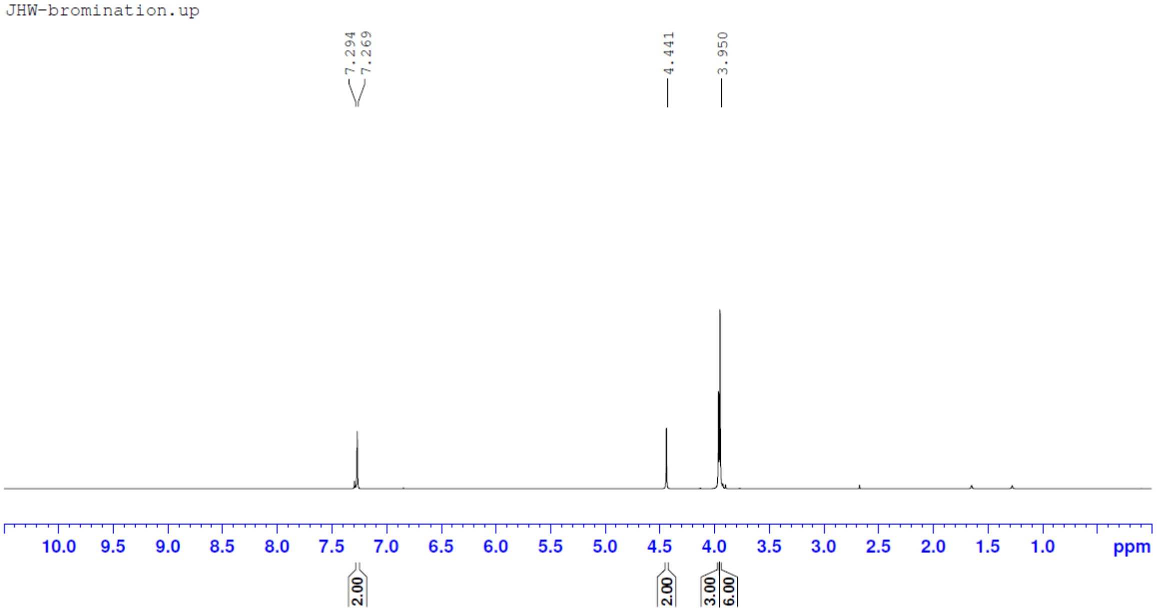

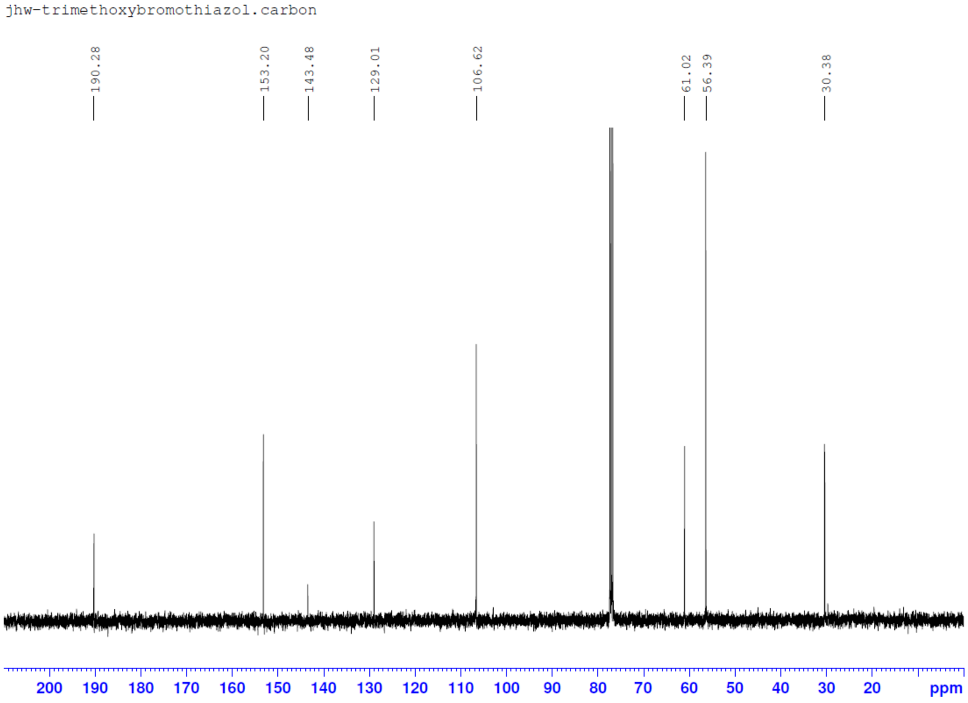

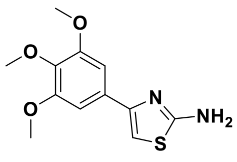

2

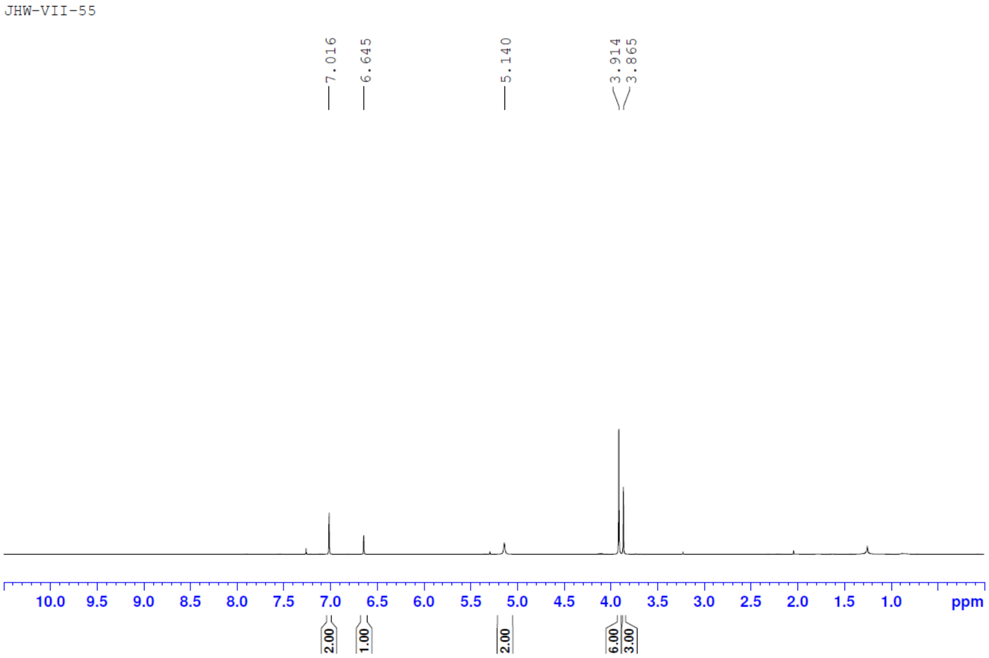
JHW-VII-55
7.016
6.645
5.140
3.914
3.865
10.0 9.5 9.0 8.5 8.0 7.5 7.0 6.5 6.0 5.5 5.0 4.5 4.0 3.5 3.0 2.5 2.0 1.5 1.0 ppm
2.00
1.00
2.00
6.00
3.00

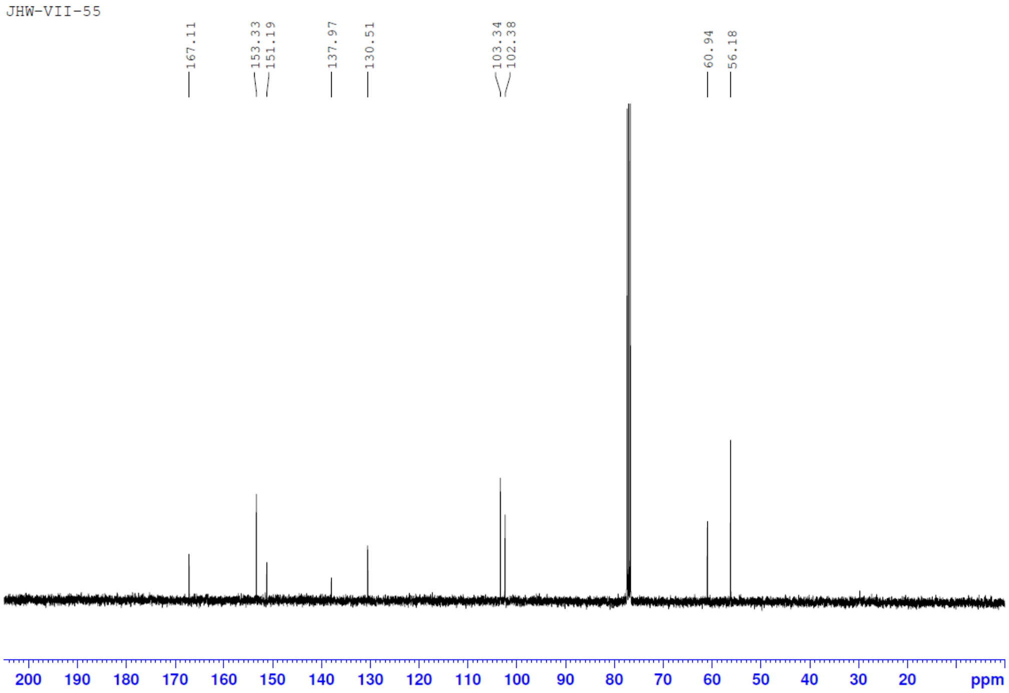
JHW-VII-55
167.11
153.33
151.19
137.97
130.51
103.34
102.38
60.94
56.18
200 190 180 170 160 150 140 130 120 110 100 90 80 70 60 50 40 30 20 ppm

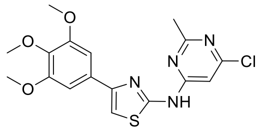
O
O
O
N
S
NH
N
N
Cl

3

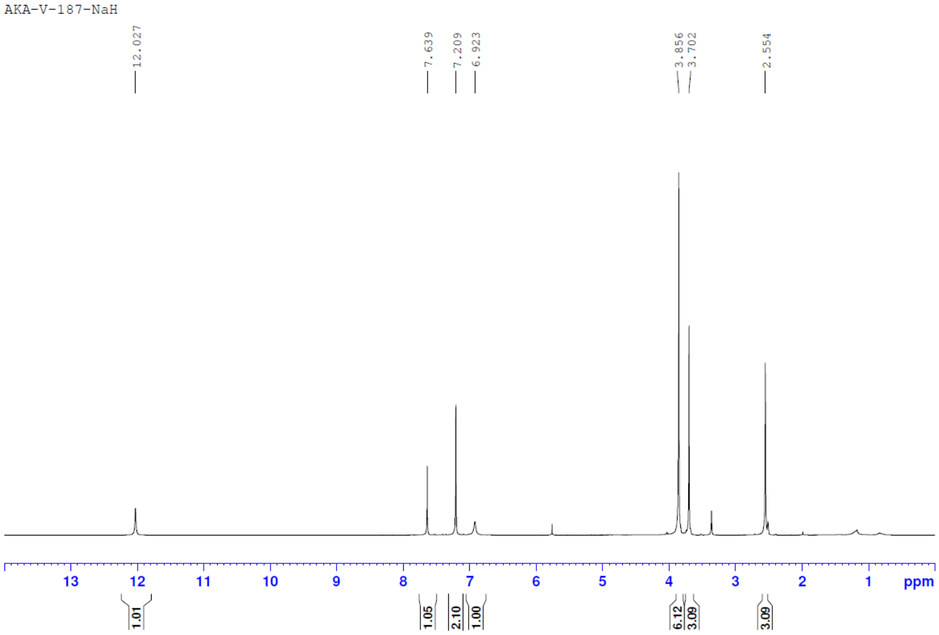
AKA-V-187-NaH
12.027
7.639
7.209
6.923
3.856
3.702
2.554
13
12
11
10
9
8
7
6
5
4
3
2
1
ppm
1.01
1.05
2.10
1.00
6.12
3.09
3.09

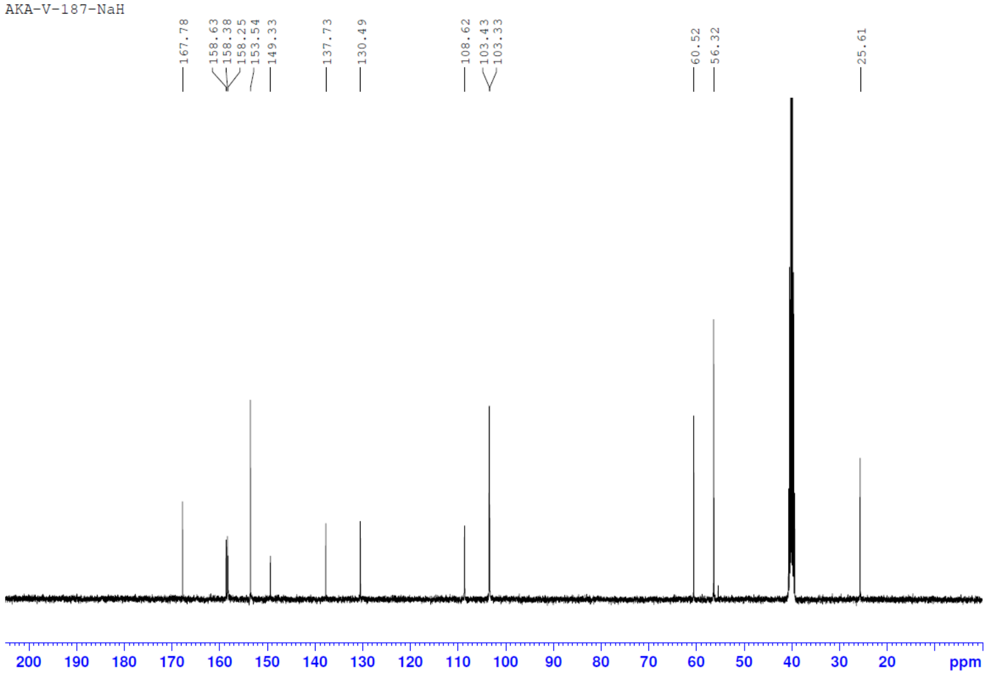
AKA-V-187-NaH
167.78
158.63
158.38
158.25
153.54
149.33
137.73
130.49
108.62
103.43
103.33
60.52
56.32
25.61
200
190
180
170
160
150
140
130
120
110
100
90
80
70
60
50
40
30
20
ppm

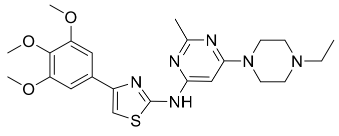
O
O
O
N
S
NH
N
N
N
N

4a

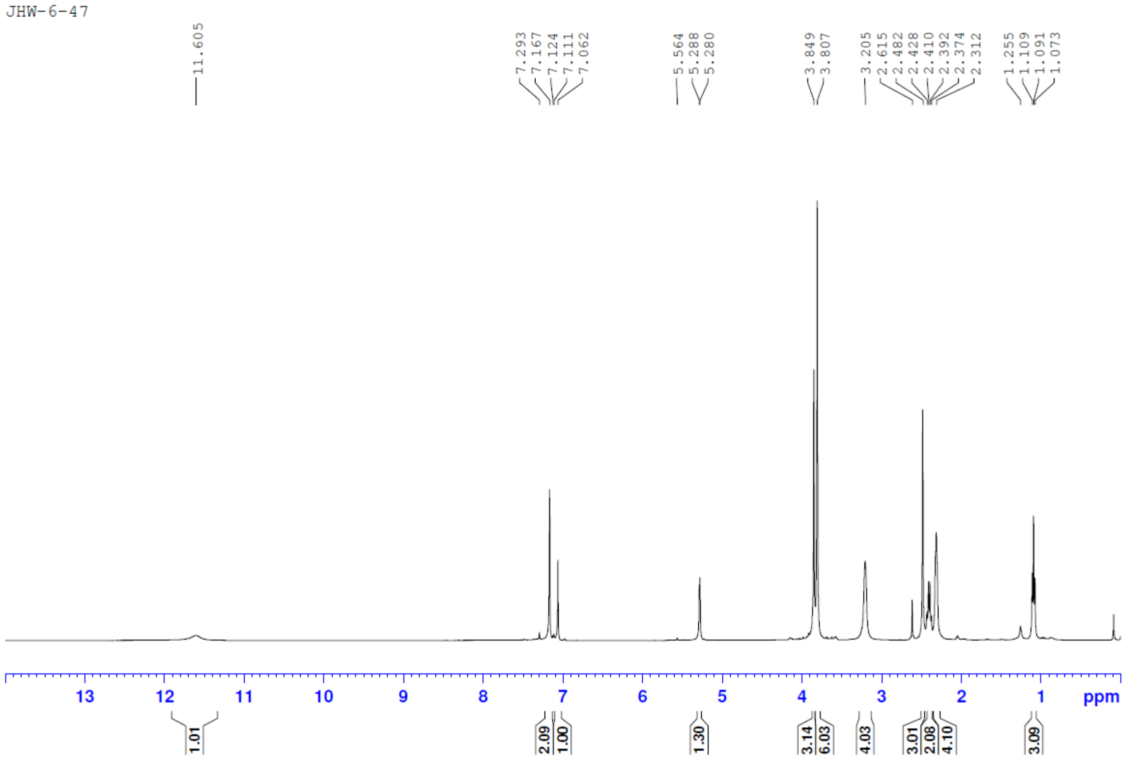
JHW-6-47
11.605
7.293
7.167
7.124
7.111
7.062
5.564
5.288
5.280
3.849
3.807
3.205
2.615
2.482
2.428
2.410
2.392
2.374
2.312
1.255
1.109
1.091
1.073
13
12
11
10
9
8
7
6
5
4
3
2
1
ppm
1.01
2.09
1.00
1.30
3.14
6.03
4.03
3.01
2.08
4.10
3.09

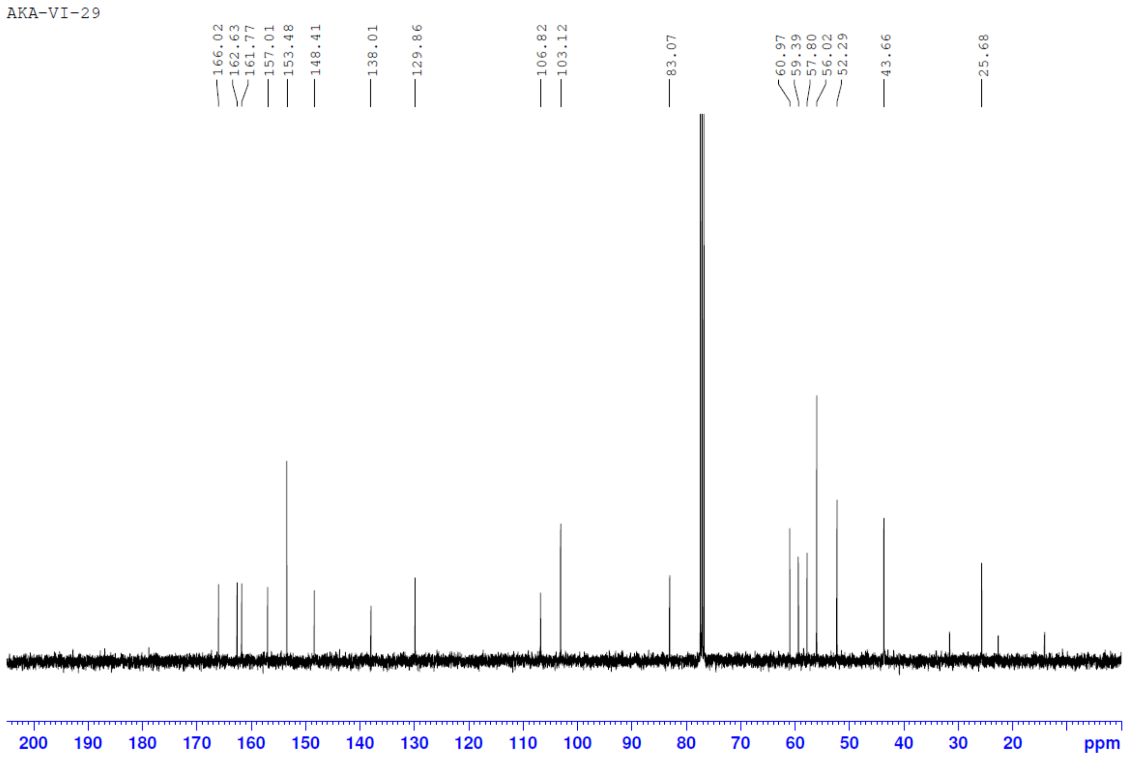
AKA-VI-29
166.02
162.63
161.77
157.01
153.48
148.41
138.01
129.86
106.82
103.12
83.07
60.97
59.39
57.80
56.02
52.29
43.66
25.68
200
190
180
170
160
150
140
130
120
110
100
90
80
70
60
50
40
30
20
ppm

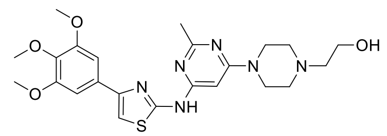

4b

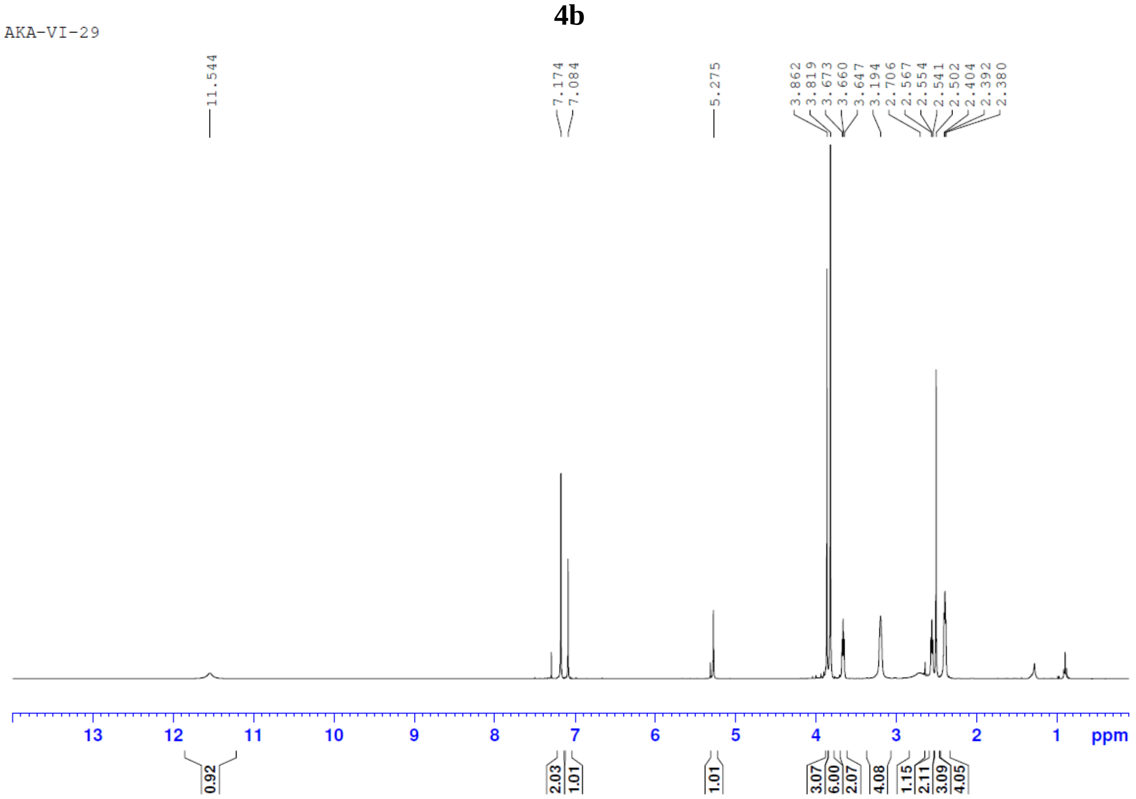
AKA-VI-29
11.544
7.174
7.084
5.275
3.862
3.819
3.673
3.660
3.647
3.194
2.706
2.567
2.554
2.541
2.502
2.404
2.392
2.380
13 12 11 10 9 8 7 6 5 4 3 2 1 ppm
0.92
2.03
1.01
1.01
3.07
6.00
2.07
4.08
1.15
2.11
3.09
4.05

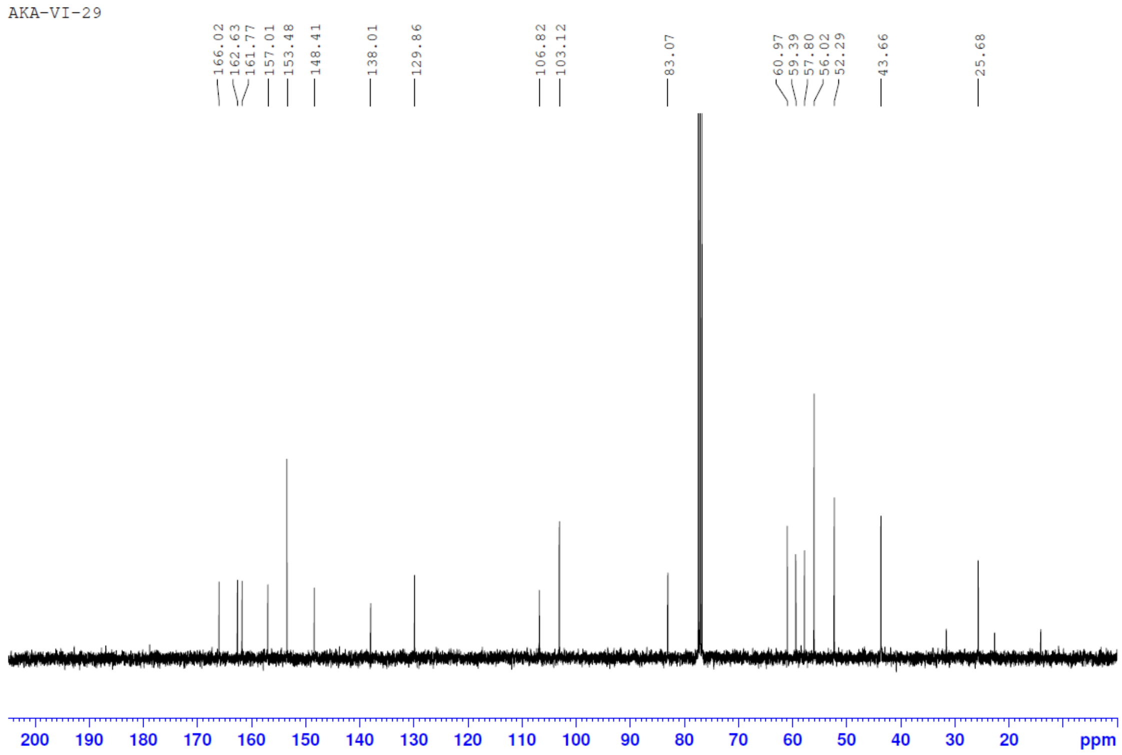
AKA-VI-29
166.02
162.63
161.77
157.01
153.48
148.41
138.01
129.86
106.82
103.12
83.07
60.97
59.39
57.80
56.02
52.29
43.66
25.68
200 190 180 170 160 150 140 130 120 110 100 90 80 70 60 50 40 30 20 ppm

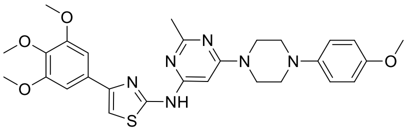

4c

AKA-VI-27

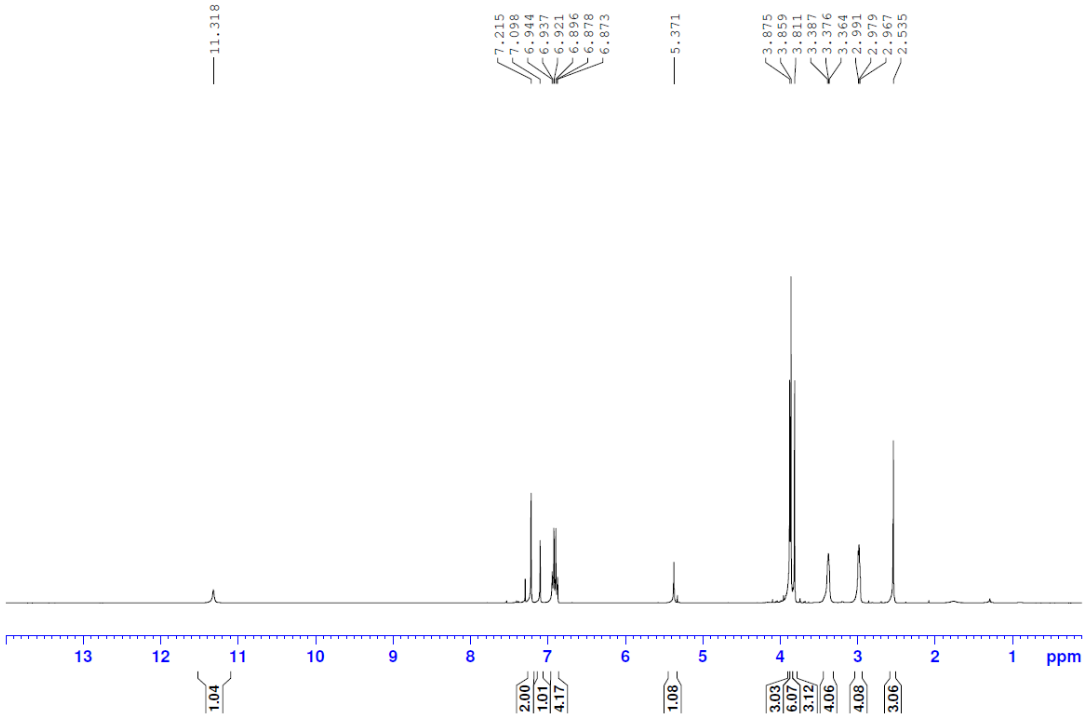

AKA-VI-27

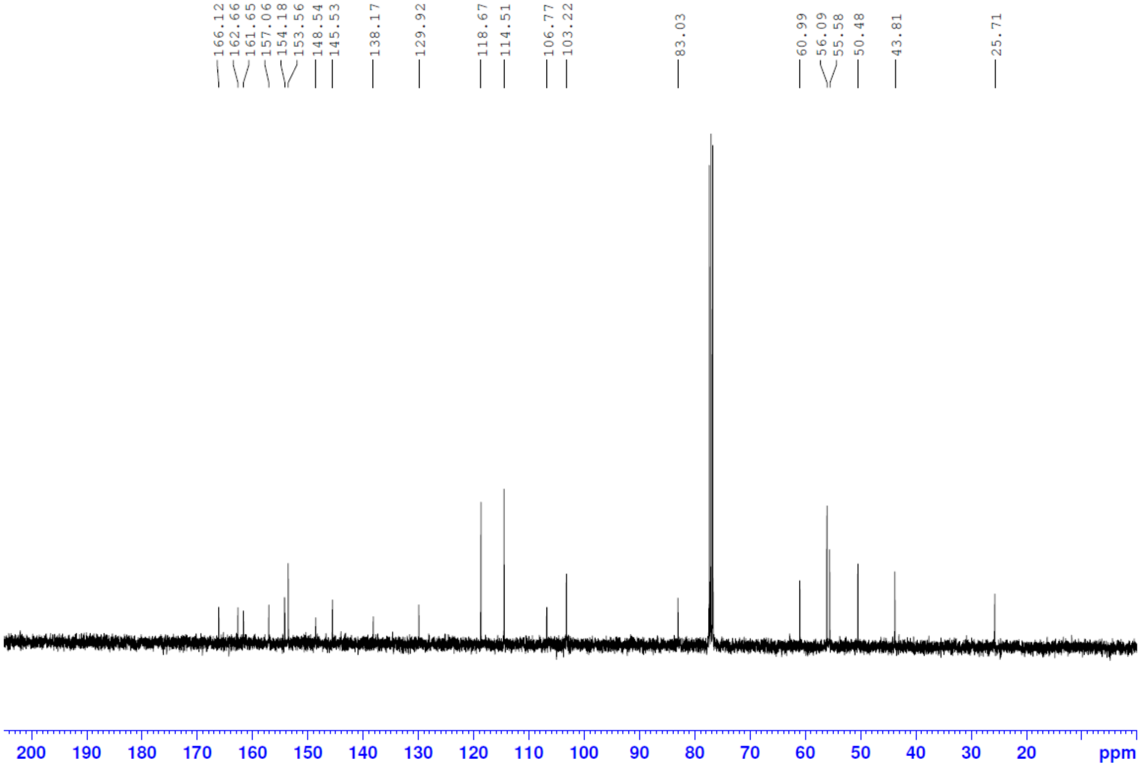

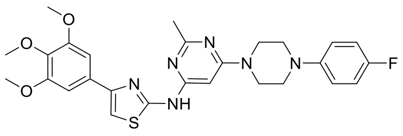
O
O
O
N
S
NH
N
N
N
N
F

4d

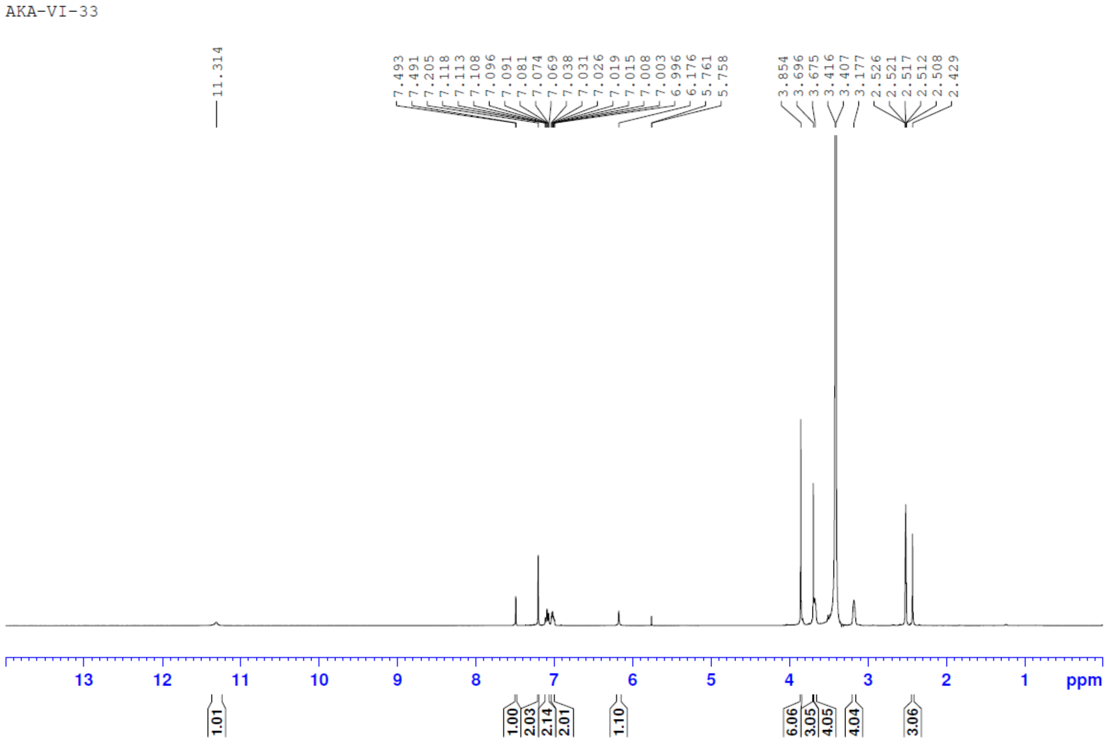
AKA-VI-33
11.314
7.493
7.491
7.205
7.118
7.113
7.108
7.096
7.091
7.081
7.074
7.069
7.038
7.031
7.026
7.019
7.015
7.008
7.003
6.996
6.176
5.761
5.758
3.854
3.696
3.675
3.416
3.407
3.177
2.526
2.521
2.517
2.512
2.508
2.429
13 12 11 10 9 8 7 6 5 4 3 2 1 ppm
1.01
1.00
2.03
2.14
2.01
1.10
6.06
3.05
4.05
4.04
3.06

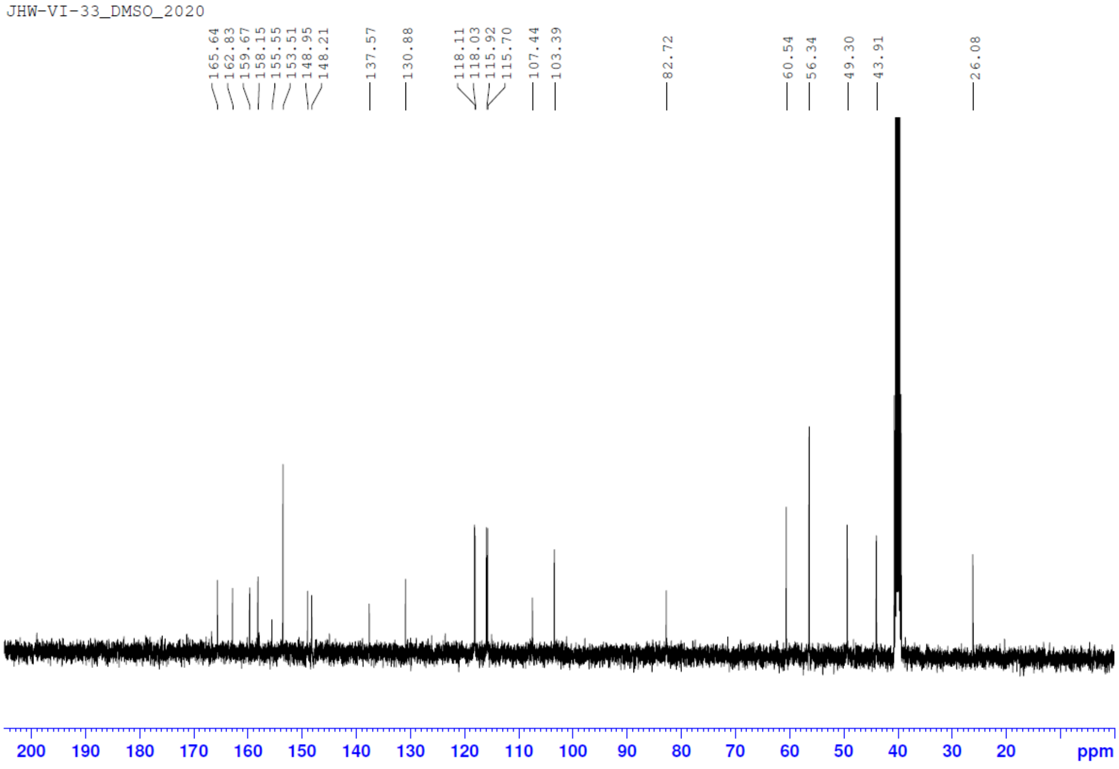
JHW-VI-33_DMSO_2020
165.64
162.83
159.67
158.15
155.55
153.51
148.95
148.21
137.57
130.88
118.11
118.03
115.92
115.70
107.44
103.39
82.72
60.54
56.34
49.30
43.91
26.08
200 190 180 170 160 150 140 130 120 110 100 90 80 70 60 50 40 30 20 ppm

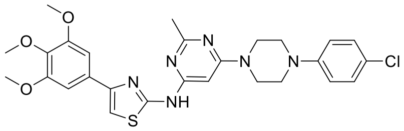
O
O
O
N
N
N
N
N
S
NH
Cl

4e

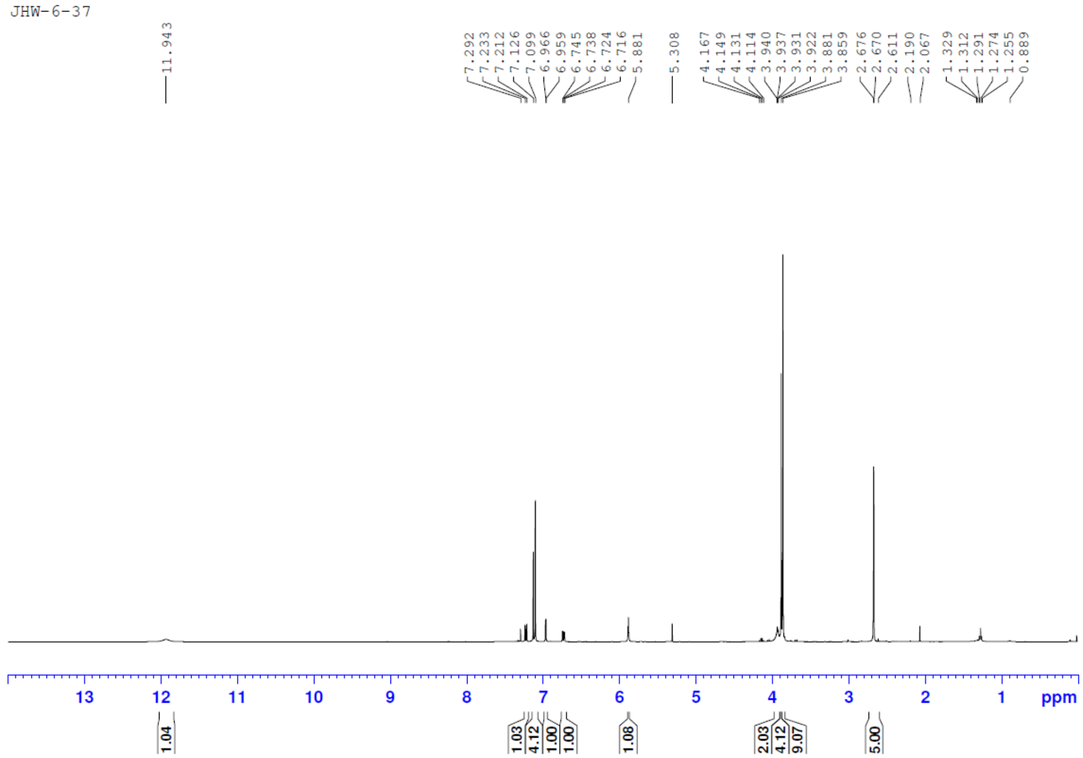
JHW-6-37
11.943
7.292 7.233 7.212 7.126 7.099 6.966 6.959 6.745 6.738 6.724 6.716 5.881
5.308
4.167 4.149 4.131 4.114 3.940 3.937 3.931 3.922 3.881 3.859 2.676 2.670 2.611 2.190 2.067
1.329 1.312 1.291 1.274 1.255 0.889
13 12 11 10 9 8 7 6 5 4 3 2 1 ppm
1.04
1.03 4.12 1.00 1.00
1.08
2.03 4.12 9.07
5.00

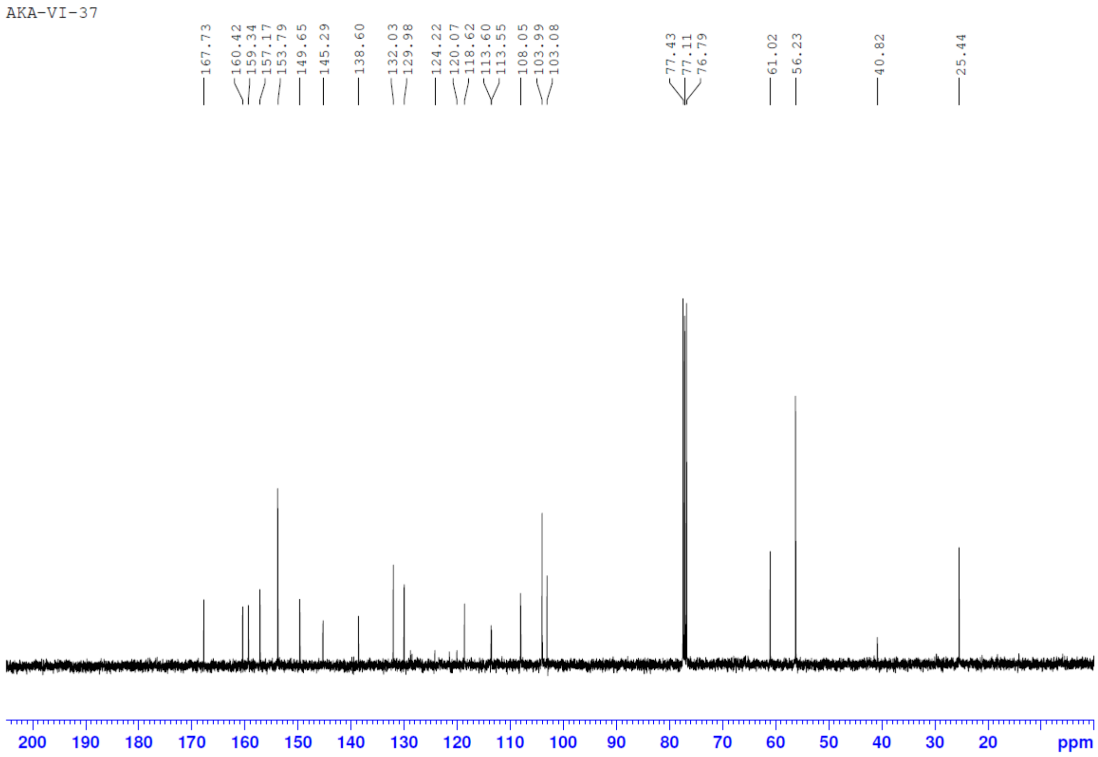
AKA-VI-37
167.73
160.42 159.34 157.17 153.79 149.65 145.29
138.60
132.03 129.98
124.22 120.07 118.62 113.60 113.55
108.05 103.99 103.08
77.43 77.11 76.79
61.02 56.23
40.82
25.44
200 190 180 170 160 150 140 130 120 110 100 90 80 70 60 50 40 30 20 ppm

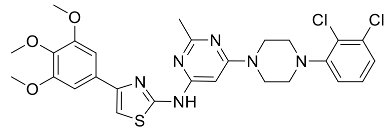
O
O
O
N
S
NH
N
N
N
N
Cl
Cl

4f

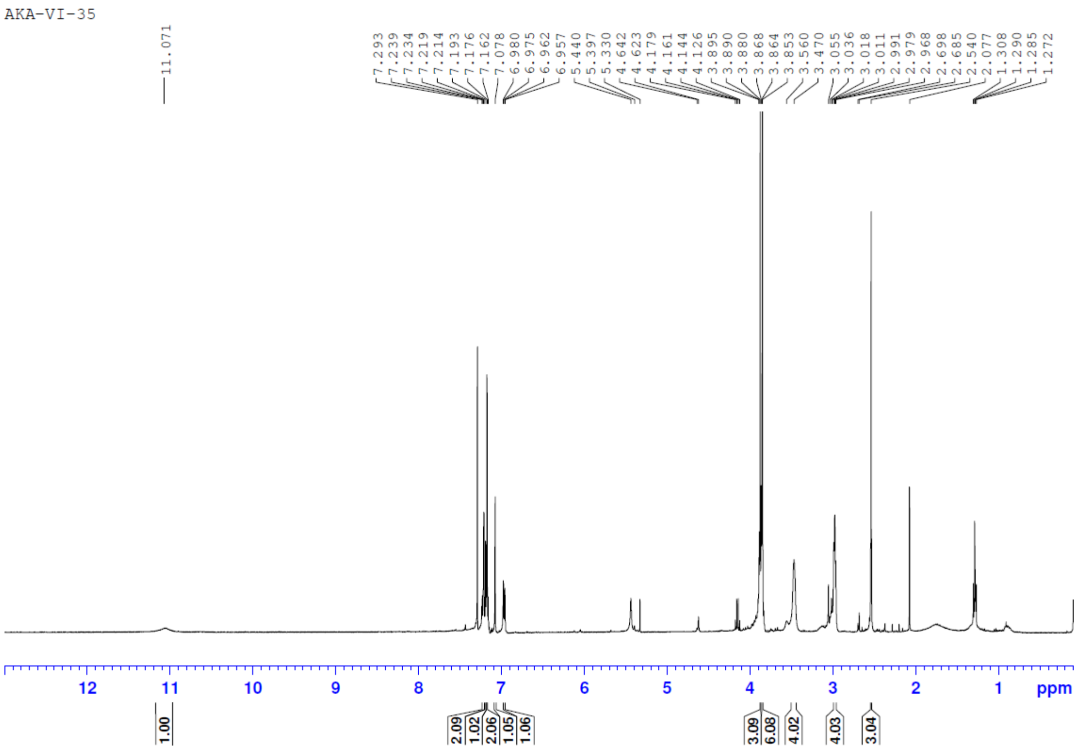
AKA-VI-35
11.071
7.293
7.239
7.234
7.219
7.214
7.193
7.176
7.162
7.078
6.980
6.975
6.962
6.957
5.440
5.397
5.330
4.642
4.623
4.179
4.161
4.144
4.126
3.895
3.890
3.880
3.868
3.864
3.853
3.560
3.470
3.055
3.036
3.018
3.011
2.991
2.979
2.968
2.698
2.685
2.540
2.077
1.308
1.290
1.285
1.272
12
11
10
9
8
7
6
5
4
3
2
1
ppm
1.00
2.09
1.02
2.06
1.05
1.06
3.09
6.08
4.02
4.03
3.04

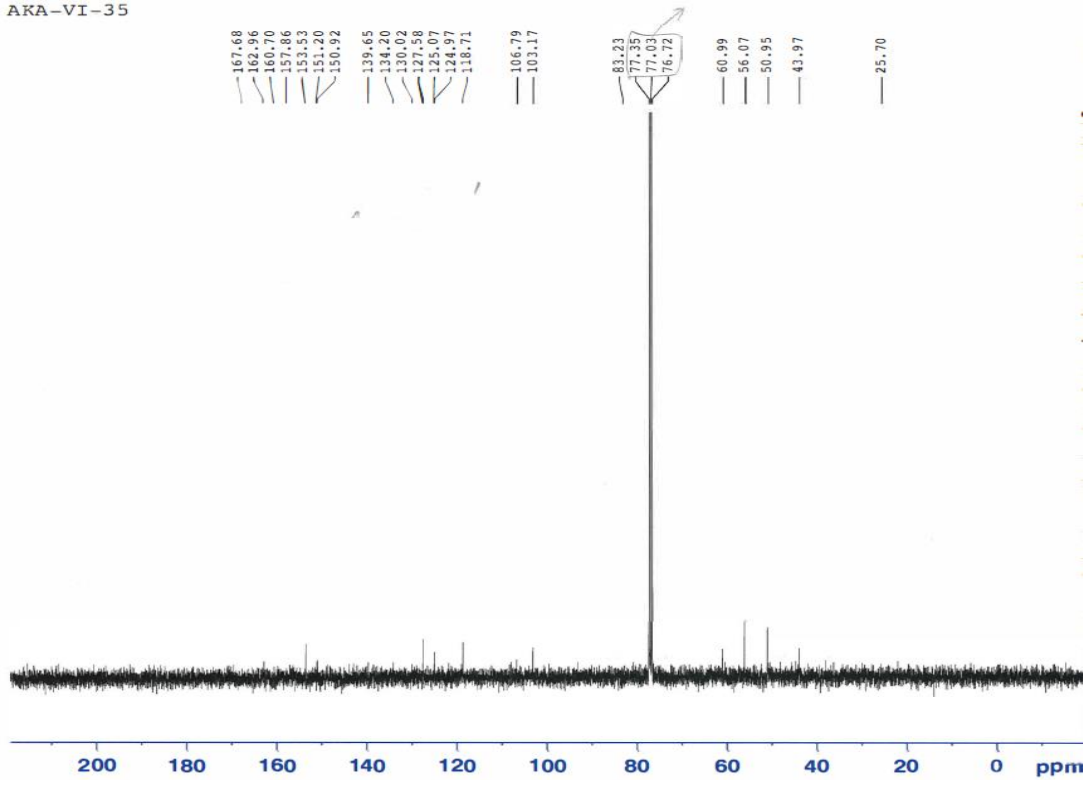
AKA-VI-35
167.68
162.96
160.70
157.86
153.53
151.20
150.92
139.65
134.20
130.02
127.58
125.07
124.97
118.71
106.79
103.17
83.23
77.35
77.03
76.72
60.99
56.07
50.95
43.97
25.70
200
180
160
140
120
100
80
60
40
20
0
ppm

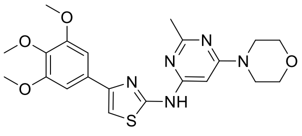
O
O
O
N
S
NH
N
N
N
O

4g

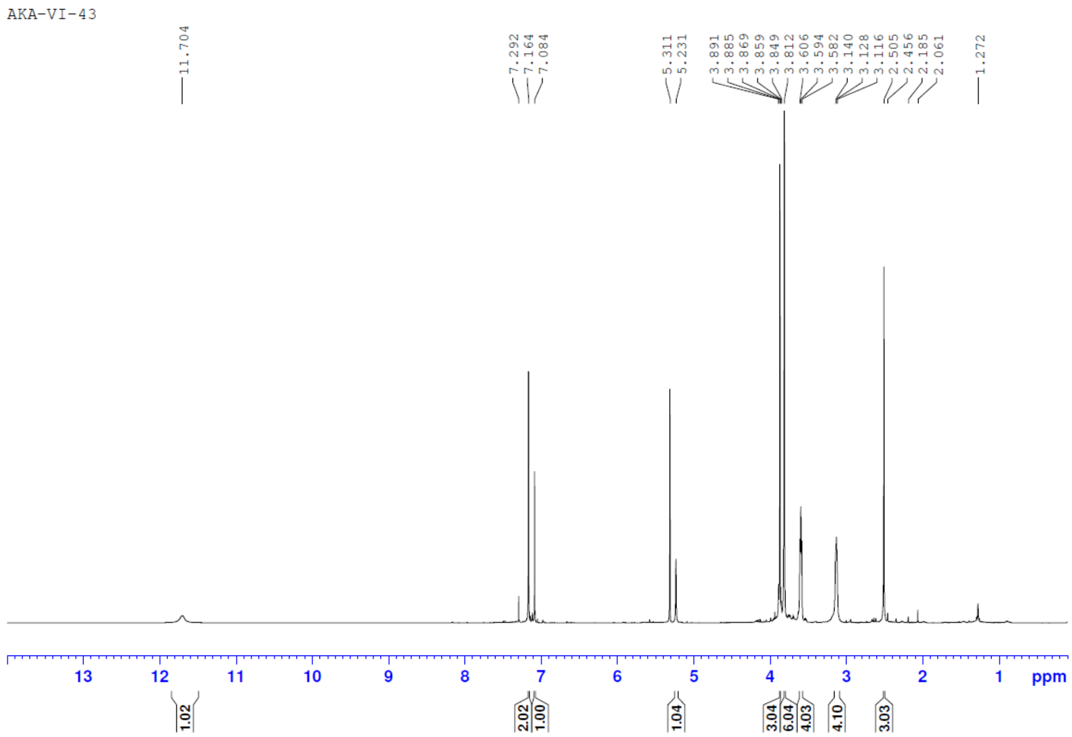
AKA-VI-43
11.704
7.292
7.164
7.084
5.311
5.231
3.891
3.885
3.869
3.859
3.849
3.812
3.606
3.594
3.582
3.140
3.128
3.116
2.505
2.456
2.185
2.061
1.272
13 12 11 10 9 8 7 6 5 4 3 2 1 ppm
1.02
2.02
1.00
1.04
3.04
6.04
4.03
4.10
3.03

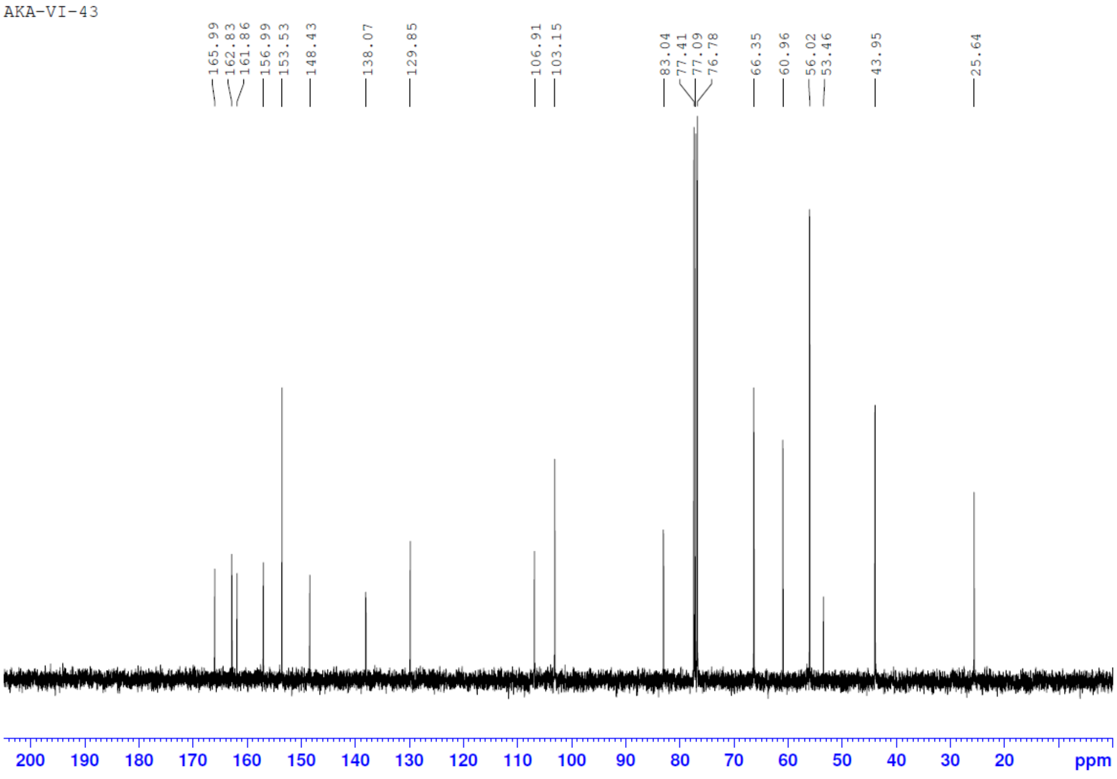
AKA-VI-43
165.99
162.83
161.86
156.99
153.53
148.43
138.07
129.85
106.91
103.15
83.04
77.41
77.09
76.78
66.35
60.96
56.02
53.46
43.95
25.64
200 190 180 170 160 150 140 130 120 110 100 90 80 70 60 50 40 30 20 ppm

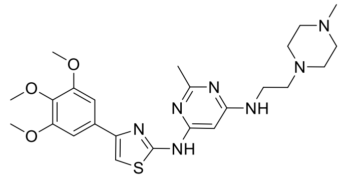
O
O
O
N
S
NH
N
N
NH
N
N

4h

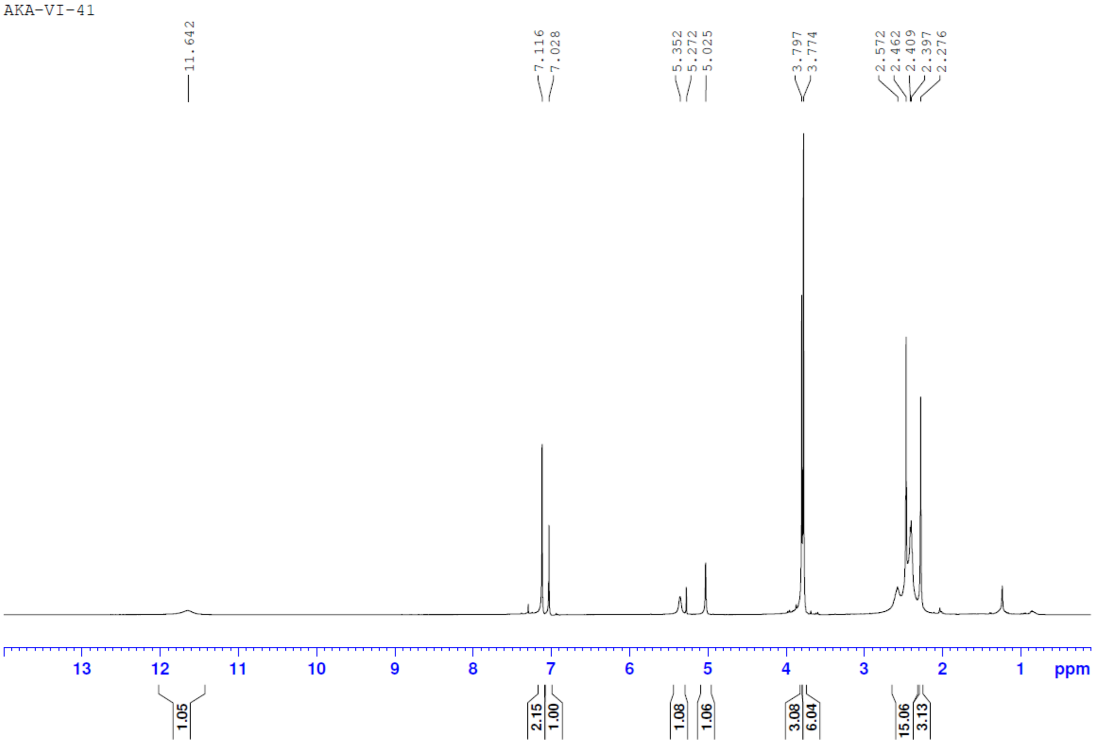
AKA-VI-41
11.642
7.116
7.028
5.352
5.272
5.025
3.797
3.774
2.572
2.462
2.409
2.397
2.276
13 12 11 10 9 8 7 6 5 4 3 2 1 ppm
1.05
2.15
1.00
1.08
1.06
3.08
6.04
15.06
3.13

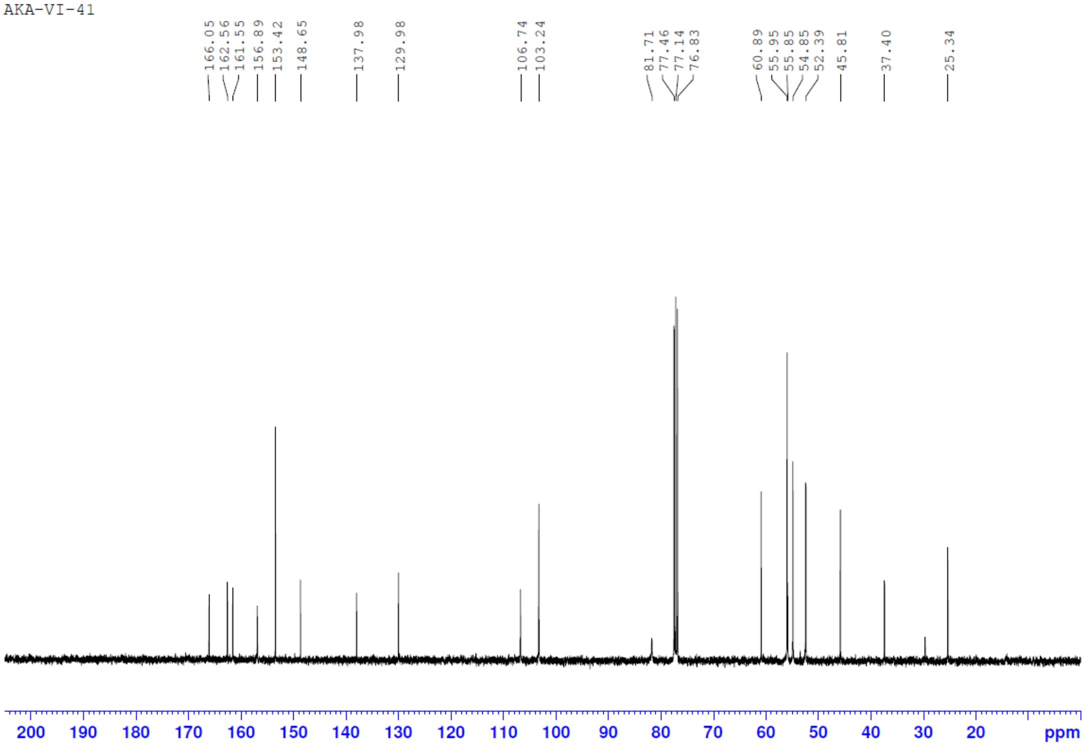
AKA-VI-41
166.05
162.56
161.55
156.89
153.42
148.65
137.98
129.98
106.74
103.24
81.71
77.46
77.14
76.83
60.89
55.95
55.85
54.85
52.39
45.81
37.40
25.34
200 190 180 170 160 150 140 130 120 110 100 90 80 70 60 50 40 30 20 ppm

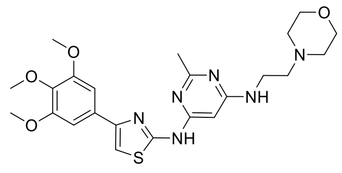
O
O
O
N
S
NH
N
N
NH
N
O

4i

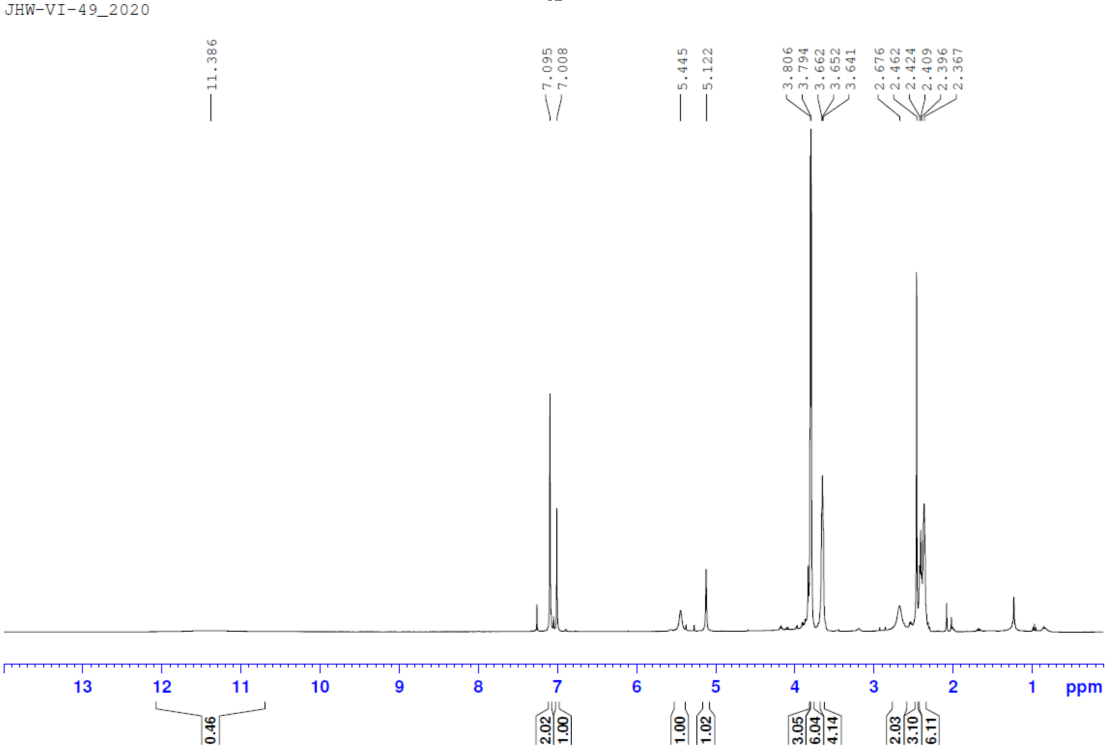
JHW-VI-49_2020
11.386
7.095
7.008
5.445
5.122
3.806
3.794
3.662
3.652
3.641
2.676
2.462
2.424
2.409
2.396
2.367
13 12 11 10 9 8 7 6 5 4 3 2 1 ppm
0.46
2.02
1.00
1.00
1.02
3.05
6.04
4.14
2.03
3.10
6.11

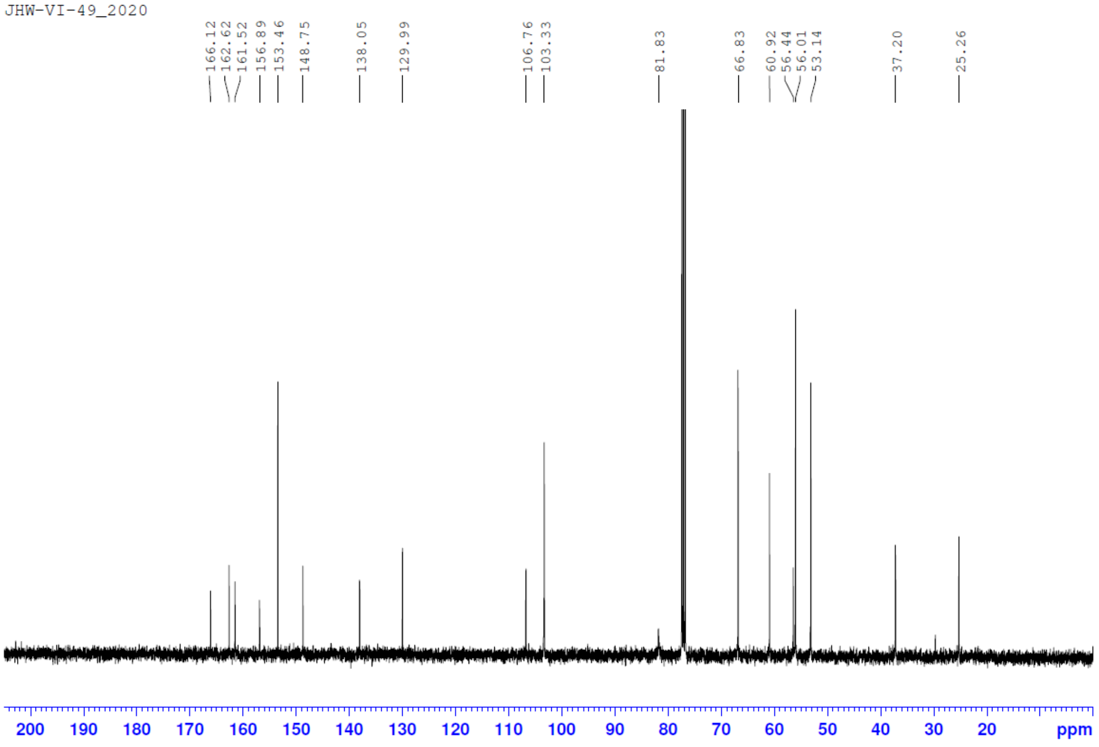
JHW-VI-49_2020
166.12
162.62
161.52
156.89
153.46
148.75
138.05
129.99
106.76
103.33
81.83
66.83
60.92
56.44
56.01
53.14
37.20
25.26
200 190 180 170 160 150 140 130 120 110 100 90 80 70 60 50 40 30 20 ppm

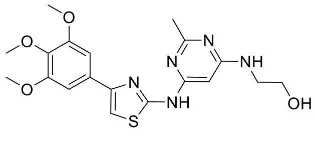

4j

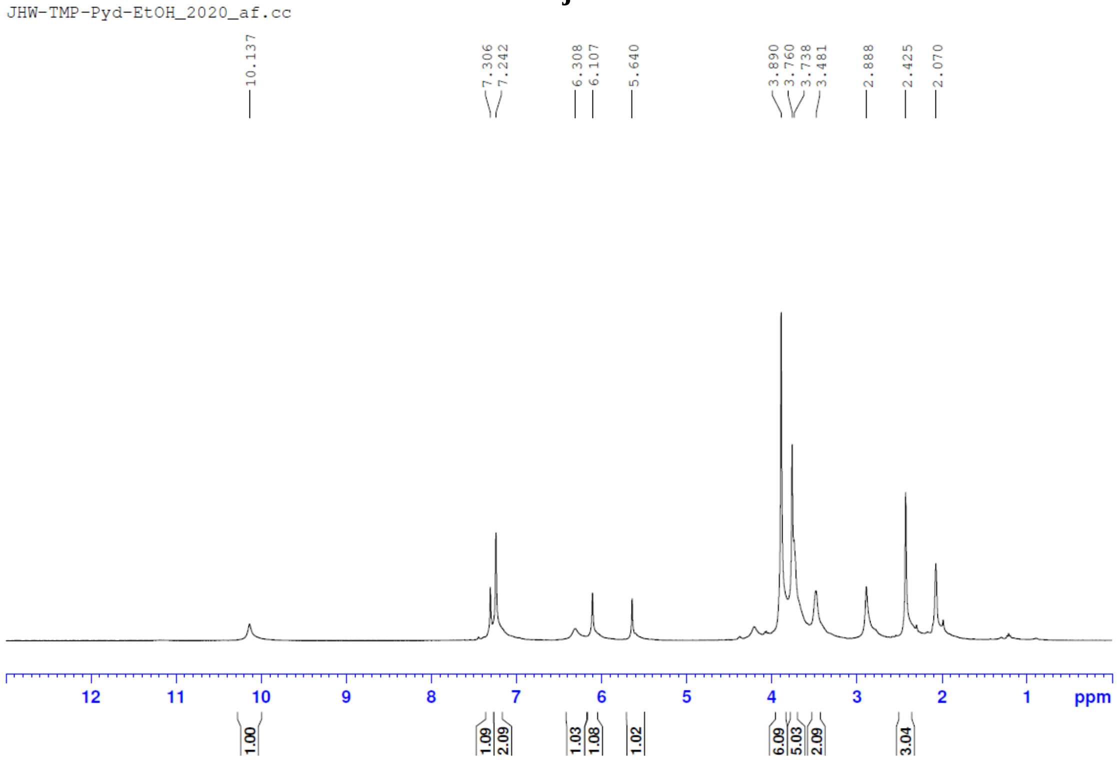
JHW-TMP-Pyd-EtOH_2020_af.cc
10.137
7.306
7.242
6.308
6.107
5.640
3.890
3.760
3.738
3.481
2.888
2.425
2.070
12 11 10 9 8 7 6 5 4 3 2 1 ppm
1.00
1.09
2.09
1.03
1.08
1.02
6.09
5.03
2.09
3.04

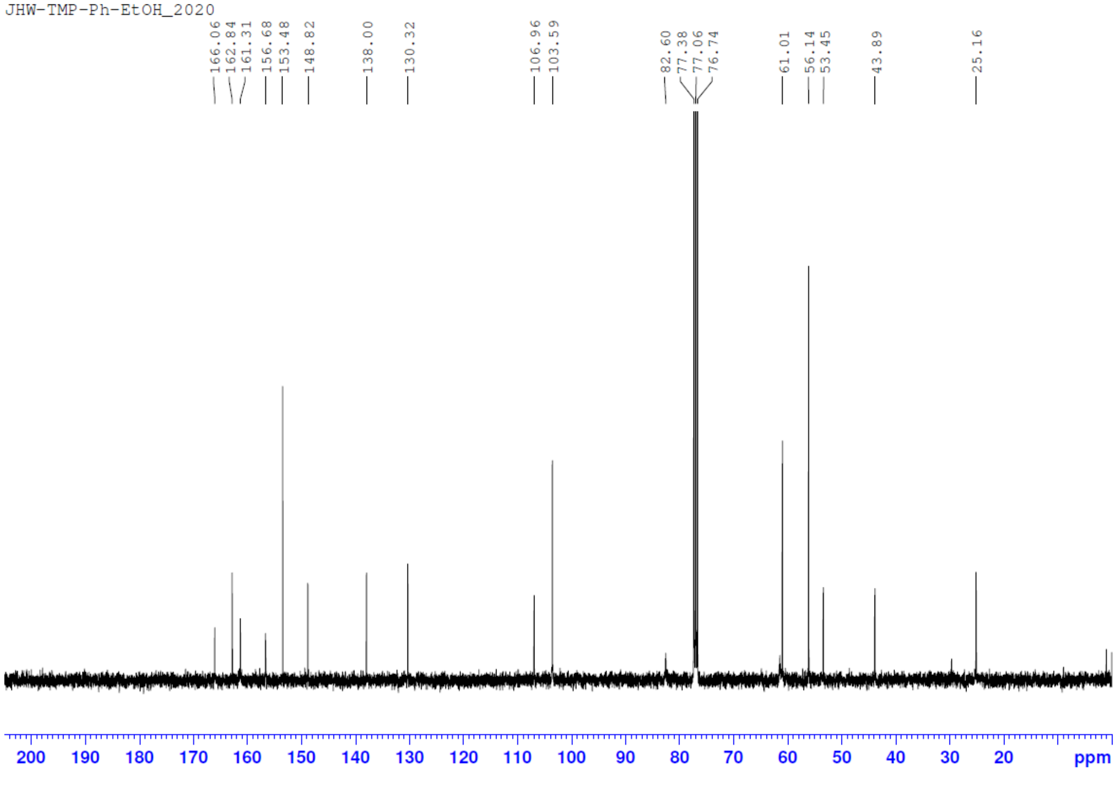
JHW-TMP-Ph-EtOH_2020
166.06
162.84
161.31
156.68
153.48
148.82
138.00
130.32
106.96
103.59
82.60
77.38
77.06
76.74
61.01
56.14
53.45
43.89
25.16
200 190 180 170 160 150 140 130 120 110 100 90 80 70 60 50 40 30 20 ppm

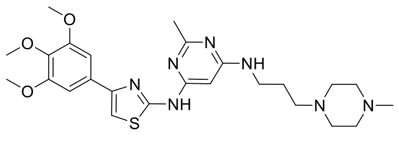
O
O
O
N
S
NH
N
N
NH
N
N

4k

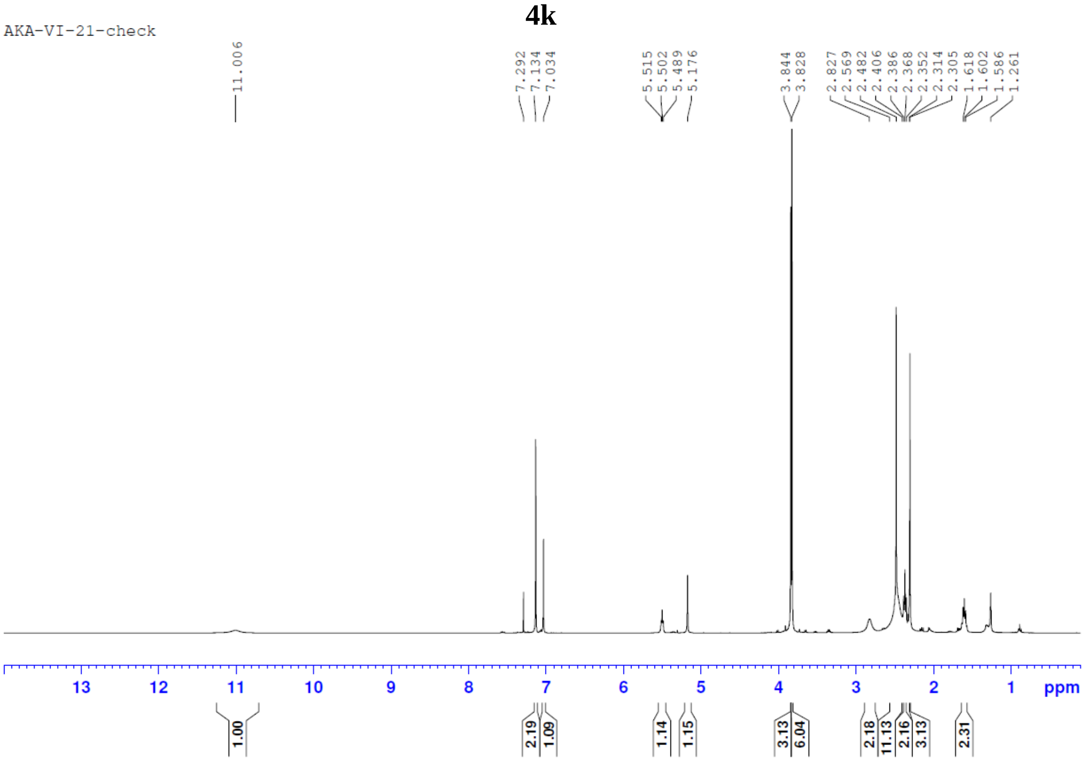
AKA-VI-21-check
11.006
7.292
7.134
7.034
5.515
5.502
5.489
5.176
3.844
3.828
2.827
2.569
2.482
2.406
2.386
2.368
2.352
2.314
2.305
1.618
1.602
1.586
1.261
13
12
11
10
9
8
7
6
5
4
3
2
1
ppm
1.00
2.19
1.09
1.14
1.15
3.13
6.04
2.18
11.13
2.16
3.13
2.31

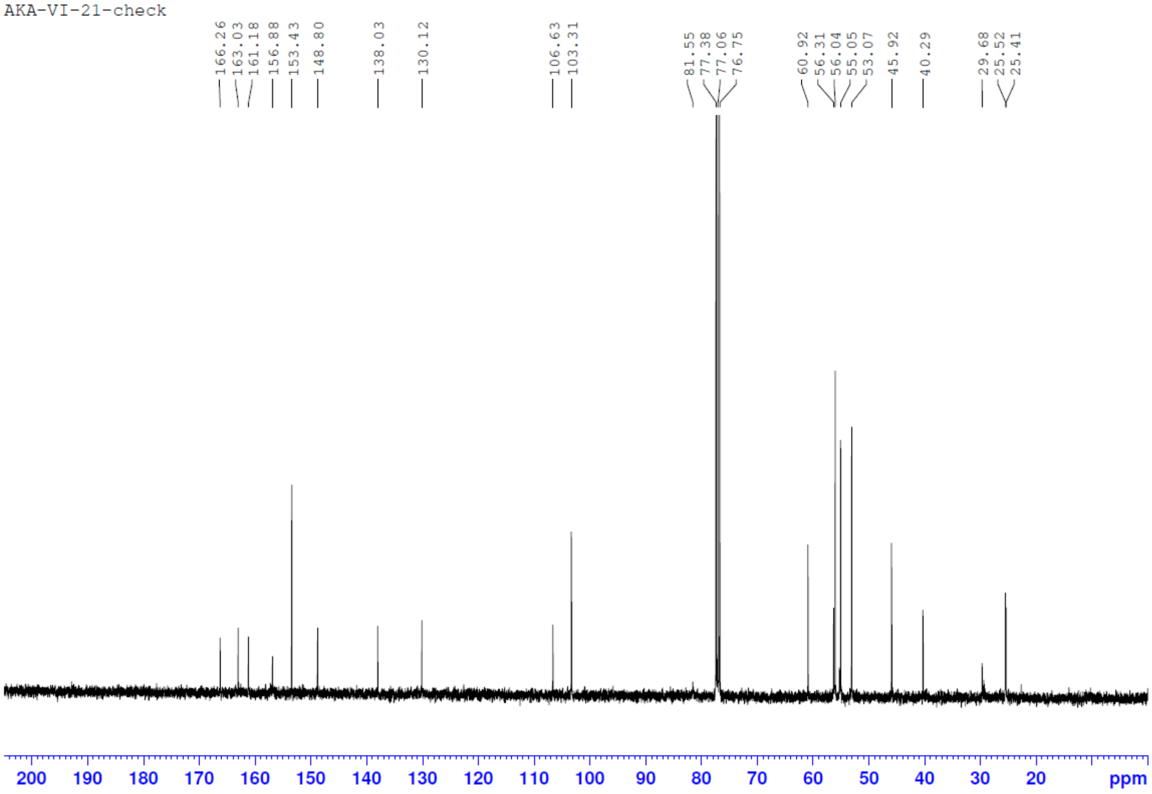
AKA-VI-21-check
166.26
163.03
161.18
156.88
153.43
148.80
138.03
130.12
106.63
103.31
81.55
77.38
77.06
76.75
60.92
56.31
56.04
55.05
53.07
45.92
40.29
29.68
25.52
25.41
200
190
180
170
160
150
140
130
120
110
100
90
80
70
60
50
40
30
20
ppm

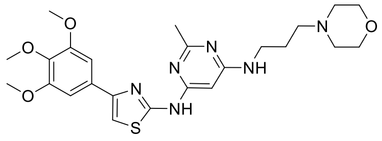

4l

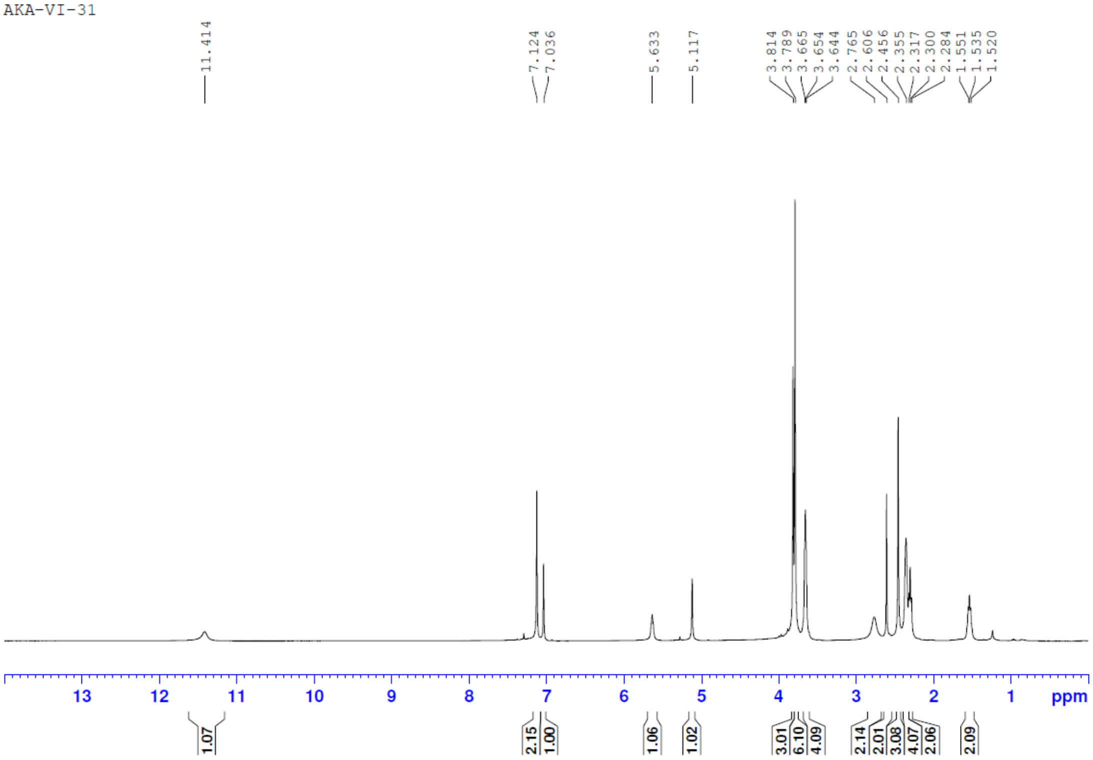
AKA-VI-31
11.414
7.124
7.036
5.633
5.117
3.814
3.789
3.665
3.654
3.644
2.765
2.606
2.456
2.355
2.317
2.300
2.284
1.551
1.535
1.520
13
12
11
10
9
8
7
6
5
4
3
2
1
ppm
1.07
2.15
1.00
1.06
1.02
3.01
6.10
4.09
2.14
2.01
3.08
4.07
2.06
2.09

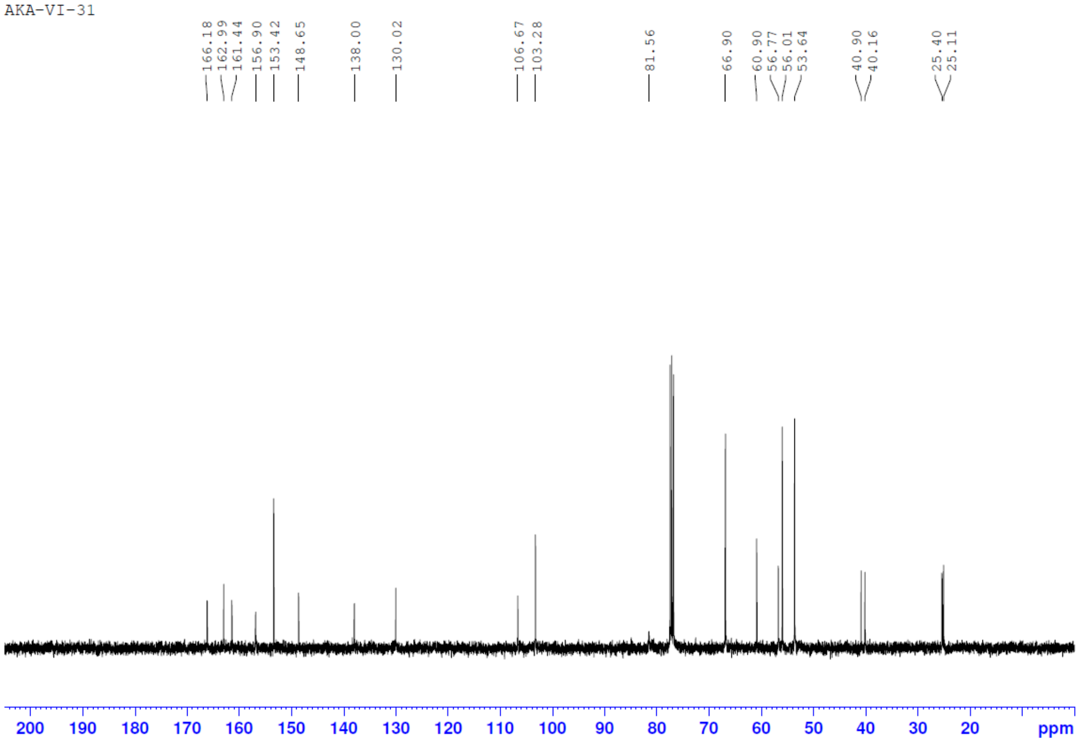
AKA-VI-31
166.18
162.99
161.44
156.90
153.42
148.65
138.00
130.02
106.67
103.28
81.56
66.90
60.90
56.77
56.01
53.64
40.90
40.16
25.40
25.11
200
190
180
170
160
150
140
130
120
110
100
90
80
70
60
50
40
30
20
ppm

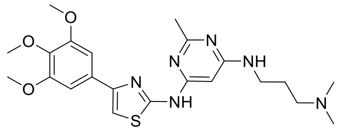
O
O
O
N
S
NH
N
N
NH
N

4m

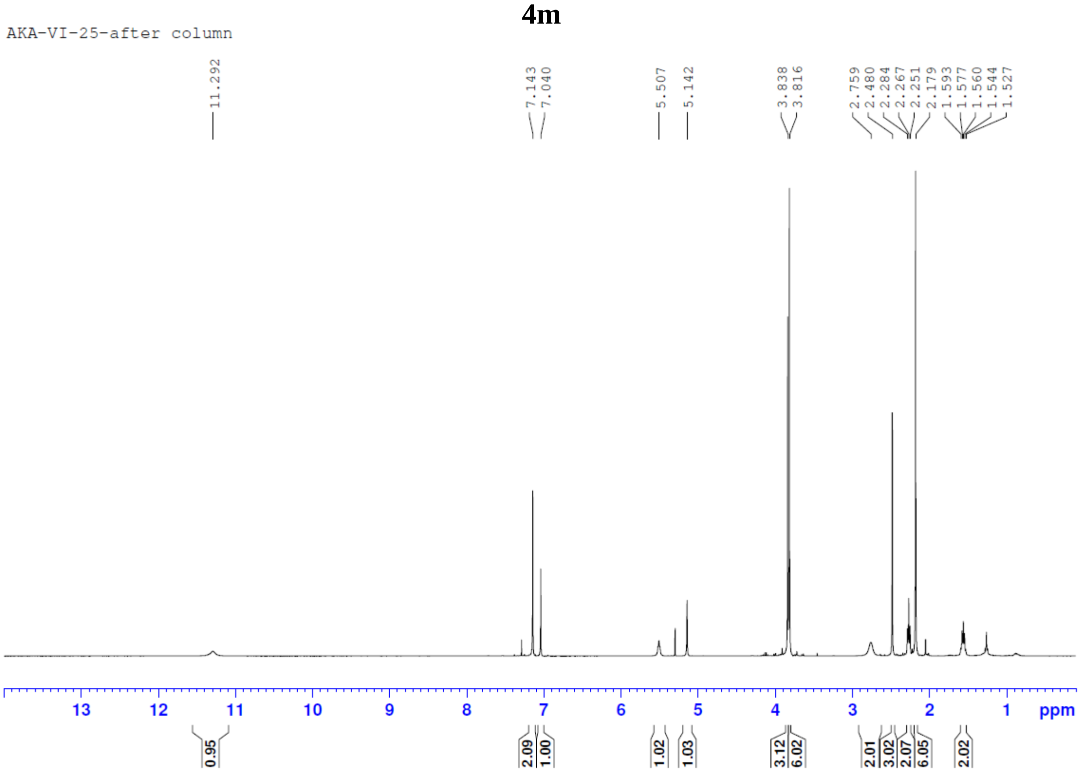
AKA-VI-25-after column
11.292
7.143
7.040
5.507
5.142
3.838
3.816
2.759
2.480
2.284
2.267
2.251
2.179
1.593
1.577
1.560
1.544
1.527
13 12 11 10 9 8 7 6 5 4 3 2 1 ppm
0.95
2.09
1.00
1.02
1.03
3.12
6.02
2.01
3.02
2.07
6.05
2.02

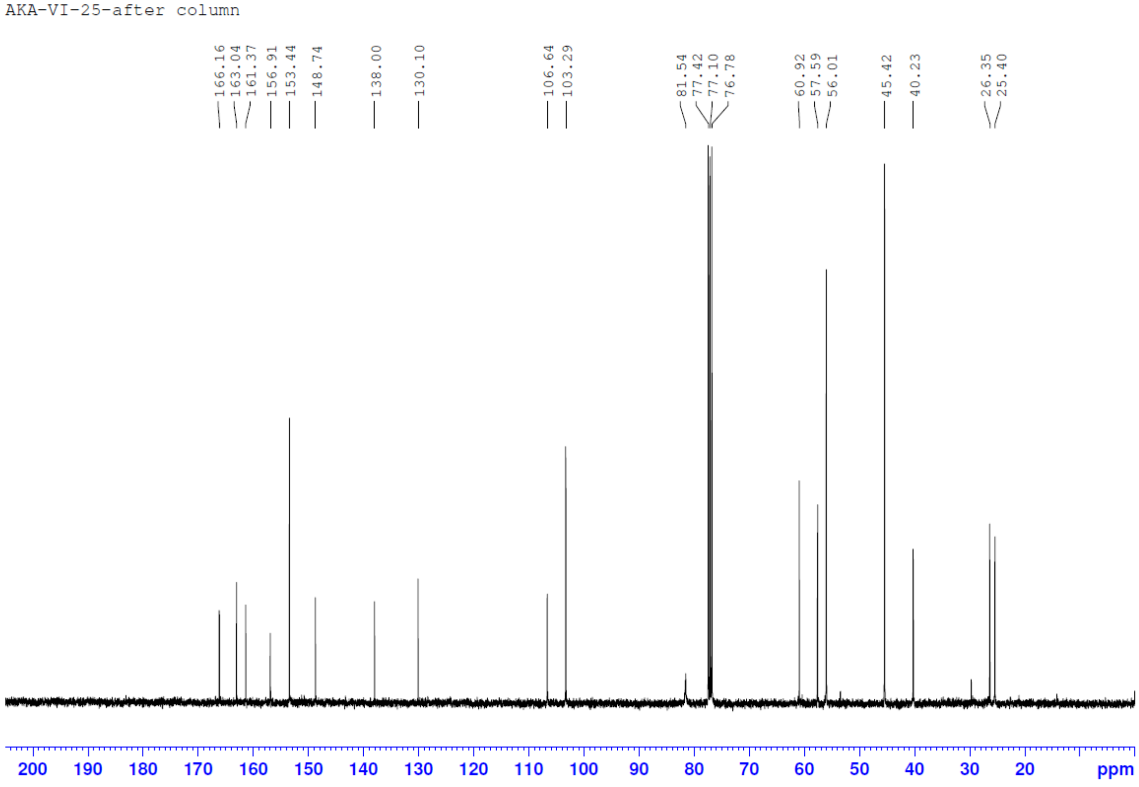
AKA-VI-25-after column
166.16
163.04
161.37
156.91
153.44
148.74
138.00
130.10
106.64
103.29
81.54
77.42
77.10
76.78
60.92
57.59
56.01
45.42
40.23
26.35
25.40
200 190 180 170 160 150 140 130 120 110 100 90 80 70 60 50 40 30 20 ppm

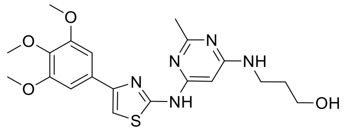

4n

AKA-VI-23-check for proton

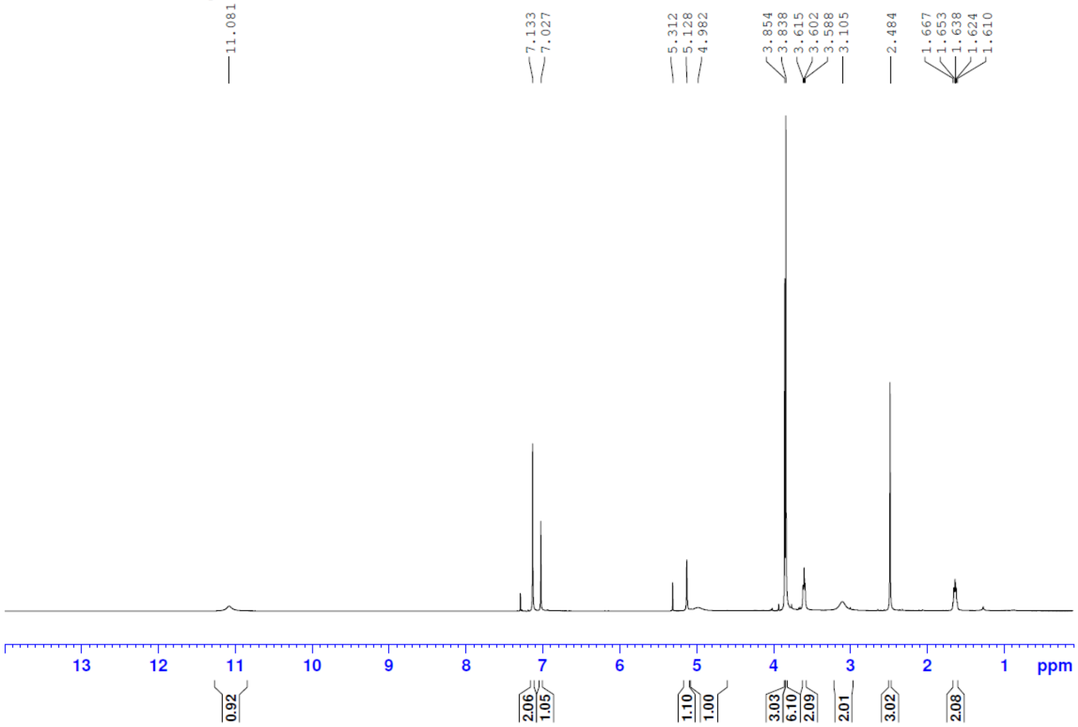

AKA-VI-23-check for carbon

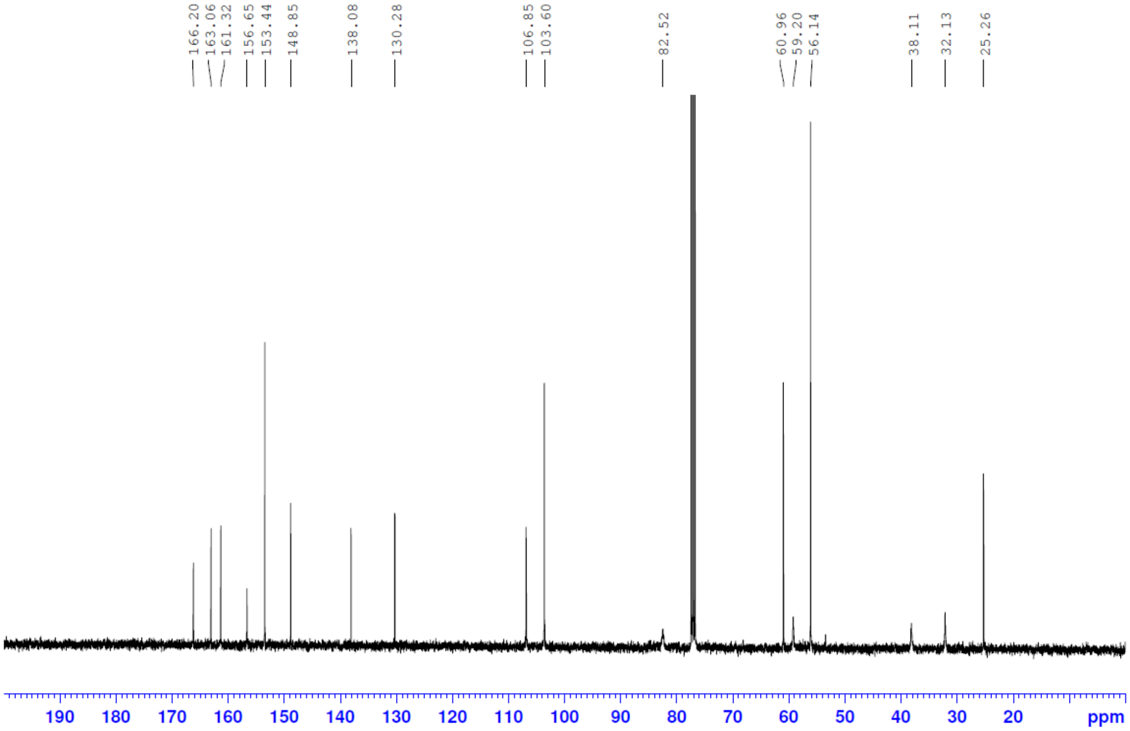

Figure S2. HRMS charts.

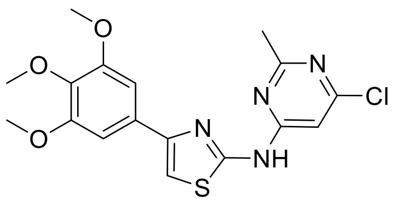

3

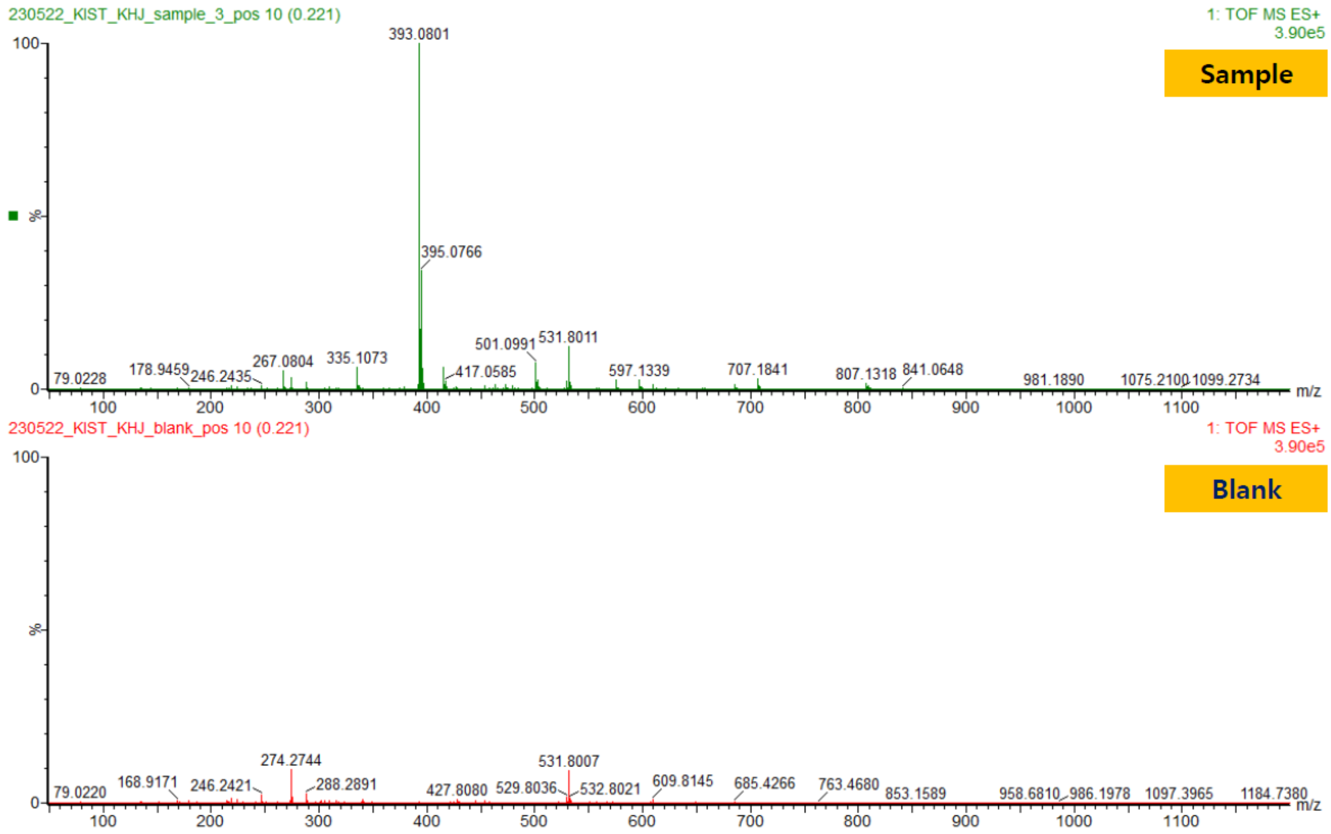

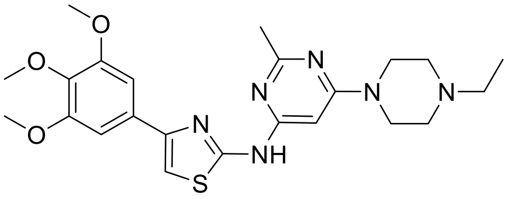

4a

Mass Spectrum: JHW-I-20 (Positive mode)

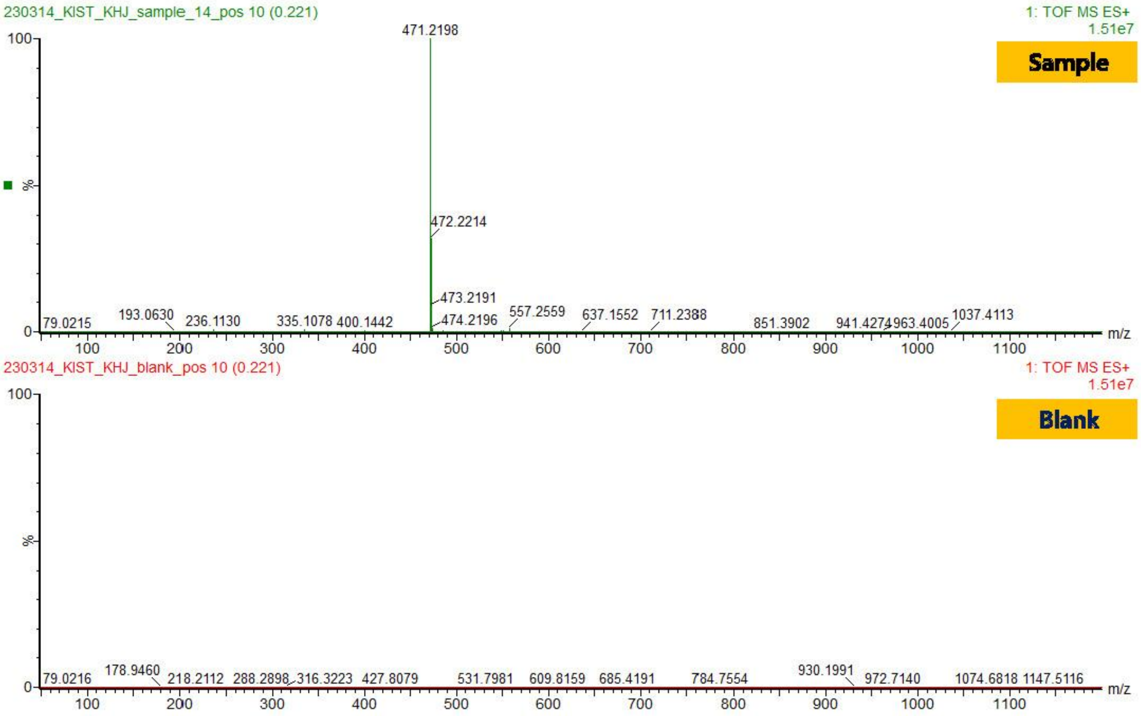

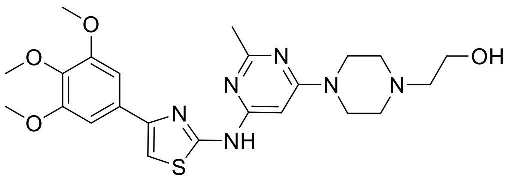

4b

Mass Spectrum: JHW-I-7 (Positive mode)

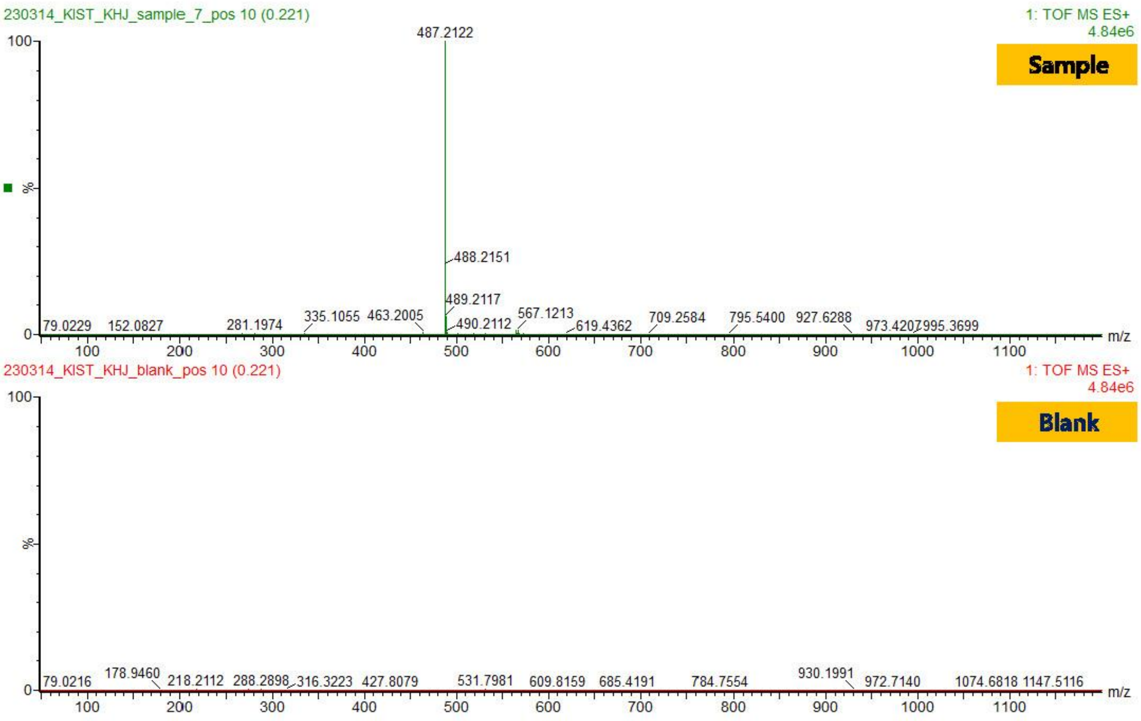

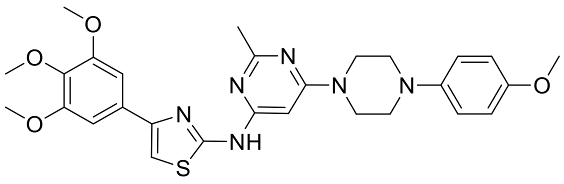

4c

Mass Spectrum: JHW-I-2 (Positive mode)

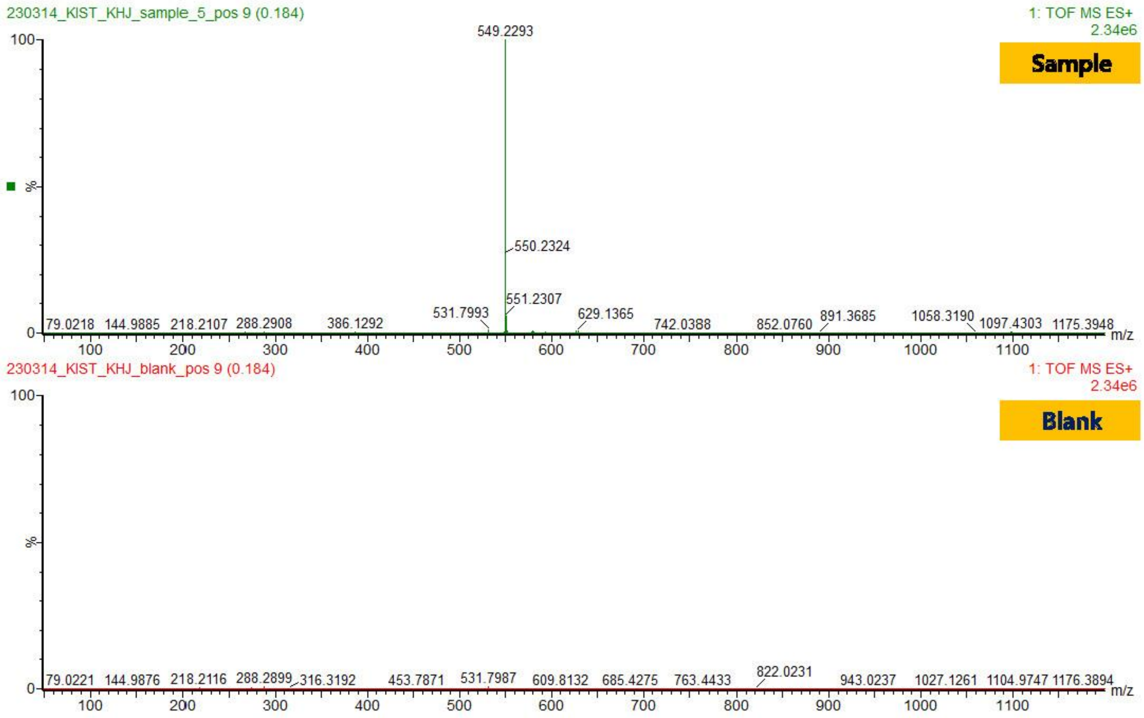

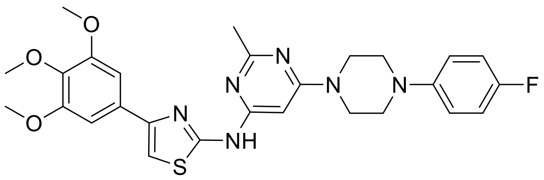

4d

Mass Spectrum: JHW-I-10 (Positive mode)

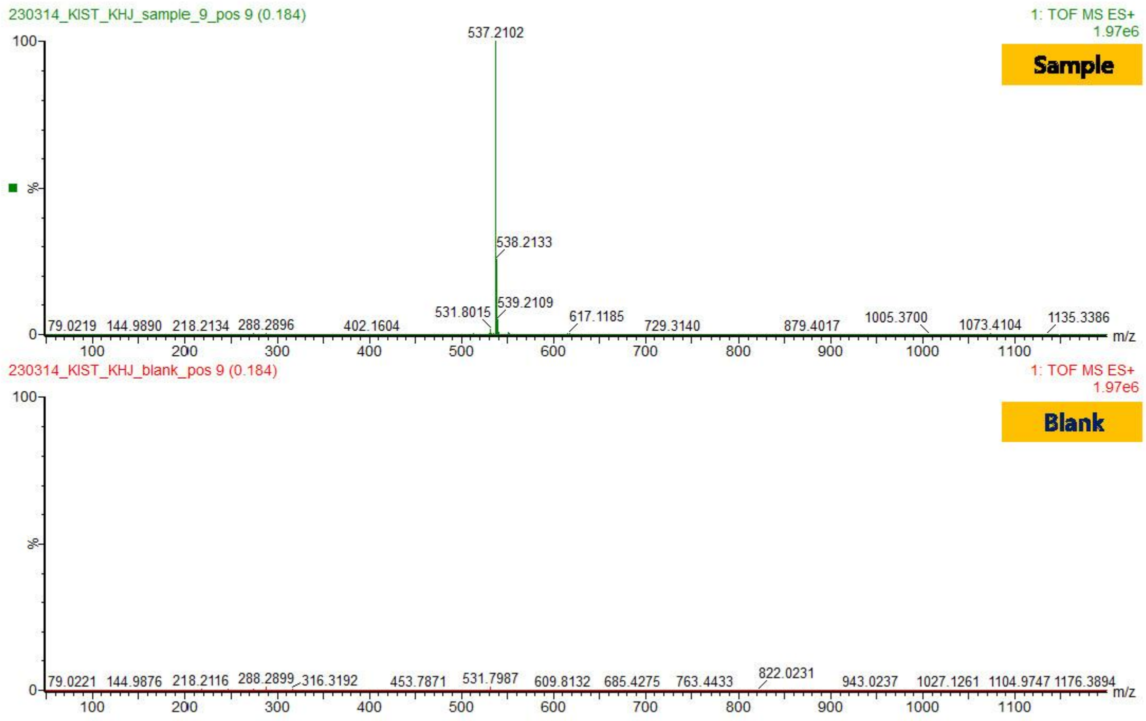

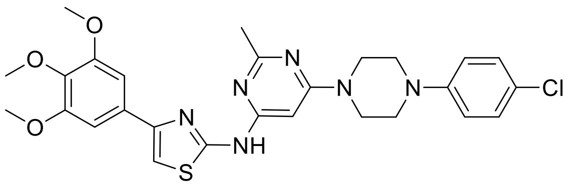

4e

Mass Spectrum: JHW-I-12 (Positive mode)

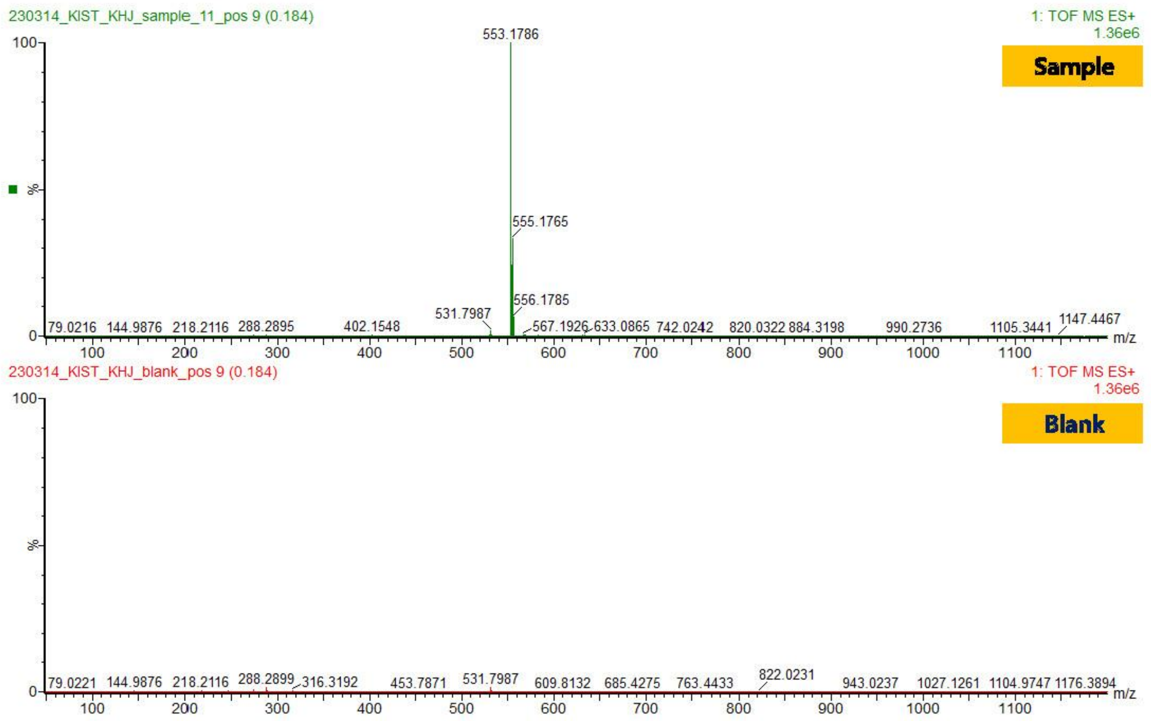

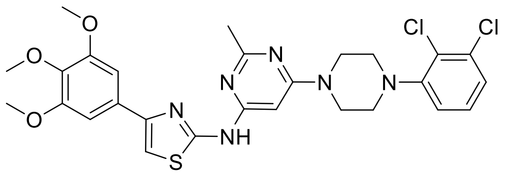

4f

Mass Spectrum: JHW-I-11 (Positive mode)

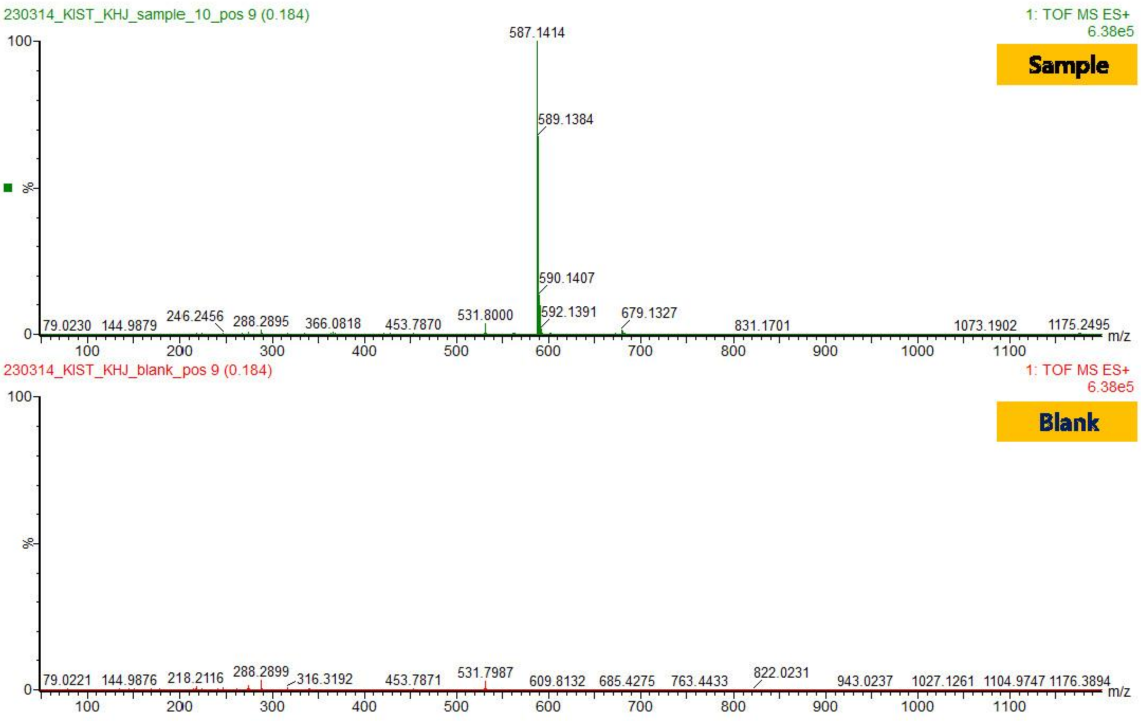

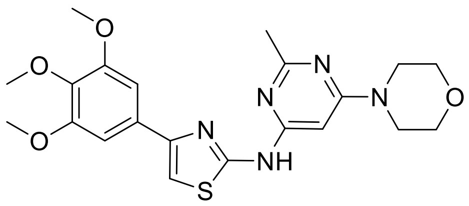

4g

Mass Spectrum: JHW-I-18 (Positive mode)

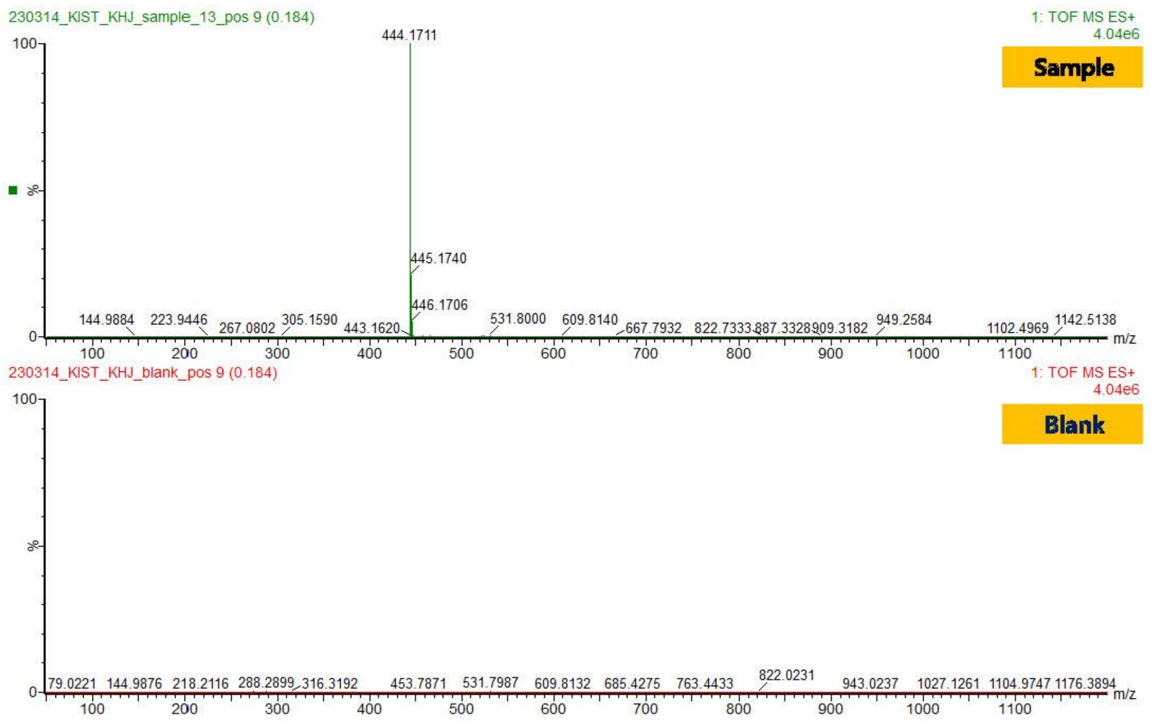

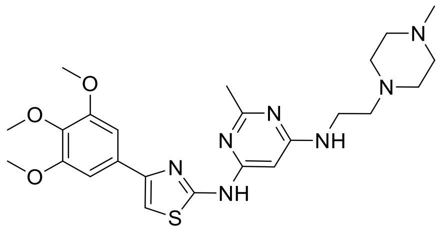

4h

Mass Spectrum: JHW-I-8 (Positive mode)

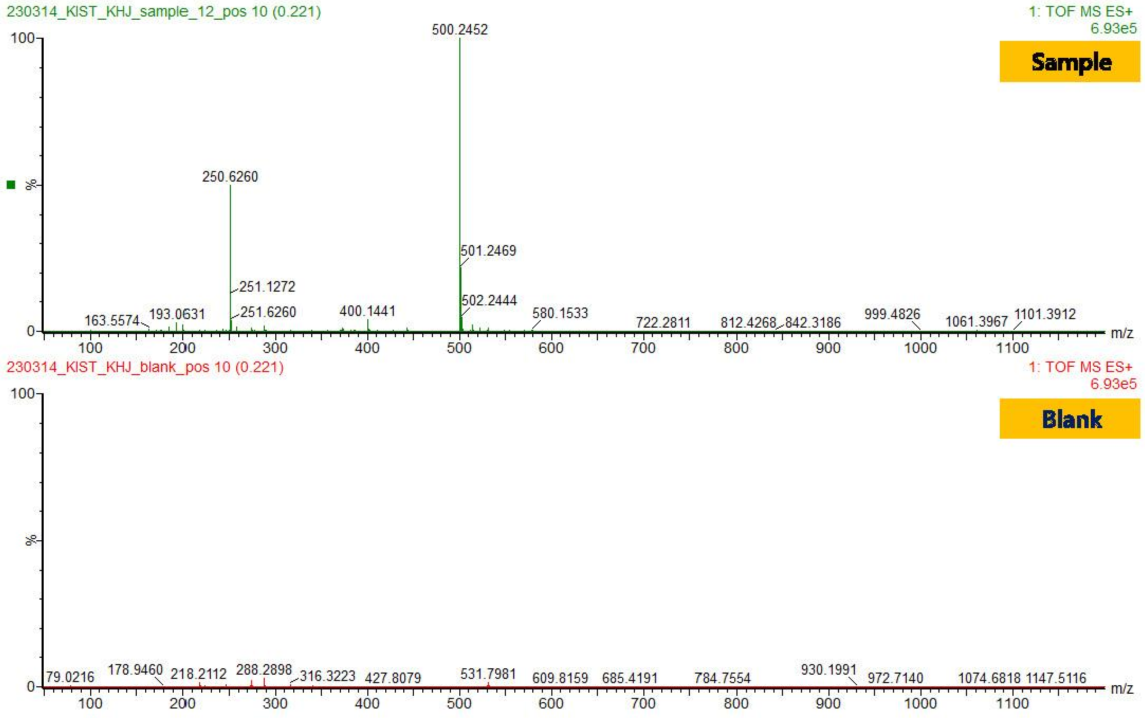

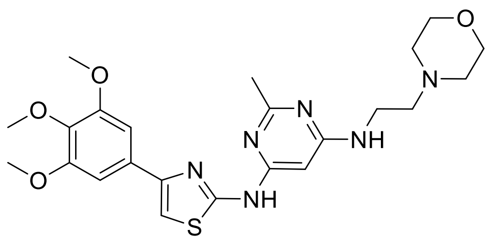

4i

Mass Spectrum: JHW-I-22 (Positive mode)

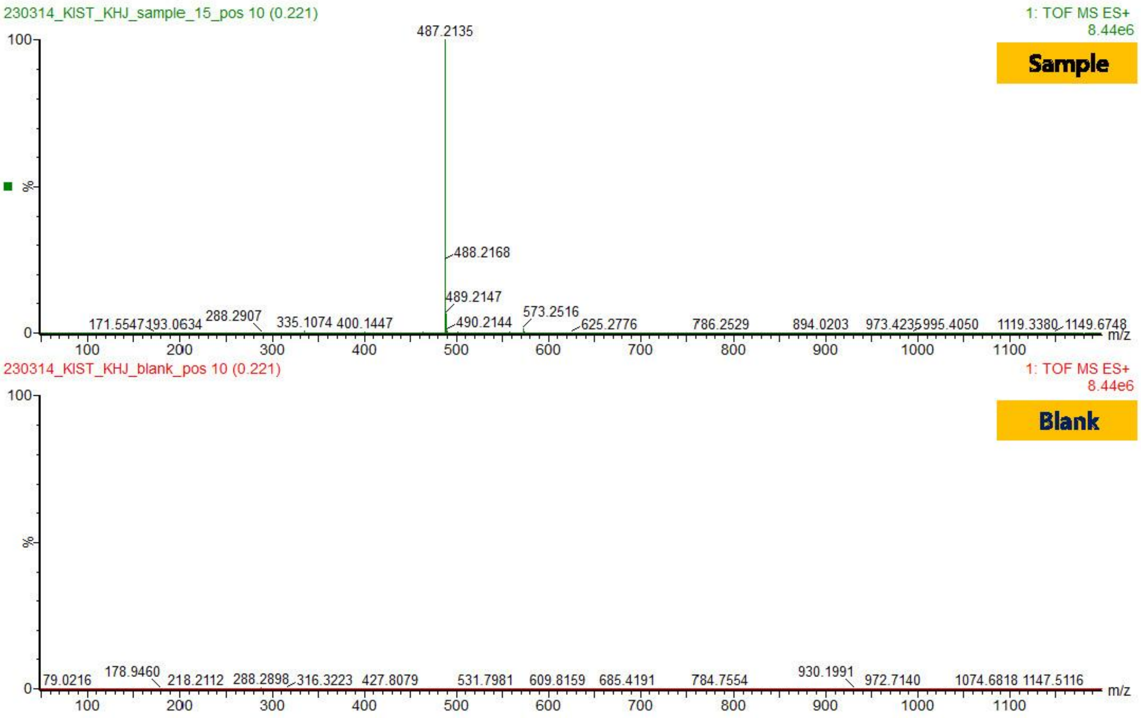

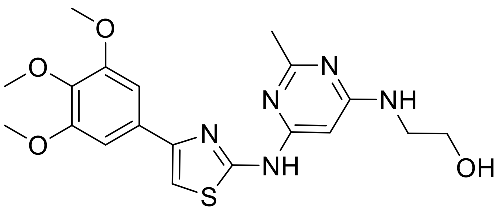

4j

Mass Spectrum: JHW-I-19 (Positive mode)

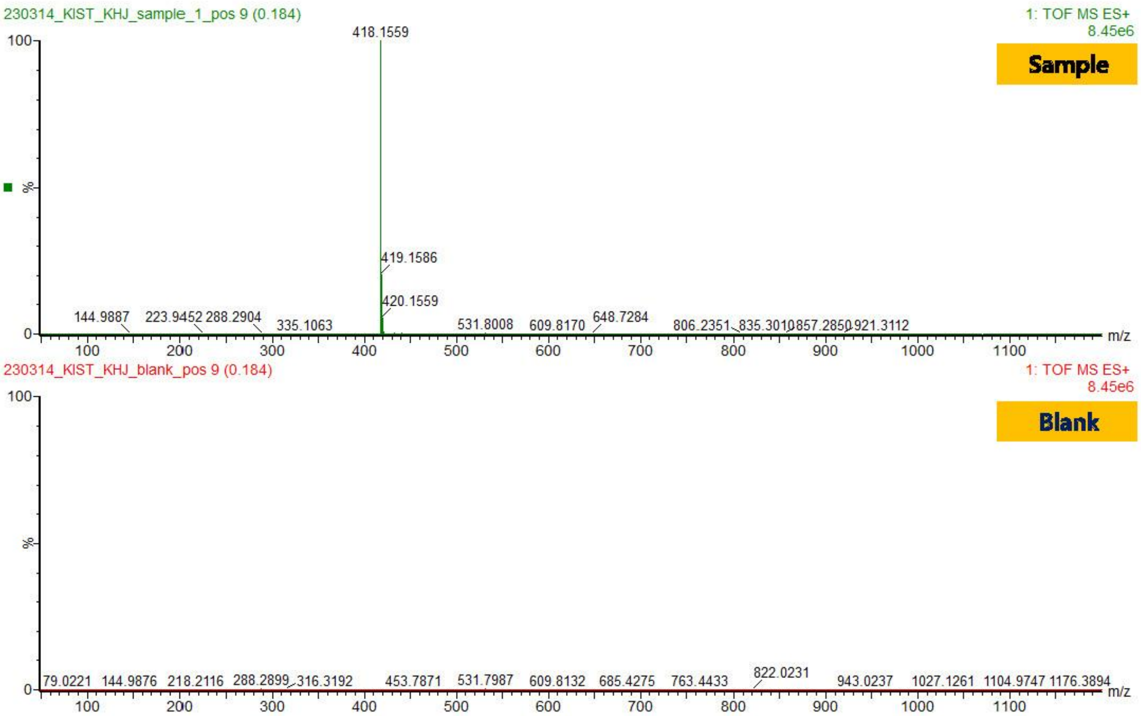

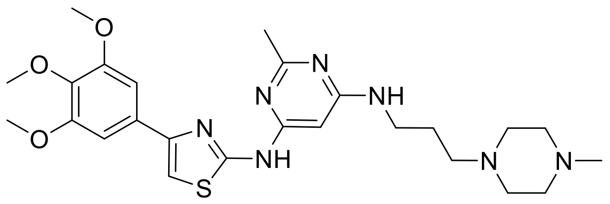

4k

Mass Spectrum: JHW-I-101 (Positive mode)

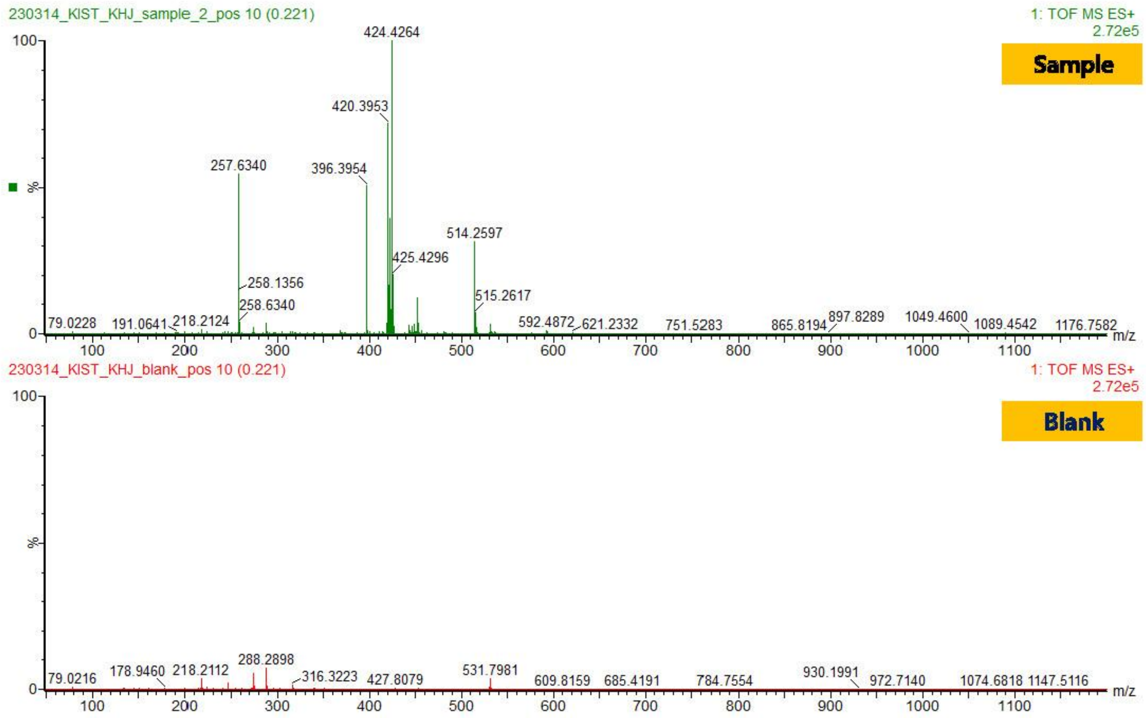

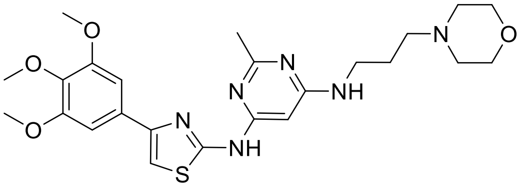

4l

Mass Spectrum: JHW-I-6 (Positive mode)

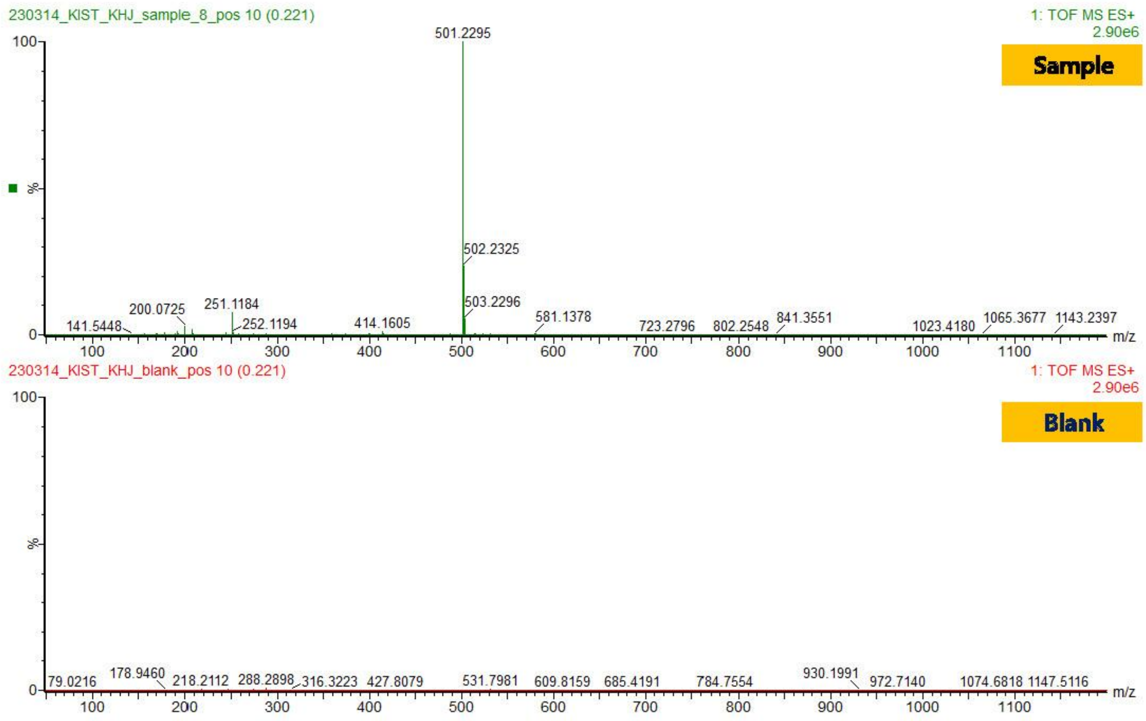

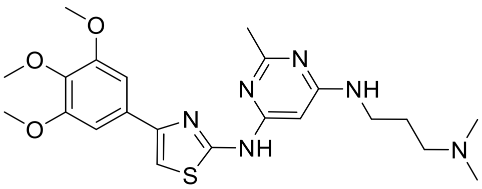

4m

Mass Spectrum: JHW-I-0 (Positive mode)

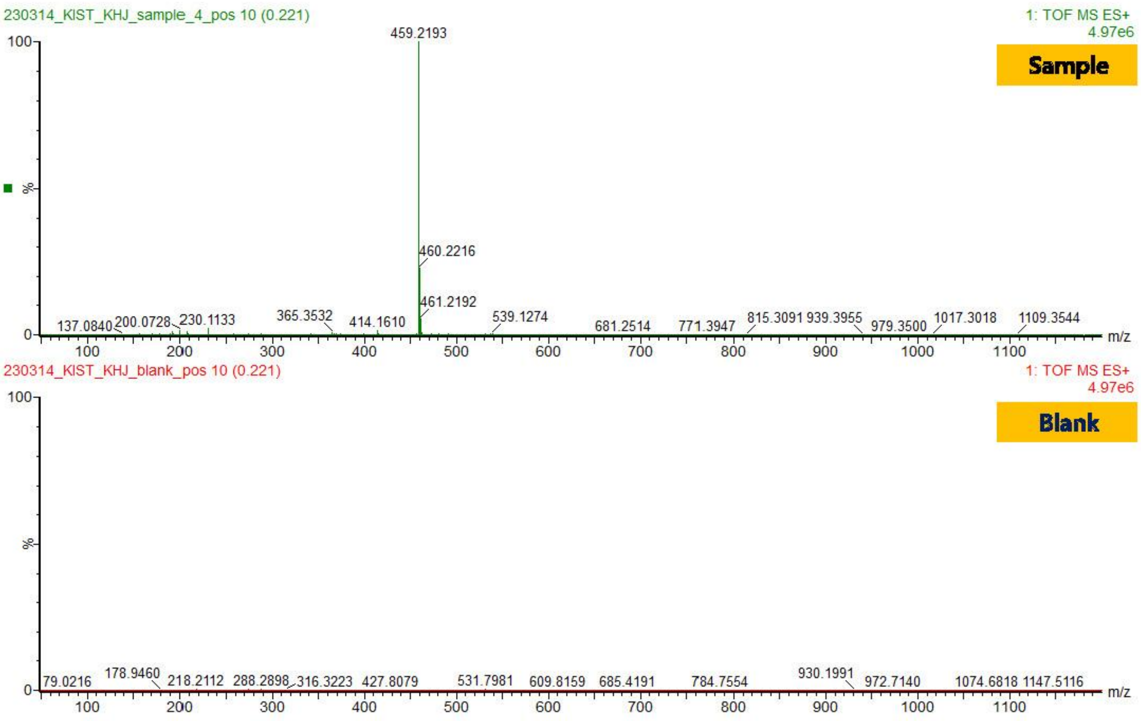

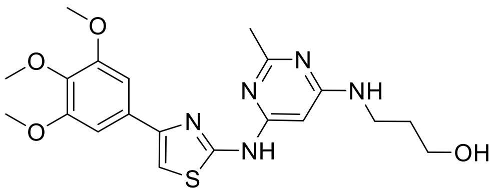

4n

Mass Spectrum: JHW-I-102 (Positive mode)

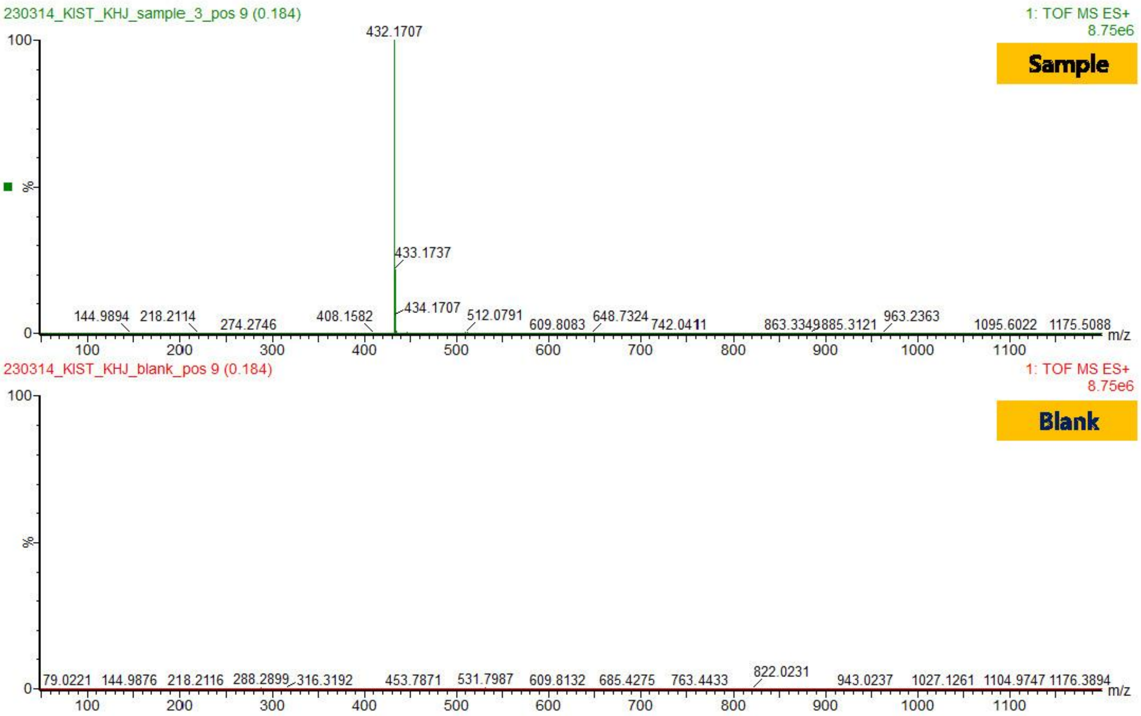

Figure S3. HPLC chromatograms.

4a

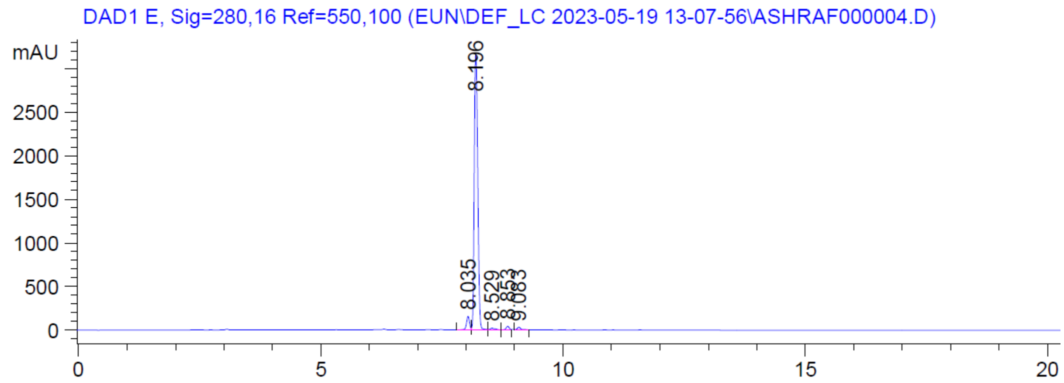

Peak #	RetTime [min]	Type	Width [min]	Area [mAU*s]	Height [mAU]	Area %
1	8.035	MM	0.0577	457.36737	132.04756	2.7660
2	8.196	MM	0.0832	1.57902e4	3164.52417	95.4942
3	8.529	MM	0.0661	63.35807	15.98133	0.3832
4	8.853	MM	0.0624	130.91321	34.96431	0.7917
5	9.083	MM	0.0629	93.40739	24.75652	0.5649

4b

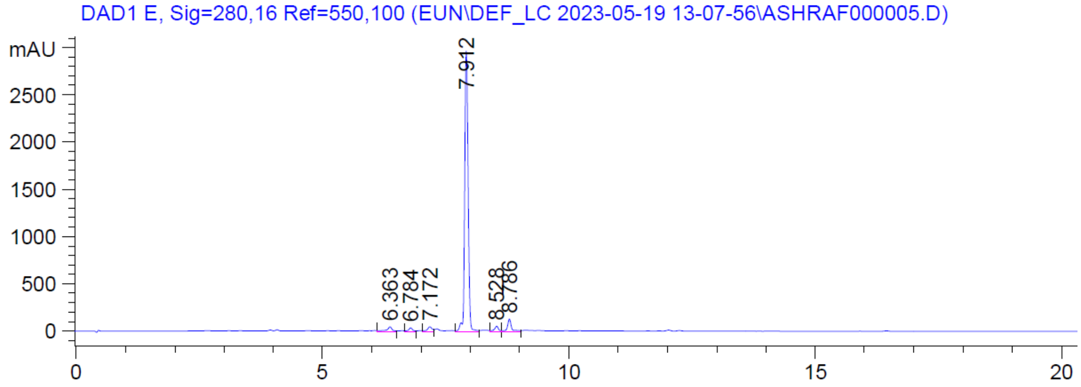

Peak #	RetTime [min]	Type	Width [min]	Area [mAU*s]	Height [mAU]	Area %
1	6.363	MM	0.0579	104.45980	30.07361	0.6206
2	6.784	MM	0.0617	105.35685	28.43876	0.6260
3	7.172	MM	0.1036	201.37787	32.40592	1.1964
4	7.912	MM	0.0880	1.59298e4	3017.06812	94.6430
5	8.528	MM	0.0591	133.52155	37.67151	0.7933
6	8.786	MM	0.0581	356.94733	102.36401	2.1207

4c

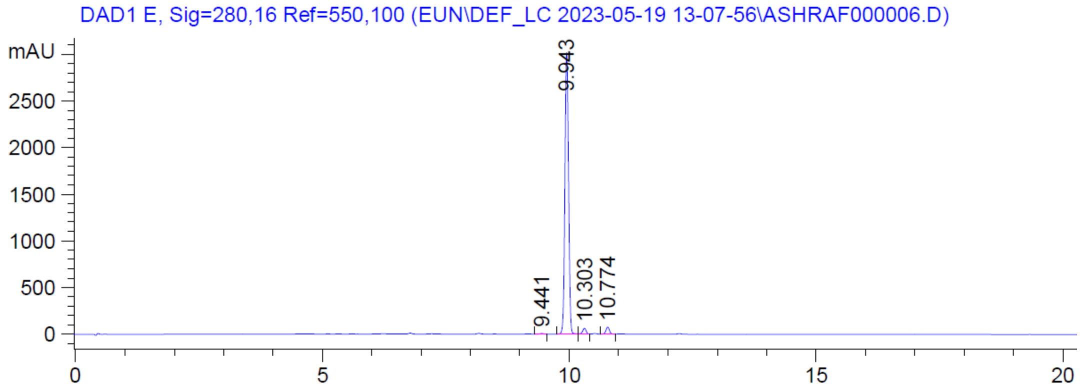

Peak #	RetTime [min]	Type	Width [min]	Area [mAU*s]	Height [mAU]	Area %
1	9.441	MM	0.0531	16.21639	5.09241	0.1000
2	9.943	MM	0.0861	1.56719e4	3035.16284	96.6872
3	10.303	MM	0.0711	240.45490	56.35232	1.4835
4	10.774	MM	0.0697	280.30371	67.05331	1.7293

4d

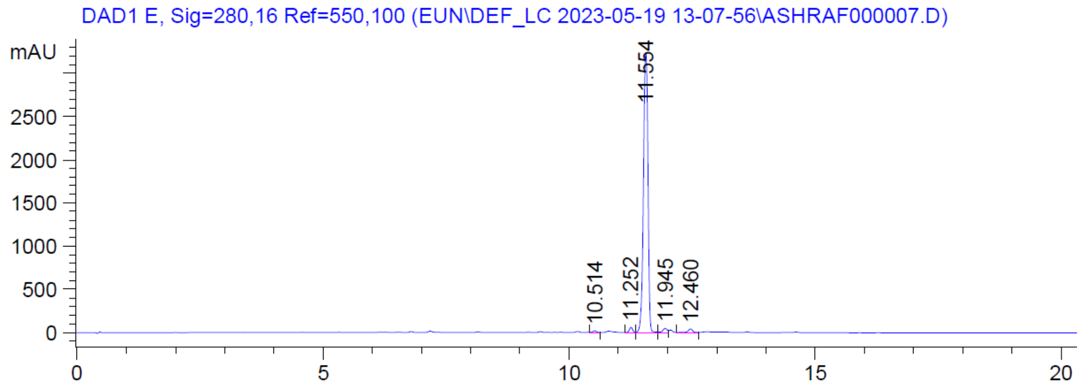

Peak #	RetTime [min]	Type	Width [min]	Area [mAU*s]	Height [mAU]	Area %
1	10.514	MM	0.0753	77.76376	17.22113	0.3557
2	11.252	MM	0.0665	216.86659	54.37672	0.9920
3	11.554	MM	0.1083	2.11809e4	3259.49268	96.8853
4	11.945	MM	0.1191	256.63257	35.92110	1.1739
5	12.460	MM	0.0647	129.67278	33.39864	0.5931

4e

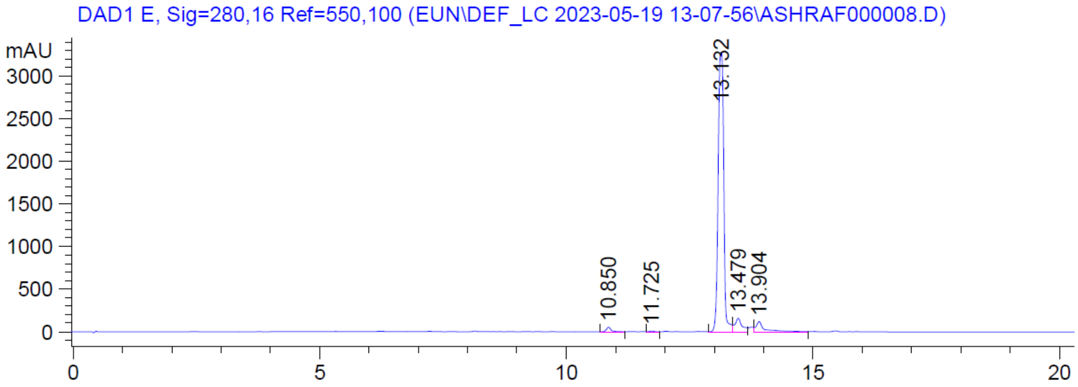

Peak #	RetTime [min]	Type	Width [min]	Area [mAU*s]	Height [mAU]	Area %
1	10.850	MM	0.1166	499.76828	71.45486	1.7694
2	11.725	MM	0.0707	96.67144	22.77393	0.3423
3	13.132	MM	0.1342	2.66627e4	3310.72241	94.3956
4	13.479	MM	0.0859	480.88800	93.28278	1.7025
5	13.904	MM	0.0964	505.66022	87.38710	1.7902

4f

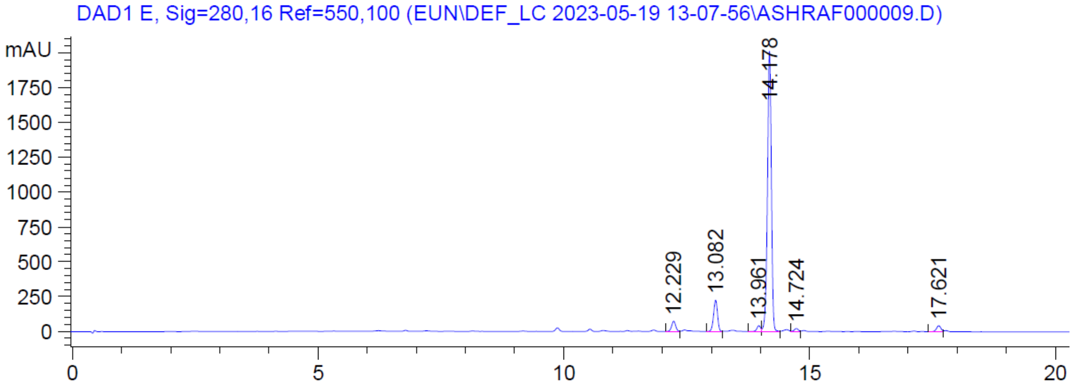

Peak #	RetTime [min]	Type	Width [min]	Area [mAU*s]	Height [mAU]	Area %
1	12.229	MM	0.0822	322.45212	65.40206	2.3795
2	13.082	MM	0.0871	1124.90076	215.34500	8.3011
3	13.961	MM	0.0571	78.17709	22.80712	0.5769
4	14.178	MM	0.0961	1.17756e4	2041.36389	86.8967
5	14.724	MM	0.0714	70.45380	16.43960	0.5199
6	17.621	MM	0.0884	179.66888	33.85971	1.3259

4g

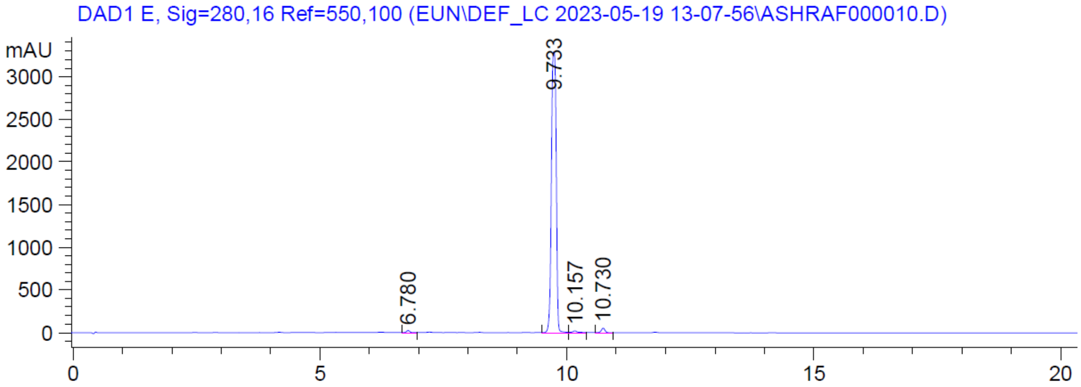

Peak #	RetTime [min]	Type	Width [min]	Area [mAU*s]	Height [mAU]	Area %
1	6.780	MM	0.0608	79.60249	21.80945	0.3454
2	9.733	MM	0.1142	2.26509e4	3305.02319	98.2748
3	10.157	MM	0.0773	76.37163	16.46140	0.3314
4	10.730	MM	0.0786	241.64787	51.23416	1.0484

4h

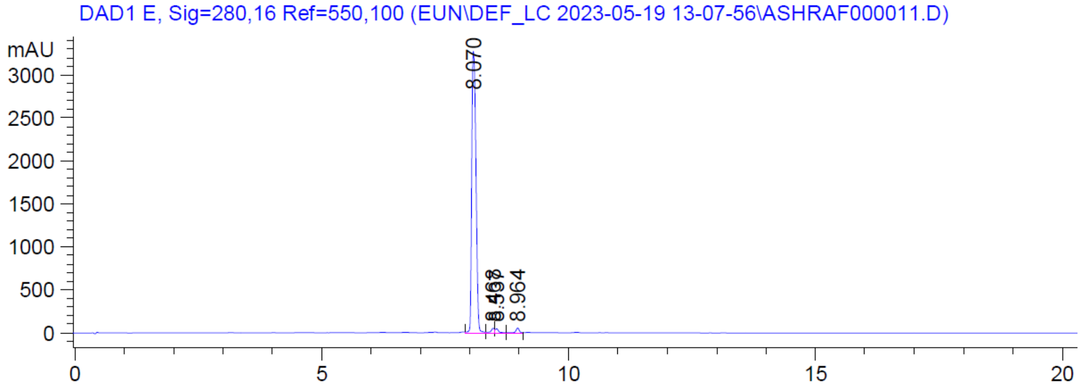

Peak #	RetTime [min]	Type	Width [min]	Area [mAU*s]	Height [mAU]	Area %
1	8.070	MM	0.1006	1.99534e4	3306.17407	97.5942
2	8.468	MM	0.0707	192.10826	45.29160	0.9396
3	8.537	MM	0.0603	148.93129	41.17998	0.7284
4	8.964	MM	0.0566	150.83940	44.44966	0.7378

Figure S4. NCI-60 cell line screening results.

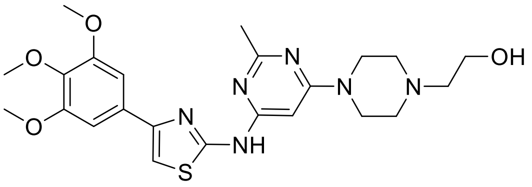

4b

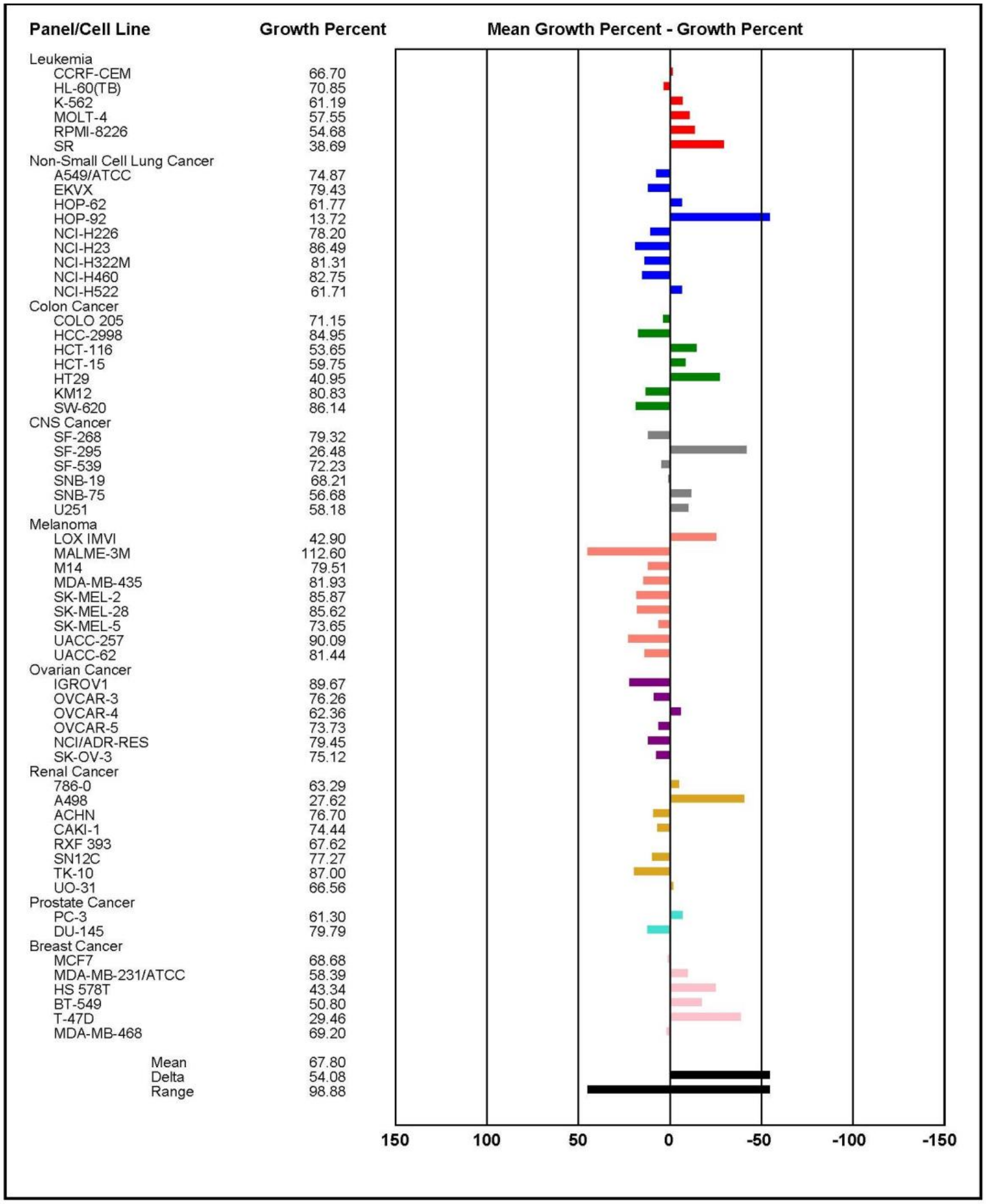

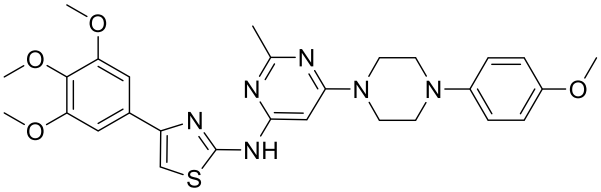

4c

Panel/Cell Line	Growth Percent
Leukemia	
HL-60(TB)	70.79
K-562	61.40
MOLT-4	47.42
SR	77.35
Non-Small Cell Lung Cancer	
A549/ATCC	72.14
EKVX	73.61
HOP-62	88.05
NCI-H226	70.69
NCI-H23	62.60
NCI-H322M	95.28
NCI-H460	86.14
NCI-H522	39.58
Colon Cancer	
COLO 205	94.73
HCC-2998	92.16
HCT-116	60.65
HCT-15	74.06
HT29	64.66
KM12	88.89
SW-620	93.67
CNS Cancer	
SF-268	94.76
SF-295	84.65
SF-539	84.05
SNB-19	90.98
SNB-75	82.52
U251	87.86
Melanoma	
LOX IMVI	82.25
MALME-3M	80.16
M14	86.57
MDA-MB-435	92.14
SK-MEL-2	78.28
SK-MEL-28	86.35
SK-MEL-5	39.99
UACC-257	54.93
UACC-62	67.01
Ovarian Cancer	
IGROV1	70.53
OVCAR-3	89.23
OVCAR-4	80.31
OVCAR-5	88.20
NCI/ADR-RES	88.54
SK-OV-3	96.62
Renal Cancer	
786-0	99.64
A498	75.06
ACHN	79.43
CAKI-1	65.75
RXF 393	109.35
SN12C	81.29
TK-10	87.47
UO-31	60.07
Prostate Cancer	
DU-145	88.42
Breast Cancer	
MCF7	79.77
MDA-MB-231/ATCC	90.80
HS 578T	108.98
BT-549	86.61
T-47D	37.02
MDA-MB-468	51.26
Mean	78.56
Delta	41.54
Range	72.33

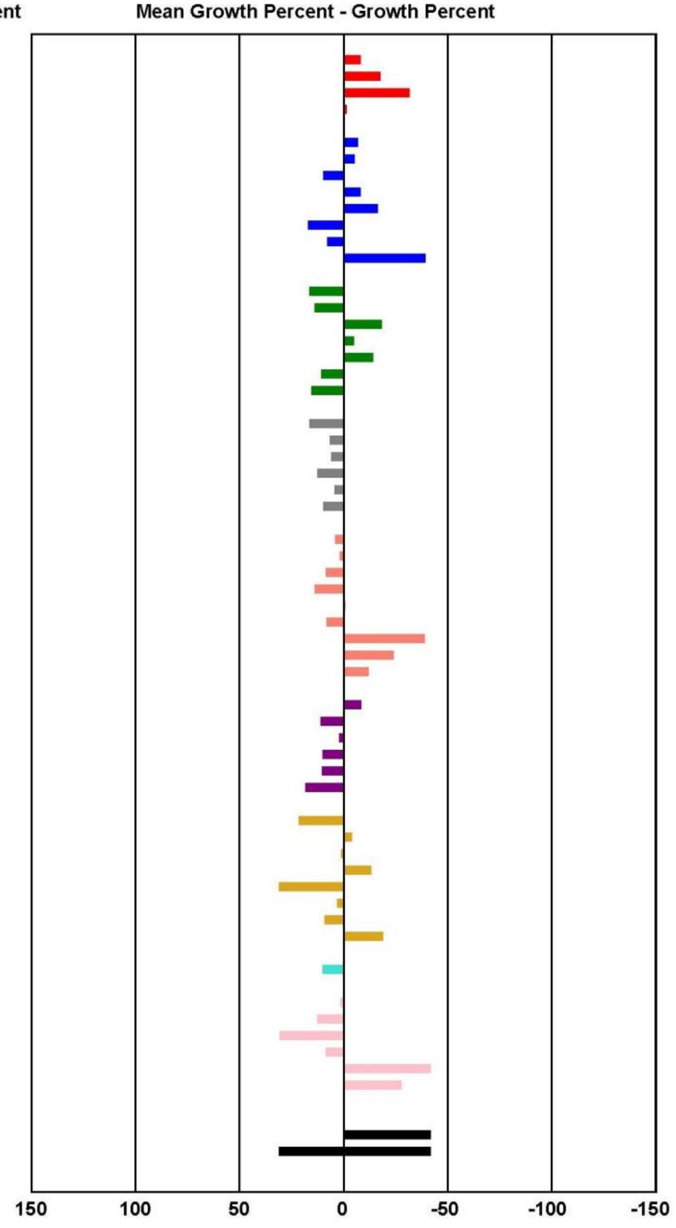

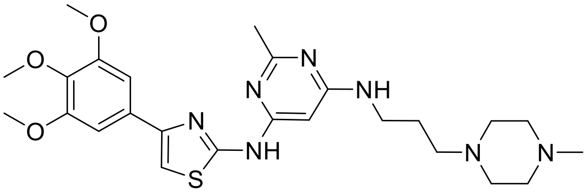

4k

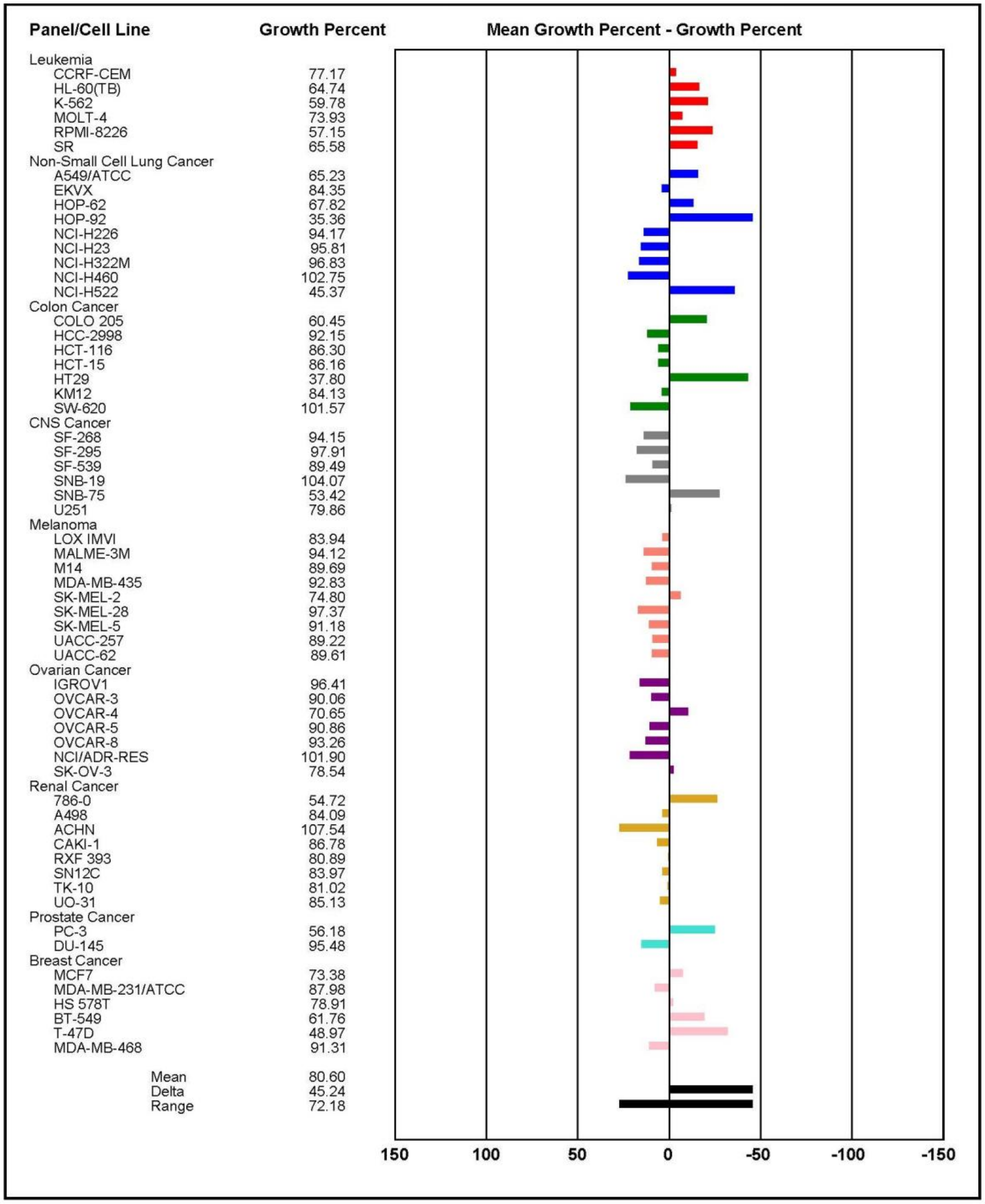
Panel/Cell Line
Growth Percent
Mean Growth Percent - Growth Percent
Leukemia
CCRF-CEM 77.17
HL-60(TB) 64.74
K-562 59.78
MOLT-4 73.93
RPMI-8226 57.15
SR 65.58
Non-Small Cell Lung Cancer
A549/ATCC 65.23
EKVX 84.35
HOP-62 67.82
HOP-92 35.36
NCI-H226 94.17
NCI-H23 95.81
NCI-H322M 96.83
NCI-H460 102.75
NCI-H522 45.37
Colon Cancer
COLO 205 60.45
HCC-2998 92.15
HCT-116 86.30
HCT-15 86.16
HT29 37.80
KM12 84.13
SW-620 101.57
CNS Cancer
SF-268 94.15
SF-295 97.91
SF-539 89.49
SNB-19 104.07
SNB-75 53.42
U251 79.86
Melanoma
LOX IMVI 83.94
MALME-3M 94.12
M14 89.69
MDA-MB-435 92.83
SK-MEL-2 74.80
SK-MEL-28 97.37
SK-MEL-5 91.18
UACC-257 89.22
UACC-62 89.61
Ovarian Cancer
IGROV1 96.41
OVCAR-3 90.06
OVCAR-4 70.65
OVCAR-5 90.86
OVCAR-8 93.26
NCI/ADR-RES 101.90
SK-OV-3 78.54
Renal Cancer
786-0 54.72
A498 84.09
ACHN 107.54
CAKI-1 86.78
RXF 393 80.89
SN12C 83.97
TK-10 81.02
UO-31 85.13
Prostate Cancer
PC-3 56.18
DU-145 95.48
Breast Cancer
MCF7 73.38
MDA-MB-231/ATCC 87.98
HS 578T 78.91
BT-549 61.76
T-47D 48.97
MDA-MB-468 91.31
Mean 80.60
Delta 45.24
Range 72.18
150
100
50
0
-50
-100
-150

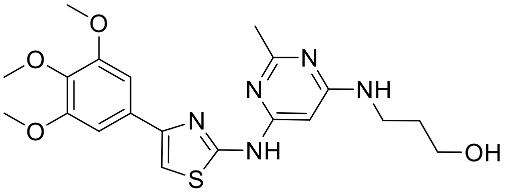

4n

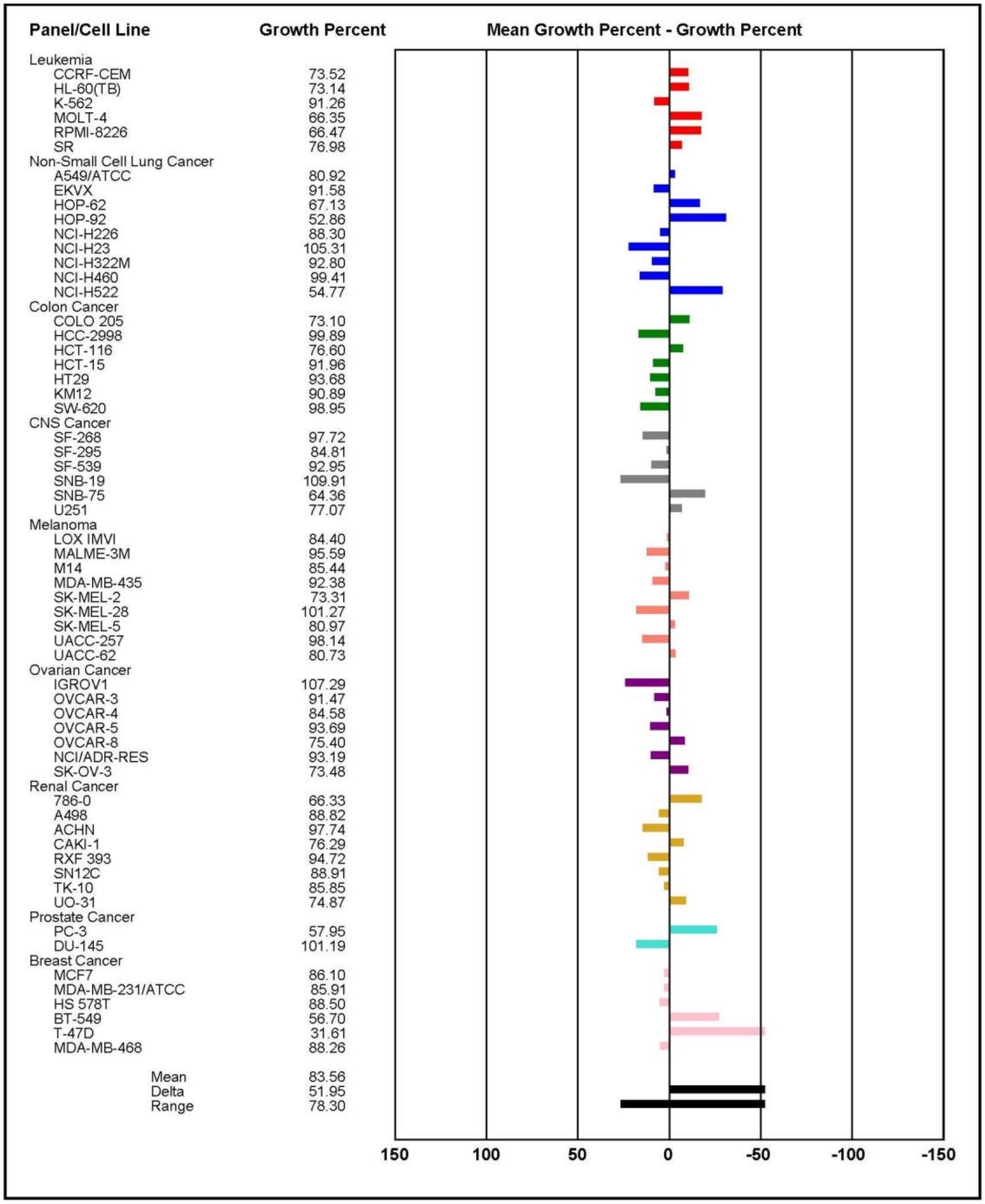
Panel/Cell Line
Growth Percent
Mean Growth Percent - Growth Percent
Leukemia
CCRF-CEM 73.52
HL-60(TB) 73.14
K-562 91.26
MOLT-4 66.35
RPMI-8226 66.47
SR 76.98
Non-Small Cell Lung Cancer
A549/ATCC 80.92
EKVX 91.58
HOP-62 67.13
HOP-92 52.86
NCI-H226 88.30
NCI-H23 105.31
NCI-H322M 92.80
NCI-H460 99.41
NCI-H522 54.77
Colon Cancer
COLO 205 73.10
HCC-2998 99.89
HCT-116 76.60
HCT-15 91.96
HT29 93.68
KM12 90.89
SW-620 98.95
CNS Cancer
SF-268 97.72
SF-295 84.81
SF-539 92.95
SNB-19 109.91
SNB-75 64.36
U251 77.07
Melanoma
LOX IMVI 84.40
MALME-3M 95.59
M14 85.44
MDA-MB-435 92.38
SK-MEL-2 73.31
SK-MEL-28 101.27
SK-MEL-5 80.97
UACC-257 98.14
UACC-62 80.73
Ovarian Cancer
IGROV1 107.29
OVCAR-3 91.47
OVCAR-4 84.58
OVCAR-5 93.69
OVCAR-8 75.40
NCI/ADR-RES 93.19
SK-OV-3 73.48
Renal Cancer
786-0 66.33
A498 88.82
ACHN 97.74
CAKI-1 76.29
RXF 393 94.72
SN12C 88.91
TK-10 85.85
UO-31 74.87
Prostate Cancer
PC-3 57.95
DU-145 101.19
Breast Cancer
MCF7 86.10
MDA-MB-231/ATCC 85.91
HS 578T 88.50
BT-549 56.70
T-47D 31.61
MDA-MB-468 88.26
Mean 83.56
Delta 51.95
Range 78.30
150
100
50
0
-50
-100
-150
